# TDRD5 facilitates piRNA biogenesis and male fertility through phase separation

**DOI:** 10.1038/s44319-026-00890-6

**Published:** 2026-08-04

**Authors:** Wenyang Yu, Jie Gao, Guanyi Shang, Canmei Chen, Ting Zhao, Ke Wang, Miao Li, Na Jiang, Deqiang Ding

**Affiliations:** 1 Shanghai Key Laboratory of Maternal Fetal Medicine, Clinical and Translational Research Center, Shanghai First Maternity and Infant Hospital, Frontier Science Center for Stem Cell Research, School of Life Sciences and Technology, Tongji University, Shanghai, China; 2 Center for Reproductive Medicine, Shanghai First Maternity and Infant Hospital, School of Medicine, Tongji University, Shanghai, China; 3 Department of Nephrology, Ren Ji Hospital, Shanghai Jiao Tong University School of Medicine, Shanghai, China

**Keywords:** RNA Biology

## Abstract

piRNAs are small regulatory RNAs with critical roles in transposon silencing and regulation of gametogenesis. Phase separation is a driving force for piRNA biogenesis machinery formation; however, the specific piRNA-related proteins that mediate phase separation and how they coordinate to drive piRNA production remain incompletely understood. Through systematic screening, we show here that TDRD5 undergoes phase separation to promote piRNA biogenesis. TDRD5 condensation is mediated by its Lotus domains and an intrinsically disordered region enriched with aromatic residues. Furthermore, TDRD1 and TDRD5 colocalize and synergistically enhance each other’s phase separation. TDRD5 phase separation is required for intermitochondrial cement assembly and fetal piRNA processing in fetal/neonatal gonocytes but is dispensable for pachytene piRNA biogenesis in adult mice. TDRD5 phase separation deficiency impairs male fertility in mice and may contribute to human male infertility. Our findings highlight the critical but diverse contributions of phase-separated proteins to germ granule assembly and piRNA production.

## Synopsis

TDRD5 undergoes phase separation to promote intermitochondrial cement assembly and piRNA biogenesis, which is important for transposon silencing and male fertility in mice.

- TDRD5 phase separation is driven by its Lotus domains and an intrinsically disordered region enriched with aromatic residues.
- TDRD1 and TDRD5 colocalize and synergistically enhance each other’s condensation.
- TDRD5 phase separation is required for intermitochondrial cement assembly and fetal piRNA processing, but not for pachytene piRNA biogenesis.

## Abstract (web summary)

TDRD5 undergoes phase separation to promote intermitochondrial cement assembly and piRNA biogenesis, which is important for transposon silencing and male fertility in mice.

## Graphical abstract

**Figure:**
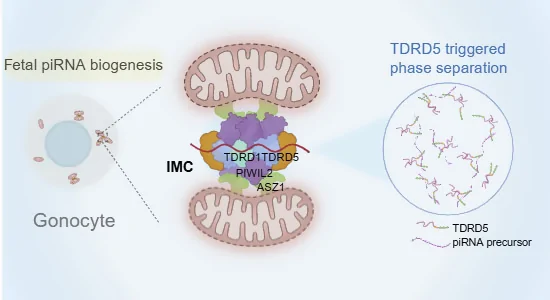

## Introduction

Piwi-interacting RNAs (piRNAs) are a class of germline-specific small non-coding RNAs essential for transposon silencing, germ cell development, and fertility maintenance (Ku and Lin, 2014; Ozata et al, 2019; Wang et al, 2022). Mature piRNAs are produced through endonucleolytic cleavage and stepwise trimming of long single-stranded precursor transcripts, a process mediated by a dedicated ribonucleoprotein complex (Gao et al, 2025). This machinery includes endonucleases, exonucleases, RNA helicases, scaffold proteins, and associated regulatory factors (Darricarrère et al, 2013; Ding et al, 2017; Zhang et al, 2016). The spatial organization and functional coordination of these components are crucial for catalytic efficiency, underscoring the importance of their precise assembly for robust piRNA biogenesis. Notably, germ granules serve as platforms for the piRNA pathway and appear to facilitate both piRNA biogenesis and the degradation of piRNA-targeted transcripts (Spichal et al, 2021; Thomas et al, 2023; Voronina et al, 2011).

In mice, piRNA biogenesis takes place within intermitochondrial cement (IMC), an electron-dense germ granule situated among clustered mitochondria in germ cells (Wang et al, 2022). Several IMC components, such as mitoPLD, ASZ1, and TDRKH, are anchored to mitochondria, while others are recruited through various mechanisms (Gao et al, 2025; Miao et al, 2025; Watanabe et al, 2011; Wei et al, 2024). PIWI proteins are thought to play a key role in tethering the piRNA biogenesis machinery to the mitochondrial outer membrane (Ding et al, 2019; Vourekas et al, 2012; Wei et al, 2023). Once localized, IMC components coordinate to assemble into a functional complex that drives efficient piRNA production. However, the specific roles of individual IMC components in IMC assembly and piRNA biogenesis remain poorly understood.

During murine germ cell development, there are two distinct modes of piRNA production: fetal piRNAs generated in gonocytes and pachytene piRNAs produced in pachytene spermatocytes (Aravin et al, 2009; Wang et al, 2022). Although both types of piRNAs are produced in the IMCs, their origins, biogenesis pathway and biological functions are significantly different. In gonocytes, an active ping-pong cycle drives the robust production of transposon-derived piRNAs, which suppress transposon activity primarily through DNA methylation and mRNA degradation (Czech and Hannon, 2016; Kuramochi-Miyagawa et al, 2008). In contrast, during pachytene piRNA biogenesis, the ping-pong cycle is largely suppressed, resulting in a minimal transposon-derived piRNA population (Xiong et al, 2023). Instead, the vast majority of pachytene piRNAs originate from piRNA clusters, though their precise biological roles remain poorly understood (Li et al, 2013). Notably, most piRNA-related proteins are expressed in both gonocytes and pachytene spermatocytes. Given the substantial differences between fetal and pachytene piRNAs, it is plausible that certain proteins may perform distinct functions during the two developmental stages.

Phase separation is a physical process through which a homogeneous solution spontaneously separates into distinct phases with differing compositions and properties (Alberti et al, 2019; Sawyer et al, 2019; Yang et al, 2020; Zhao and Zhang, 2020). This mechanism concentrates specific biomolecules and serves as a major driving force behind the formation of membrane-less organelles (Sawyer et al, 2019; Schmidt and Görlich, 2016; Zhao and Zhang, 2020). Recent studies have shown that phase separation governs the assembly of germ granules across species (Cipriani et al, 2021; Dodson and Kennedy, 2020). In mammalian germ cells, the IMC component TDRD1 promotes IMC assembly via phase separation, mediated by its tetrameric coiled-coil domain and dimethylarginine-binding Tudor domains (Gao et al, 2024). Given that IMCs contain numerous proteins, it remains unclear whether other IMC components also undergo phase separation and how they collectively contribute to IMC assembly. TDRD5 plays evolutionarily conserved roles in germ granule formation and piRNA biogenesis in germ cells (Yabuta et al, 2011). In *Drosophila*, TDRD5 homolog Tejas functions as a core component in nuage assembly through its intrinsically disordered region (Ding et al, 2018; Lin et al, 2023). In mammals, how TDRD5, as an IMC component, participates in IMC assembly and piRNA biogenesis remains largely unclear.

In this study, we systematically assessed condensate formation by piRNA-related proteins in cells and identified two Tudor domain proteins, TDRD5 and RNF17/TDRD4, that form prominent cytoplasmic granules, in addition to TDRD1. We further demonstrated that TDRD5 undergoes phase separation upon interaction with RNA G-quadruplex through its Lotus domains, and that an intrinsically disordered region (IDR4) is critical for phase separation. Aromatic residues within IDR4 play a key role in TDRD5 phase separation. Unlike TDRD1, the Tudor domain of TDRD5 appears dispensable for phase separation. We also found that TDRD1 and TDRD5 colocalize and mutually enhance their phase separation. Phase separation-deficient mutations in TDRD5 disrupted IMC assembly and piRNA biogenesis in gonocytes, leading to transposon derepression and reduced male fertility. Interestingly, pachytene piRNA biogenesis and IMC formation in pachytene spermatocytes were not markedly affected in TDRD5 phase separation-deficient mice. Together, our findings underscore the critical but diverse contribution of phase separation to germ granule assembly and piRNA production.

## Results

### TDRD5 assembles fluid-like macromolecular condensates in cells

**Figure 1:**
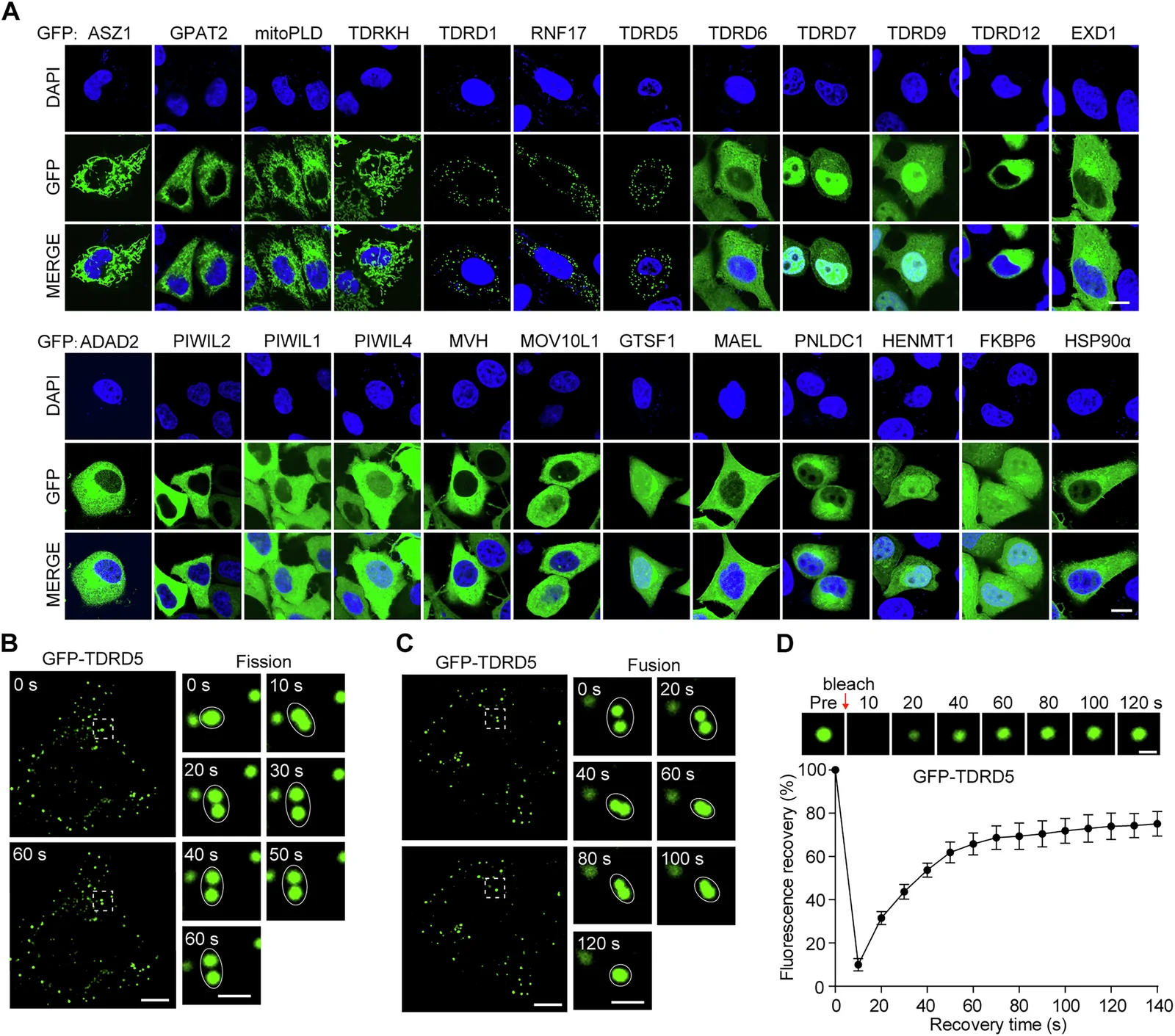
TDRD5 assembles fluid-like macromolecular condensates in cells. (**A**) Images of HeLa cells transfected with indicated plasmid. Scale bars, 10 μm. (**B**, **C**) Live-cell images showing the fission (**B**) and fusion (**C**) of TDRD5 condensates in HeLa cells transfected with GFP-TDRD5. Scale bar, 10 μm (left) and 2 μm (right). (**D**) FRAP analysis of GFP-TDRD5 in transfected HeLa cells. Quantification of FRAP data is shown below. *n* = 5 droplets. The data represent the mean ± s.e.m. Scale bar, 1 μm. Source data are available online for this figure.

**Figure EV1:**
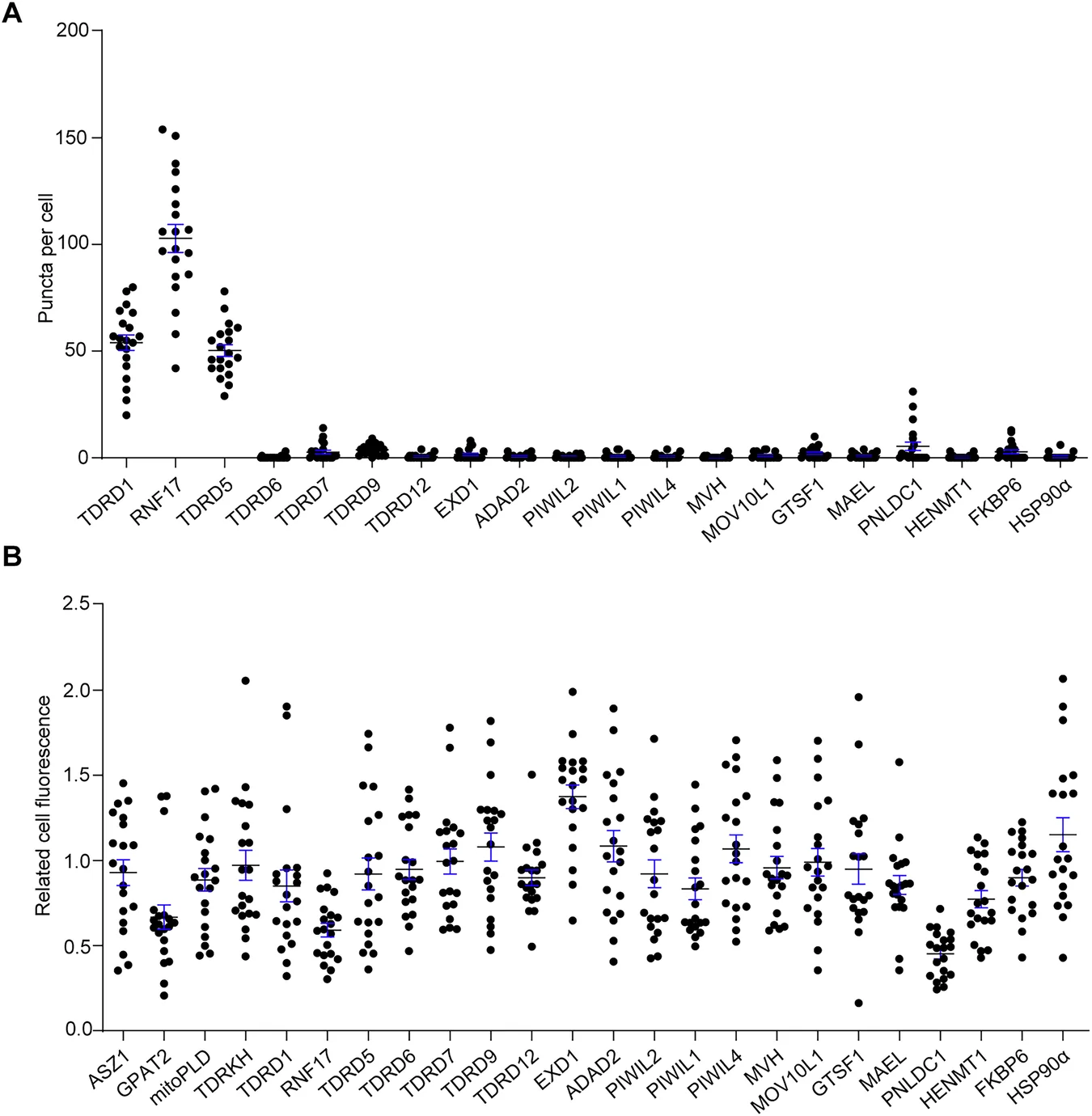
Quantification of 24 overexpressed proteins in HeLa cells. (**A**) Quantification of the puncta number per cell were shown in transfected HeLa cells. *n* = 20 cells. The data represent the mean ± s.e.m. (**B**) Quantification of the relative expression levels for the 24 overexpressed proteins in HeLa cells, measured as Corrected Total Cell Fluorescence (CTCF). *n* = 20 cells. The data represent the mean ± s.e.m.

**Figure EV2:**
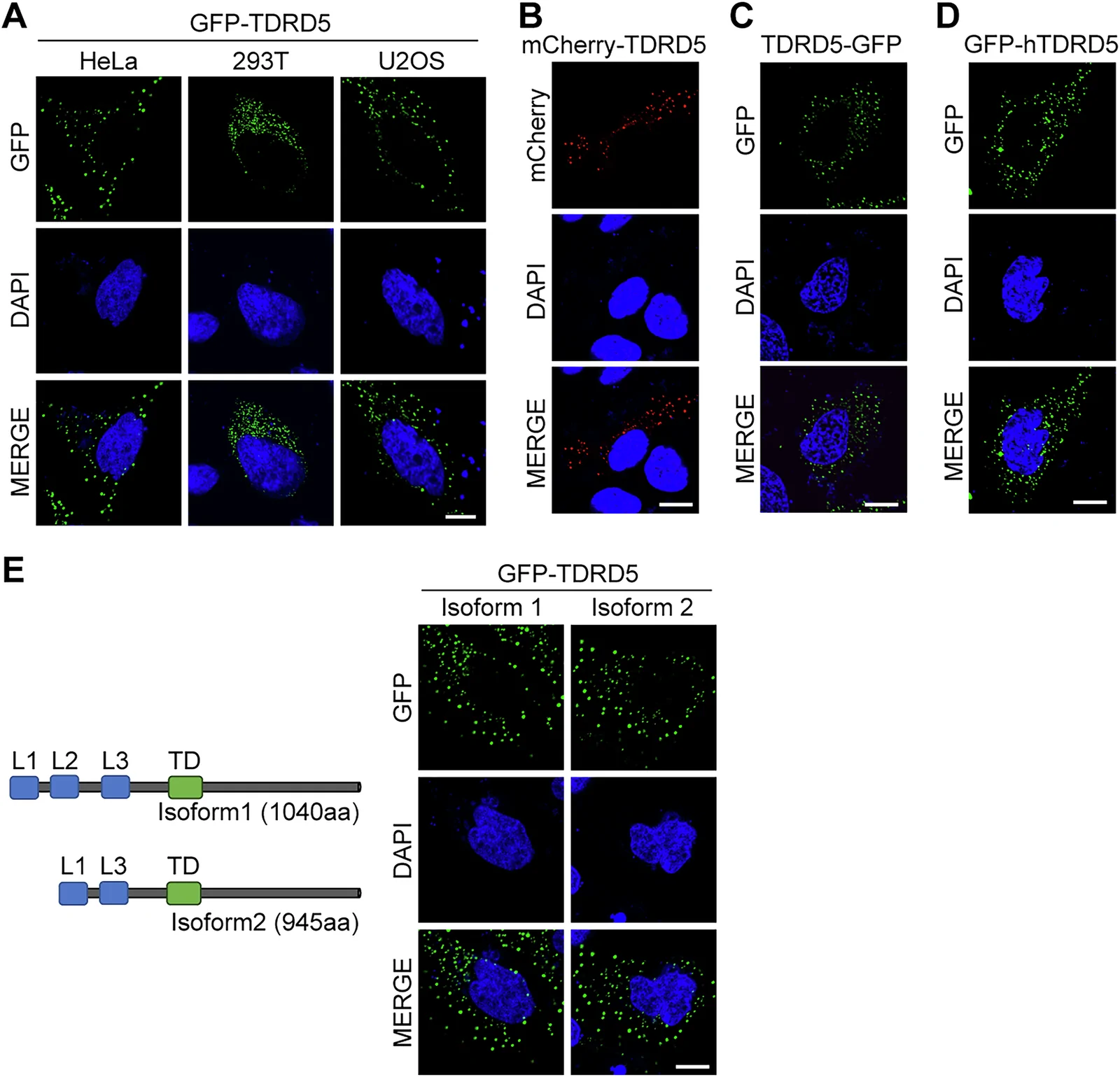
TDRD5 assembles fluid-like macromolecular condensates in cells. (**A**) Images of HeLa, 293T and U2OS cells transfected with GFP-TDRD5. Scale bars, 10 μm. (**B**) Images of HeLa cells transfected with mCherry-TDRD5. Scale bars, 10 μm. (**C**) Images of HeLa cells transfected with TDRD5-GFP. Scale bars, 10 μm. (**D**) Images of HeLa cells transfected with GFP tagged human TDRD5. Scale bars, 10 μm. (**E**) Images of HeLa cells transfected with GFP-TDRD5 Isoform 1 and 2. Scale bars, 10 μm.

To assess the phase separation potential of piRNA-related proteins, we constructed 24 GFP-tagged piRNA-related proteins and expressed them in HeLa cells. These proteins were chosen because they represent the core, well-established components currently known to be most essentially associated with piRNA biogenesis (Ku and Lin, 2014; Wang et al, 2022). We transfected them into HeLa cell separately and revealed that only three proteins—TDRD1, TDRD5, and RNF17/TDRD4—formed substantial cytoplasmic condensates in HeLa cells (Figs. 1A and EV1). TDRD1 and TDRD5 localize to the IMCs in gonocytes and pachytene spermatocytes, and their knockout disrupts IMC assembly and piRNA production (Chuma et al, 2006; Yabuta et al, 2011). RNF17 is mainly located in P-bodies. Although loss of RNF17 has been reported to cause aberrant activation of the secondary piRNA pathway, RNF17 does not substantially affect total piRNA production (Wasik et al, 2015; Xiong et al, 2023). Given that TDRD1 promotes IMC formation via phase separation, we asked whether TDRD5 also undergoes phase separation to facilitate IMC assembly and piRNA biogenesis. GFP-tagged TDRD5 consistently formed cytoplasmic droplets in HeLa, 293 T, and U2OS cells (Fig. EV2A). Similarly, mCherry-tagged TDRD5, C-terminal GFP-tagged TDRD5, and GFP-tagged human TDRD5 all formed condensates in HeLa cells (Fig. EV2B–D). Since TDRD5 has two isoforms in male germ cells, we tested both and found that each formed cytoplasmic condensates to a comparable extent (Fig. EV2E). Live-cell imaging further revealed highly dynamic fusion and fission events among TDRD5 condensates (Fig. 1B,C). Fluorescence recovery after photobleaching (FRAP) assays showed rapid recovery within GFP-TDRD5 condensates, with substantial replenishment occurring within ~140 s (Fig. 1D). These results indicate that TDRD5 forms dynamic, liquid-like condensates in cells.

### Tandem Lotus domains and an intrinsically disordered region drive TDRD5 condensation

**Figure 2:**
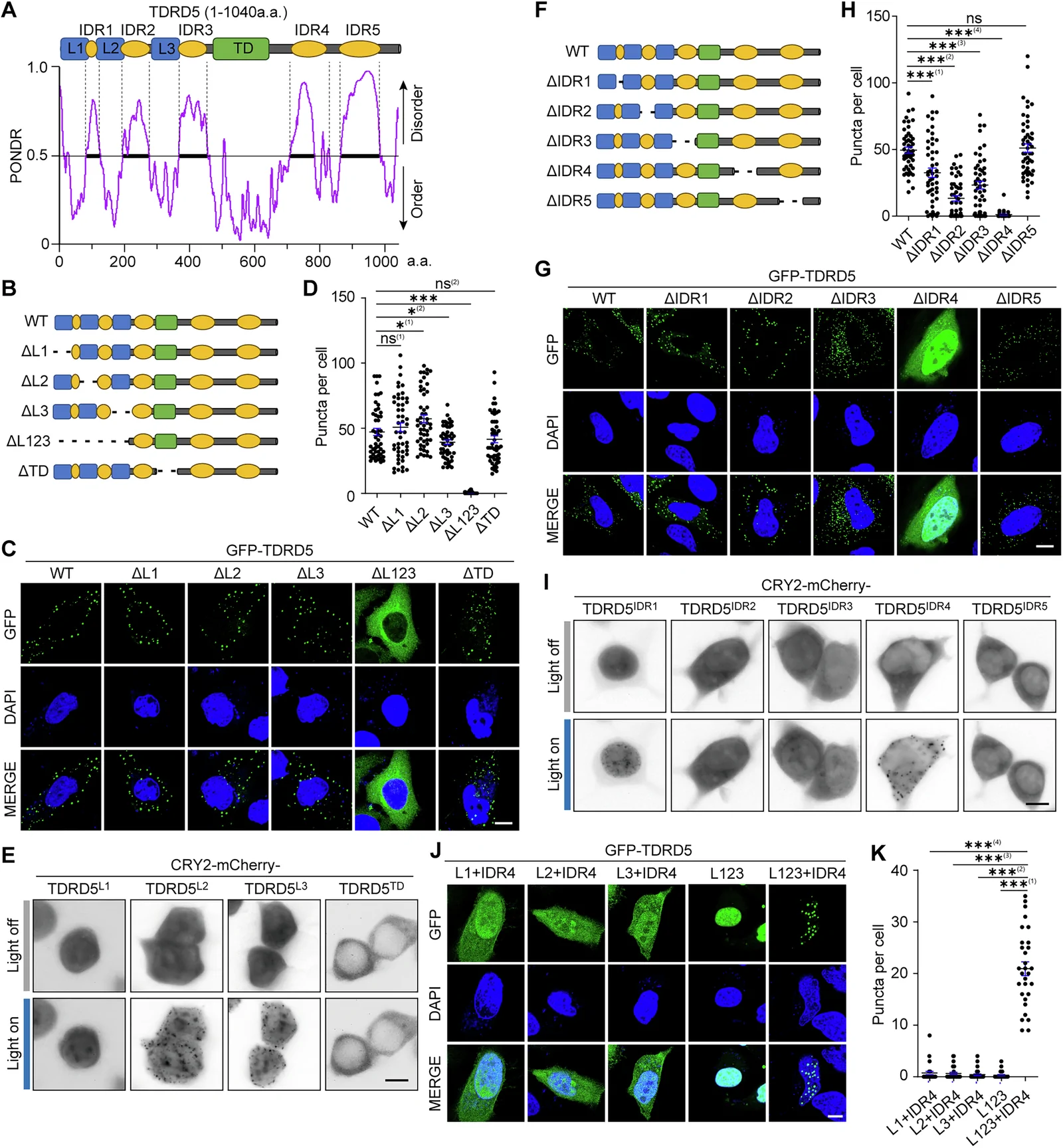
Lotus domains and IDR4 drive TDRD5 condensation. (**A**) Intrinsic disorder prediction and domain mapping of TDRD5: PONDR VSL2 disorder profile (bottom) and annotated domain schematic (top). (**B**) Schematic representation of the domain deletion constructs used to validate their impact on TDRD5. (**C**) Images of HeLa cells transfected with indicated constructs were shown. Scale bars, 10 μm. (**D**) Quantification of the puncta number per cell for each construct were shown based on (**C**). *n* = 50 cells. ns, not significant, *P* = 0.4154 (1), *P* = 0.1478 (2); *P = 0.0165 (1), 0.0190 (2); ***P = 6.56116E-22. *P* values were calculated using Welch’s *t* test. The data represent the mean ± s.e.m. (**E**) Images of 293 T cells transfected with indicated constructs before and after blue light activation (160 s). Scale bars, 10 μm. (**F**) Schematic representation of intrinsically disordered region deletion constructs used to validate their impact on TDRD5. (**G**) Images of HeLa cells transfected with indicated constructs were shown. Scale bars, 10 μm. (**H**) Quantification of the puncta number per cell for each construct were shown based on (**G**). *n* = 50 cells. ns, not significant, *P* = 0.6920; ***P = 0.0001 (1), 9.28388E-21(2), 3.26712E-10 (3), 3.67754E-27 (4). *P* values were calculated using Welch’s *t* test. The data represent the mean ± s.e.m. (**I**) Images of 293T cells transfected with indicated constructs before and after blue light activation (160 s). Scale bars, 10 μm. (**J**) Images of HeLa cells transfected with indicated constructs were shown. Scale bars, 10 μm. (**K**) Quantification of the puncta number per cell for each construct were shown based on (**J**). *n* = 30 cells. ***P = 5.71324E-18 (1), 6.06872E-18 (2), 7.13345E-18 (3), 7.2025E-18 (4). *P* values were calculated using Welch’s *t* test. The data represent the mean ± s.e.m. Source data are available online for this figure.

**Figure EV3:**
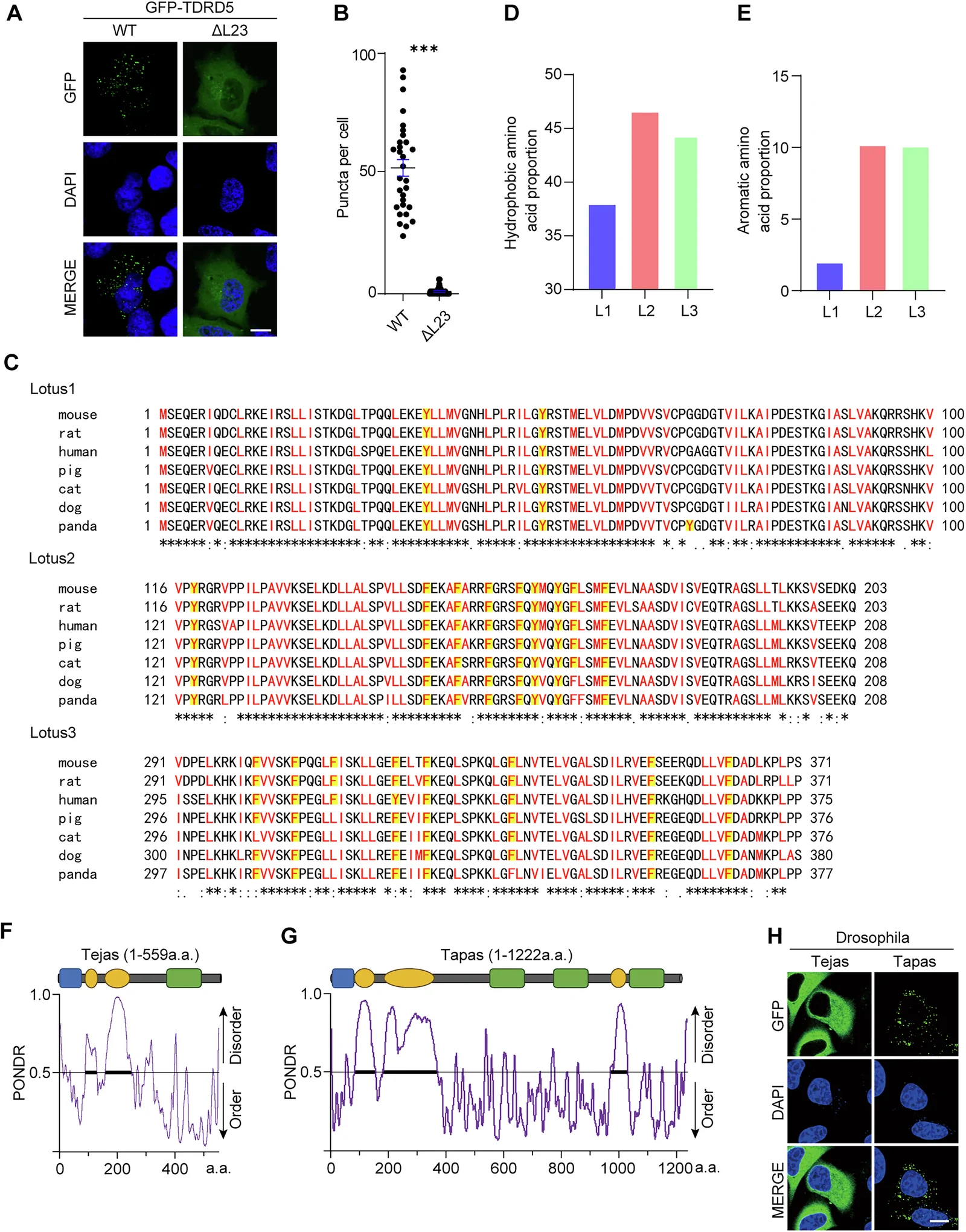
Lotus domains and IDR4 drive TDRD5 condensation. (**A**) Images of HeLa cells transfected with indicated plasmids. Scale bars, 10 μm. (**B**) Quantification of the puncta number per cell were shown in transfected HeLa cells. *n* = 30 cells. The data represent the mean ± s.e.m. ***P = 3.48374E-15. *P* values were calculated using Welch’s *t* test. (**C**) Multiple sequence alignment of Lotus domains of TDRD5 from mouse, human, rat, panda, cat, pig and dog using Clustal Omega. The hydrophobic amino acids were shown in red. The aromatic amino acids were highlighted in yellow. (**D**) Proportion of hydrophobic amino acids in three Lotus domains of TDRD5. (**E**) Proportion of Aromatic amino acids in three Lotus domains of TDRD5. (**F**, **G**) Intrinsic disorder prediction and domain mapping of Tejas (**A**) and Tapas (**B**): PONDR VSL2 disorder profile (bottom) and annotated domain schematic (top). (**H**) Images of HeLa cells transfected with Tejas and Tapas were shown. Scale bars, 10 μm.

TDRD5 contains three Lotus domains, which recognize G-rich RNAs (Ding et al, 2018), and one Tudor domain (Fig. 2A). Since RNA-binding and Tudor domains have been implicated in condensate formation (Courchaine et al, 2021; Shao et al, 2022), we systematically deleted each domain to assess their contributions (Fig. 2B). Individual deletion of any Lotus or Tudor domain did not prevent condensation; however, simultaneous deletion of all three Lotus domains completely abolished condensate formation (Fig. 2C,D). Using an optoDroplet assay (Zhang et al, 2019), we fused each TDRD5 domain to the light-responsive CRY2 domain and mCherry. Upon blue-light induction in 293 T cells, the second and third Lotus (L2 and L3), but not the first Lotus (L1) or Tudor, formed condensates (Fig. 2E). Consistently, deletion of L2 and L3 was sufficient to abolish TDRD5 condensation to the same extent as the deletion of all three Lotus domains (Fig. EV3A,B). Multi-species alignment revealed that all three Lotus domains (L1-L3) are highly conserved across mammals (Fig. EV3C). However, L2 and L3 contain a notably higher proportion of hydrophobic and aromatic residues compared to L1 (Fig. EV3D,E). Given that hydrophobic and aromatic residues are key drivers of multivalent interactions in biomolecular condensates, their relative enrichment in L2 and L3 likely underpins the intrinsic condensation capacity.

Because intrinsically disordered regions (IDRs) often promote biomolecular condensation (Shin et al, 2017), we analyzed mouse TDRD5 using PONDR and identified five putative IDRs (Fig. 2A). Deletion of IDR1, IDR2, or IDR3 mildly impaired TDRD5 droplet formation, whereas loss of IDR4 completely disrupted it. (Fig. 2F–H). The optoDroplet assay confirmed that IDR4 has a strong capacity to form light-induced condensates (Fig. 2I). We next tested whether Lotus domains cooperate with IDR4 by generating fusion proteins. Only constructs containing all three Lotus domains together with IDR4 formed pronounced condensates (Fig. 2J,K). Thus, the N-terminal tandem Lotus domains and IDR4 collaboratively promote TDRD5 condensation. This is consistent with previous findings that the *Drosophila* TDRD5 homolog Tejas relies on its IDR for granule assembly, supporting a conserved role of IDRs in TDRD5 function (Lin et al, 2023).

We then asked whether this condensation ability is conserved in *Drosophila*. While both mouse TDRD5 and TDRD7 contain Lotus and Tudor domains, only TDRD5 forms condensates (Fig. 1A). Similarly, in *Drosophila*, Tejas and Tapas contain these domains and function in piRNA biogenesis and transposon silencing. PONDR predicted IDRs in both proteins (Fig. EV3F,G), but only Tapas formed condensates when expressed in HeLa cells (Fig. EV3H), indicating conserved yet divergent condensation behavior among Lotus and IDR containing proteins.

### TDRD5 undergoes RNA-dependent phase separation

**Figure EV4:**
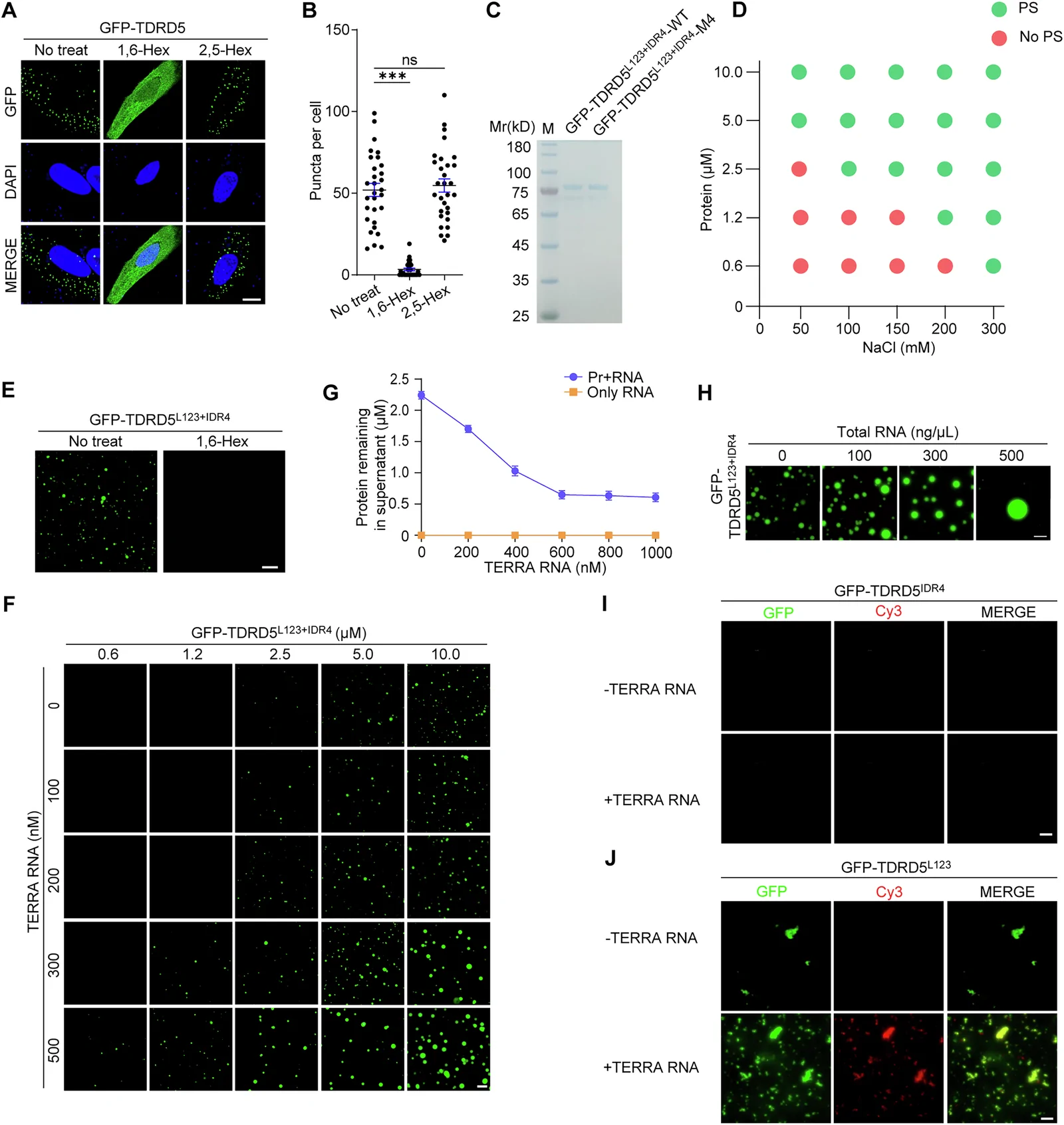
TDRD5 undergoes RNA-dependent phase separation. (**A**) Images of HeLa cells transfected with GFP-TDRD5 exposed to 1,6-hexanediol (1,6-Hex) or 2,5-hexanediol (2,5-Hex) were shown. Scale bars, 10 μm. (**B**) Quantification of the puncta number per cell were shown based on (**A**). *n* = 30 cells. ns, not significant, *P* = 0.6341; ***P = 4.73229E-13. *P* values were calculated using Welch’s *t* test. The data represent the mean ± s.e.m. (**C**) SDS-PAGE analysis showing the purified proteins. The protein standard molecular weights are labeled. (**D**) Phase diagram of purified recombinant GFP-TDRD5^L123+IDR4^ at various amounts and with increasing concentrations of NaCl. (**E**) Purified protein of GFP-TDRD5^L123+IDR4^ exposed to 1,6-hexanediol were shown (5 μM Protein, 150 mM NaCl). Scale bars, 10 μm. (**F**) Phase separation of purified recombinant GFP-TDRD5^L123+IDR4^ at various amounts and with increasing concentrations of TERRA RNA (150 mM NaCl). Scale bar, 10  μm. (**G**) Protein remaining in supernatant after phase separation for 2.5 μM GFP-TDRD5^L123+IDR4^ for 0–1000 nM TERRA RNA with 150 mM NaCl. *n* = 3. (**H**) In vitro droplet formation of purified GFP-TDRD5^L123 + IDR4^ (5 μM Protein, 150 mM NaCl) at the indicated total RNA concentrations in the presence of 150 mM NaCl. Scale bars, 10 μm. (**I**) Phase separation of purified recombinant GFP-TDRD5^IDR4^ with Cy3 labeled TERRA RNA (10 μM Protein, 150 mM NaCl). Scale bars, 10 μm. (**J**) Phase separation of purified recombinant GFP-TDRD5^L123^ with Cy3 labeled TERRA RNA (10 μM Protein, 150 mM NaCl). Scale bars, 10 μm.

**Figure 3:**
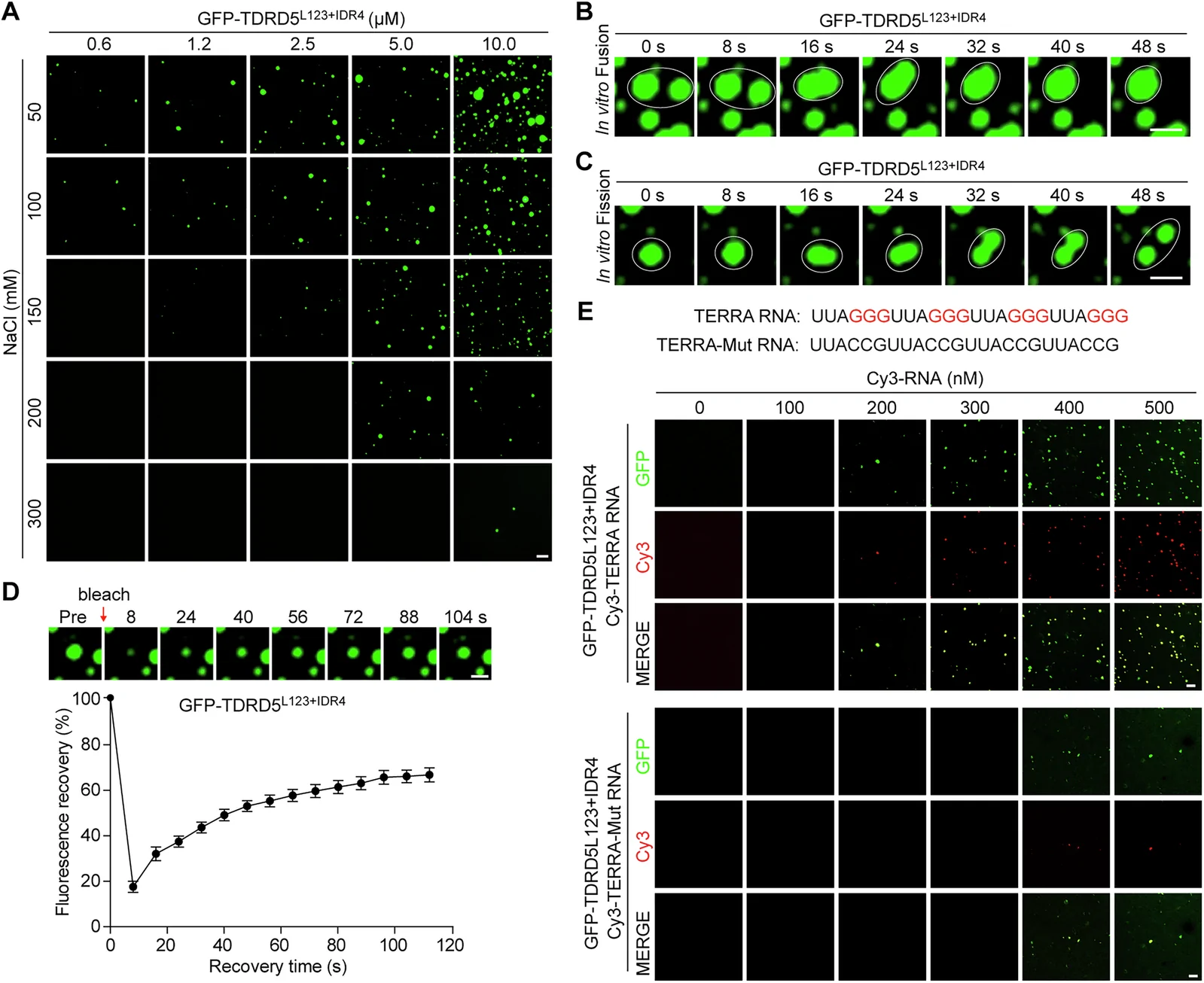
TDRD5 undergoes RNA-dependent phase separation. (**A**) Phase separation of purified recombinant GFP-TDRD5^L123+IDR4^ at various amounts and with increasing concentrations of NaCl. Scale bar, 10 μm. (**B**, **C**) The fusion (**B**) and fission (**C**) of purified recombinant GFP-TDRD5^L123+IDR4^ droplets in vitro (5 μM Protein, 150 mM NaCl). Scale bar, 5 μm. (**D**) FRAP analysis of purified recombinant GFP-TDRD5^L123+IDR4^ droplets in vitro. Quantification of FRAP data is shown below. *n* = 10 droplets. The data represent the mean ± s.e.m. Scale bars, 5 μm. (**E**) Phase separation of purified recombinant GFP-TDRD5^L123+IDR4^ with increasing concentrations of Cy3 labeled TERRA RNA (upper panels) or TERRA-Mut RNA (lower panels) in vitro (1 μM Protein, 150 mM NaCl). Scale bars, 10 μm. Source data are available online for this figure.

To confirm that TDRD5 condensation results from phase separation, we treated GFP-TDRD5 expressing HeLa cells with 1,6-hexanediol, which disrupts weak hydrophobic interactions in condensates. This treatment significantly reduced TDRD5 granules, whereas the control reagent 2,5-hexanediol did not (Fig. EV4A,B). We next turned to in vitro reconstitution. Although full-length TDRD5 could not be purified, we successfully purified a GFP-tagged fragment containing the three Lotus domains and IDR4 (TDRD5^L123+IDR4^; Fig. EV4C). This recombinant protein underwent concentration-dependent phase separation in vitro, which was inhibited by increasing salt concentrations (Figs. 3A and EV4D). The TDRD5^L123+IDR4^ droplets were dissolved by 1,6-hexanediol (Fig. EV4E), underwent rapid fusion and fission (Fig. 3B,C), and showed rapid fluorescence recovery in FRAP assays (Fig. 3D), confirming their liquid-like behavior.

Since Lotus domains bind RNA G-quadruplex (G4) or G-rich RNAs (Ding et al, 2020), we tested whether RNA binding influences phase separation. Adding Cy3-labeled TERRA RNA, which forms G4 structures, strongly enhanced TDRD5^L123+IDR4^ droplet formation in vitro (Figs. 3E and EV4F). A mutant TERRA RNA, unable to form G4, had a milder effect (Fig. 3E). To quantitatively assess the effect of TERRA RNA on TDRD5 phase separation, we performed a centrifugation-based supernatant depletion assay. The addition of TERRA RNA induced a dose-dependent decrease in the soluble fraction of TDRD5^L123+IDR4^ (Fig. EV4G). Total RNA from adult testes also promoted droplet formation (Fig. EV4H). Together, these data indicate RNA binding facilitates TDRD5 phase separation.

To further dissect the minimal requirements for TDRD5 phase separation in vitro, we purified the recombinant individual domains, TDRD5^L123^ and TDRD5^IDR4^, and tested whether each alone is sufficient to drive phase separation. TDRD5^IDR4^ failed to form liquid droplets regardless of the presence or absence of TERRA RNA. TDRD5^L123^ alone did not form liquid condensates but instead produced amorphous aggregates. Notably, the addition of G-rich RNA exacerbated TDRD5^L123^ precipitation rather than inducing liquid-liquid phase separation (Fig. EV4I,J). Together, these results support a synergistic mechanism: proper liquid-like phase separation of TDRD5 requires both the RNA-binding capacity of the Lotus domains and the dynamic flexibility conferred by IDR4.

### Aromatic residues in IDR4 are essential for TDRD5 phase separation

**Figure 4:**
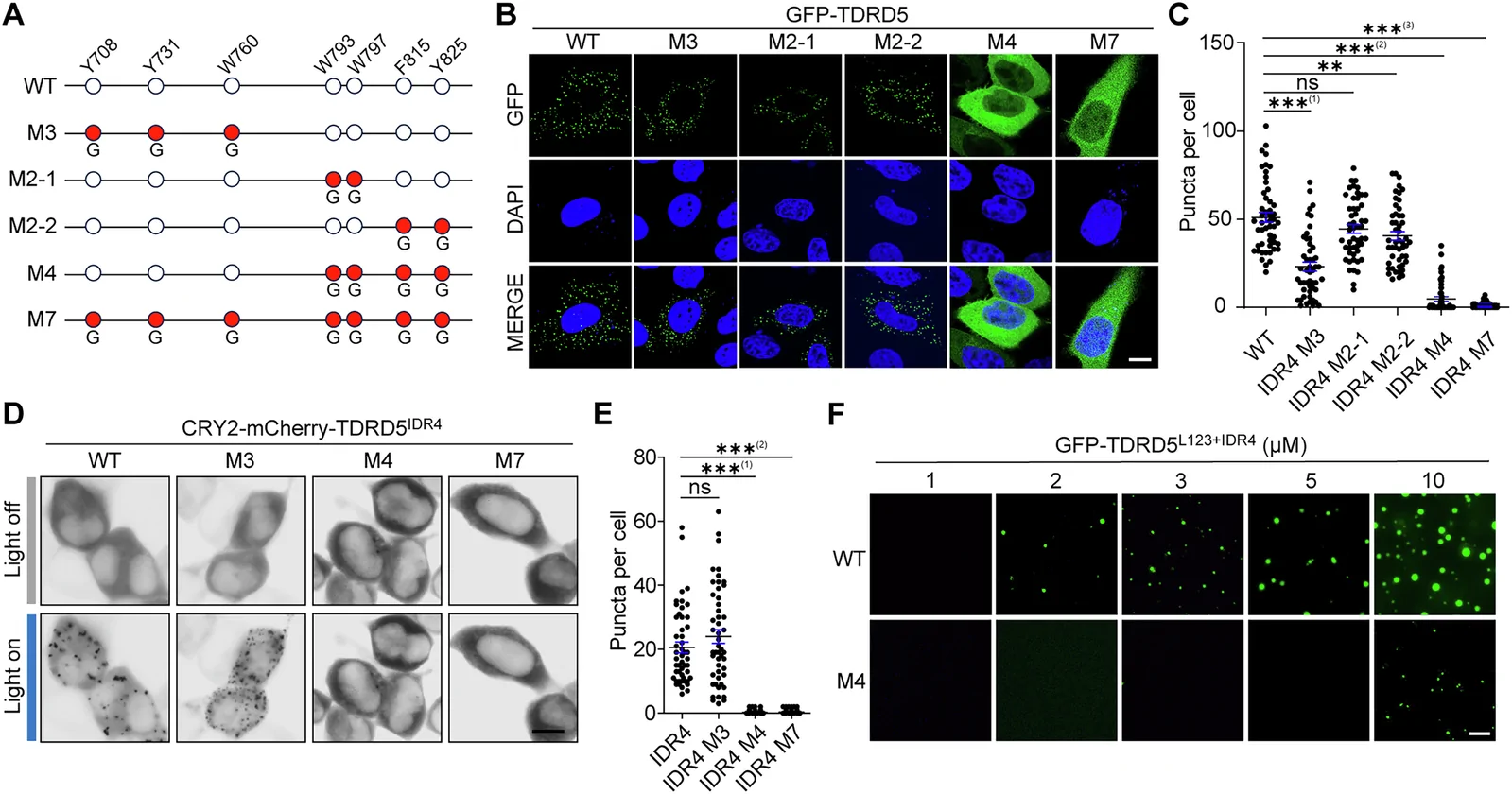
Aromatic residues in IDR4 are essential for TDRD5 phase separation. (**A**) Schematic representation of the mutation of aromatic amino acids in TDRD5. Aromatic amino acids are shown in white and mutations (Glycine) are shown in red. (**B**) Images of HeLa cells transfected with indicated constructs were shown. Scale bars, 10 μm. (**C**) Quantification of the puncta number per cell for each construct were shown based on (**B**). *n* = 50 cells. ns, not significant, *P* = 0.0797; **P = 0.0060; ***P = 6.38799E-11 (1), 2.8603E-23 (2), 4.15282E-23 (3). *P* values were calculated using Welch’s *t* test. The data represent the mean ± s.e.m. (**D**) Images of 293T cells transfected with indicated constructs before and after blue light activation (160 s). Scale bars, 10 μm. (**E**) Quantification of the puncta number per cell for each construct were shown based on (**D**). *n* = 50 cells. ns, not significant, *P* = 0.2140; ***P = 2.50027E-16 (1), 2.86415E-16 (2). *P* values were calculated using Welch’s *t* test. The data represent the mean ± s.e.m. (**F**) Phase separation of purified recombinant GFP-TDRD5^L123+IDR4^ and GFP-TDRD5^L123+IDR4^-M4 with 150 mM NaCl. Scale bar, 10 μm. Source data are available online for this figure.

**Figure EV5:**
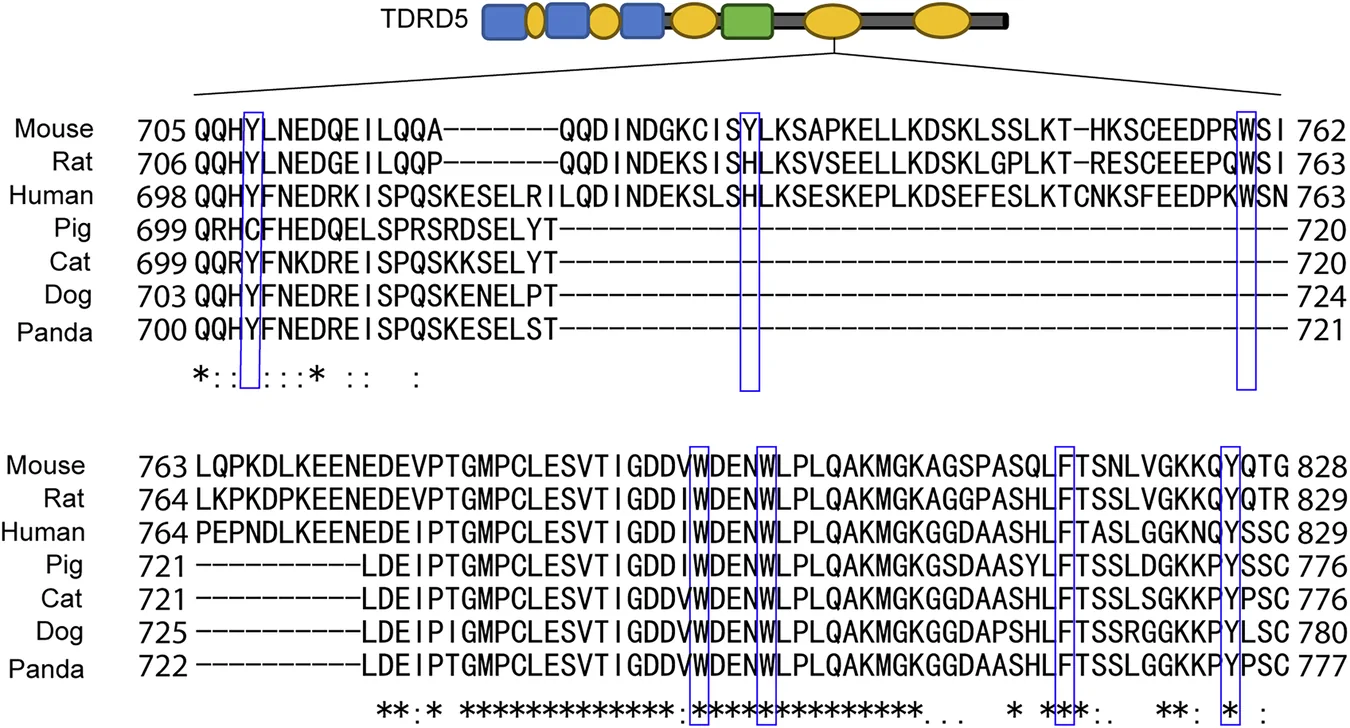
Aromatic residues in IDR4 are conserved in mammals. Multiple sequence alignment of TDRD5 IDR4 from mouse, human, rat, panda, cat, pig and dog using Clustal Omega. The aromatic amino acids were shown in blue square.

Aromatic residues in IDRs often mediate π-π or π-cation interactions that drive phase separation (Martin et al, 2020; Poh and Mueller-Cajar, 2024; Rekhi et al, 2024; Wang et al, 2018). IDR4 contains seven aromatic residues (Y, W, F; Fig. 4A). Mutating the first three (M3) slightly impaired condensation, while mutating the last four (M4) or all seven (M7) strongly disrupted it (Fig. 4B,C). Mutating two of the last four residues (M2-1, M2-2) did not affect condensation, suggesting that the collective presence of these four residues is essential. Consistent with their functional importance, the last four aromatic residues are highly conserved in mammals (Fig. EV5). The optoDroplet assay confirmed the critical role of these residues (Fig. 4D,E). Purified TDRD5^L123+IDR4^ M4 protein exhibited markedly reduced phase separation and only formed condensates at high concentrations (10 μM), indicating impaired phase separation capacity. (Fig. 4F). We therefore conclude that IDR4 triggers TDRD5 phase separation through aromatic residues.

### TDRD5 phase separation deficiency impairs male fertility in mice

**Figure EV6:**
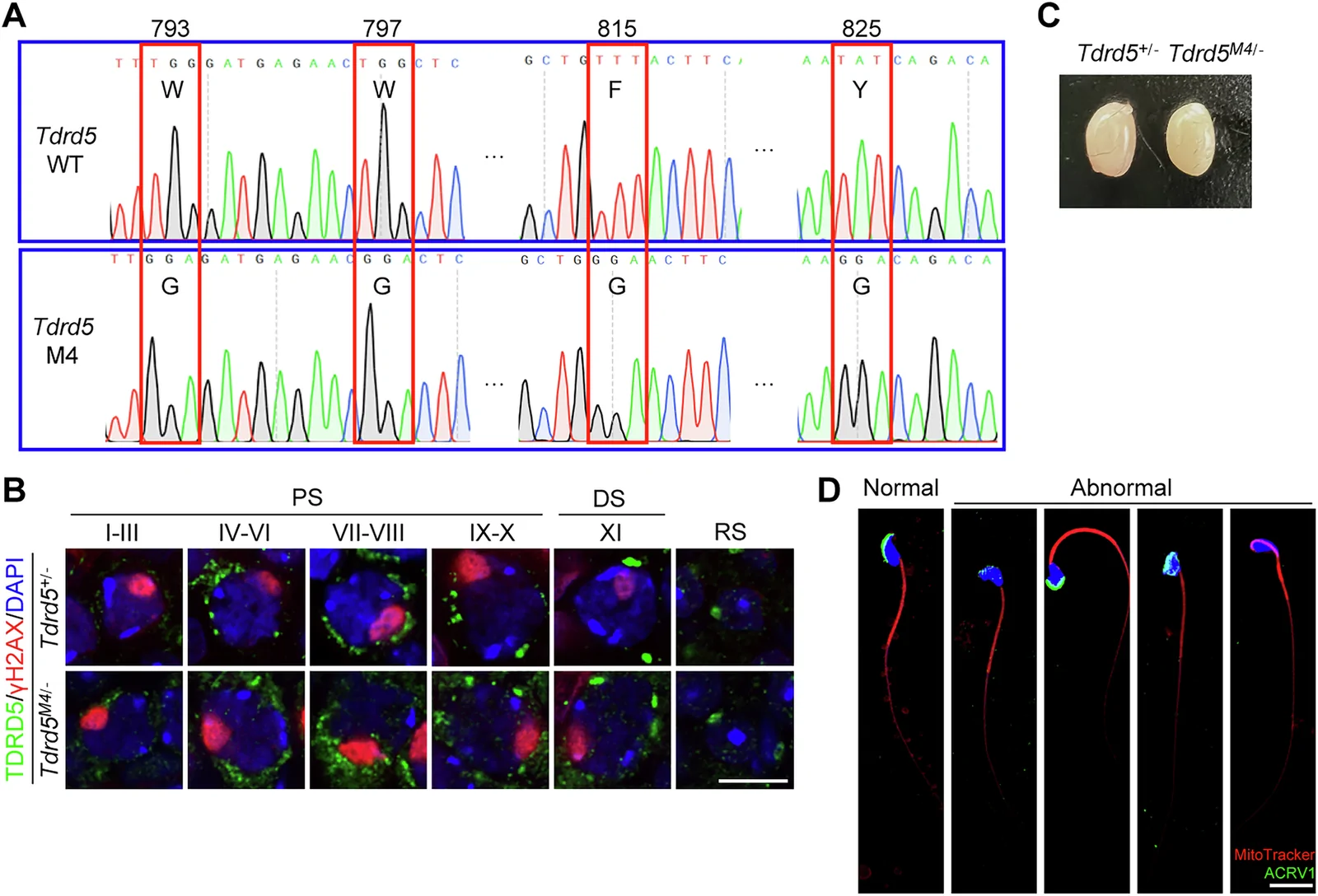
TDRD5 phase separation deficiency impairs male fertility in mice. (**A**) Sanger sequencing of genotyping PCR products amplified to confirm the *Tdrd5* M4 mutant alleles. (**B**) Co-immunostaining of TDRD5 and γH2AX on testis sections from adult *Tdrd5*^*+/−*^ and *Tdrd5*^*M4/−*^ mice. Different germ cell types were distinguished according to γH2AX and DAPI staining. PS, pachytene spermatocyte; DS, diplotene spermatocyte; RS, round spermatid. Scale bars, 10 μm. (**C**) Testis size of adult *Tdrd5*^*+/−*^ and *Tdrd5*^*M4/−*^ mice are shown. (**D**) Co-immunostaining of ACRV1 and MitoTracker on sperms from adult *Tdrd5*^*M4/−*^ mouse epididymis. Scale bars, 10 μm.

**Figure 5:**
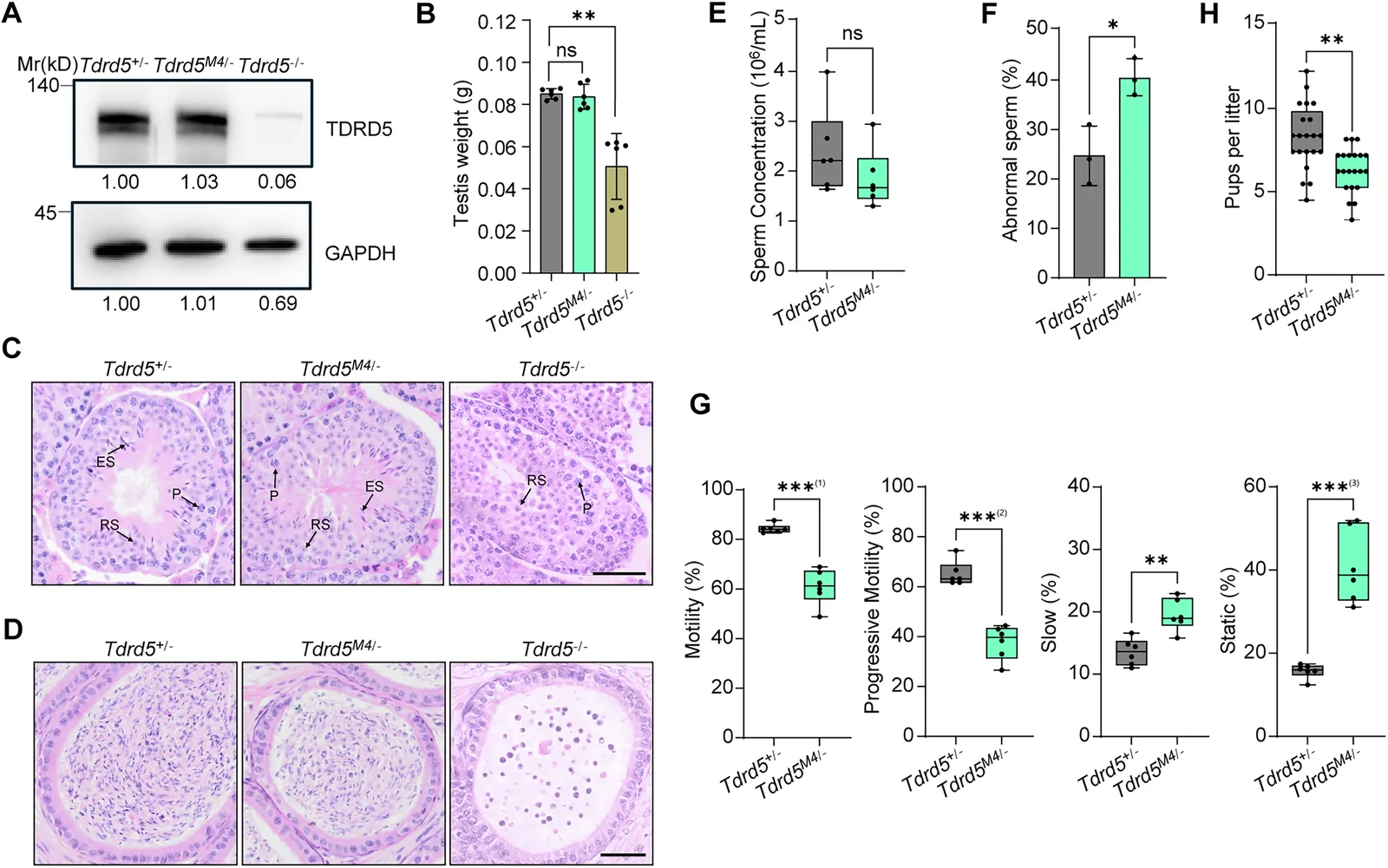
TDRD5 phase separation deficiency impairs male fertility in mice. (**A**) Western blotting of TDRD5 expression in adult *Tdrd5*^*+/−*^, *Tdrd5*^*M4/−*^and *Tdrd5*^*−/−*^ testes. GAPDH served as a loading control. (**B**) Testis weights of adult *Tdrd5*^*+/−*^, *Tdrd5*^*M4/−*^and *Tdrd5*^*−/−*^ mice are shown. *n*  = 6 (biological replicates). ns, not significant, *P* = 0.6117; **P = 0.0026. *P* values were calculated using Welch’s *t* test. The data represent the mean ± s.d. (**C**) H&E staining of testis sections from adult *Tdrd5*^*+/−*^, *Tdrd5*^*M4/−*^and *Tdrd5*^*−/−*^ mice are shown. Scale bars, 50 μm. P pachytene spermatocytes, RS round spermatids, ES elongating spermatids. (**D**) H&E staining of epididymis sections from adult *Tdrd5*^*+/−*^, *Tdrd5*^*M4/−*^and *Tdrd5*^*−/−*^ mice are shown. Scale bars, 50 μm. (**E**) Sperm concentration of adult *Tdrd5*^*M4/−*^and *Tdrd5*^*+/−*^ mice are shown. *n*  = 6 (biological replicates). *Tdrd5*^*+/−*^ group: Minimum = 1.639, Maximum = 3.990, Median = 2.210, 25% Percentile = 1.707, 75% Percentile = 2.989. *Tdrd5*^*M4/−*^ group: Minimum = 1.300, Maximum = 2.939, Median = 1.667, 25% Percentile = 1.452, 75% Percentile = 2.452. ns, not significant, *P* = 0.2233. *P* values were calculated using Welch’s *t* test. (**F**) The percentage of abnormal sperm in adult *Tdrd5*^*M4/−*^and *Tdrd5*^*+/−*^ mice are shown. *n*  = 3 (biological replicates). *P = 0.0265. *P* values were calculated using Welch’s *t* test. The data represent the mean ± s.d. (**G**) Sperm motility of adult *Tdrd5*^*M4/−*^and *Tdrd5*^*+/−*^ mice are shown. *n*  = 6 (biological replicates). *Tdrd5*^*+/−*^ group: Minimum = 82.60 (Motility), 61.70 (Progressive Motility), 11.10 (Slow), 12.40 (Static); Maximum = 87.60 (Motility), 74.50 (Progressive Motility), 16.60 (Slow), 17.40 (Static); Median = 84.05(Motility), 63.15 (Progressive Motility), 13.60 (Slow), 15.95 (Static); 25% Percentile = 83.05(Motility), 61.70 (Progressive Motility), 11.45 (Slow), 14.80 (Static); 75% Percentile = 85.20 (Motility), 68.65 (Progressive Motility), 15.25 (Slow), 16.95 (Static). *Tdrd5*^*M4/−*^ group: Minimum = 48.80 (Motility), 26.50 (Progressive Motility), 15.58 (Slow), 31.10 (Static); Maximum =  68.90 (Motility), 44.40 (Progressive Motility), 22.90 (Slow), 51.90 (Static); Median = 61.20 (Motility), 39.65 (Progressive Motility), 18.95 (Slow), 38.80 (Static); 25% Percentile = 56.00 (Motility), 31.38 (Progressive Motility), 17.83 (Slow), 32.75 (Static); 75% Percentile = 67.25 (Motility), 43.35 (Progressive Motility), 22.15 (Slow), 51.38 (Static). **P = 0.0013; ***P = 0.0003 (1), 1.18028E-05 (2), 0.0007 (3). *P* values were calculated using Welch’s *t* test. (**H**) Fertility analysis for adult *Tdrd5*^*M4/−*^and *Tdrd5*^*+/−*^ mice are shown. *n*  = 21 litters from 4 breeding cages (biological replicates). *Tdrd5*^*+/−*^ group: Minimum = 4.0, Maximum = 12.0, Median = 8.0, 25% Percentile = 7.0, 75% Percentile = 9.5. *Tdrd5*^*M4/−*^ group: Minimum = 3.0, Maximum = 8.0, Median = 6.0, 25% Percentile = 5.0, 75% Percentile = 7.0. **P = 0.0017. *P* values were calculated using Welch’s *t* test. Source data are available online for this figure.

To assess the physiological role of TDRD5 phase separation, we used CRISPR-Cas9 to generate mice carrying the M4 mutation (*Tdrd5*^*M4*^) mice (Fig. EV6A). Western blot (WB) and immunostaining analyses consistently revealed that the *Tdrd5*^*M4*^ mutation does not affect TDRD5 protein stability (including isoform1 and isoform2) and localization in adult testes (Figs. 5A and EV6B). *Tdrd5*^*M4/−*^ adult mice exhibited testis weights and size comparable to these of control mice (Figs. 5B and EV6C). Histological examination by H&E staining indicated that spermatogenesis proceeds normally in *Tdrd5*^*M4/−*^ mutants, with all developmental stages of germ cells being readily observable (Fig. 5C). Sperm counts showed no significant difference in the epididymides of *Tdrd5*^*M4/−*^ mice (Fig. 5D,E). However, morphological analysis revealed that *Tdrd5*^*M4/−*^ mice exhibited more abnormal sperm (Figs. 5F and EV6D), including defects in head shape, acrosome abnormalities and tail coiled. Consistent with this, the sperm motility was significantly impaired in *Tdrd5*^*M4/−*^ mice (Fig. 5G). As a result, *Tdrd5*^*M4/−*^ mice showed reduced male fertility (Fig. 5H). The data indicate that TDRD5 phase separation play roles in male fertility in mice.

### TDRD5 phase separation is required for IMC assembly and fetal piRNA biogenesis in gonocytes

**Figure 6:**
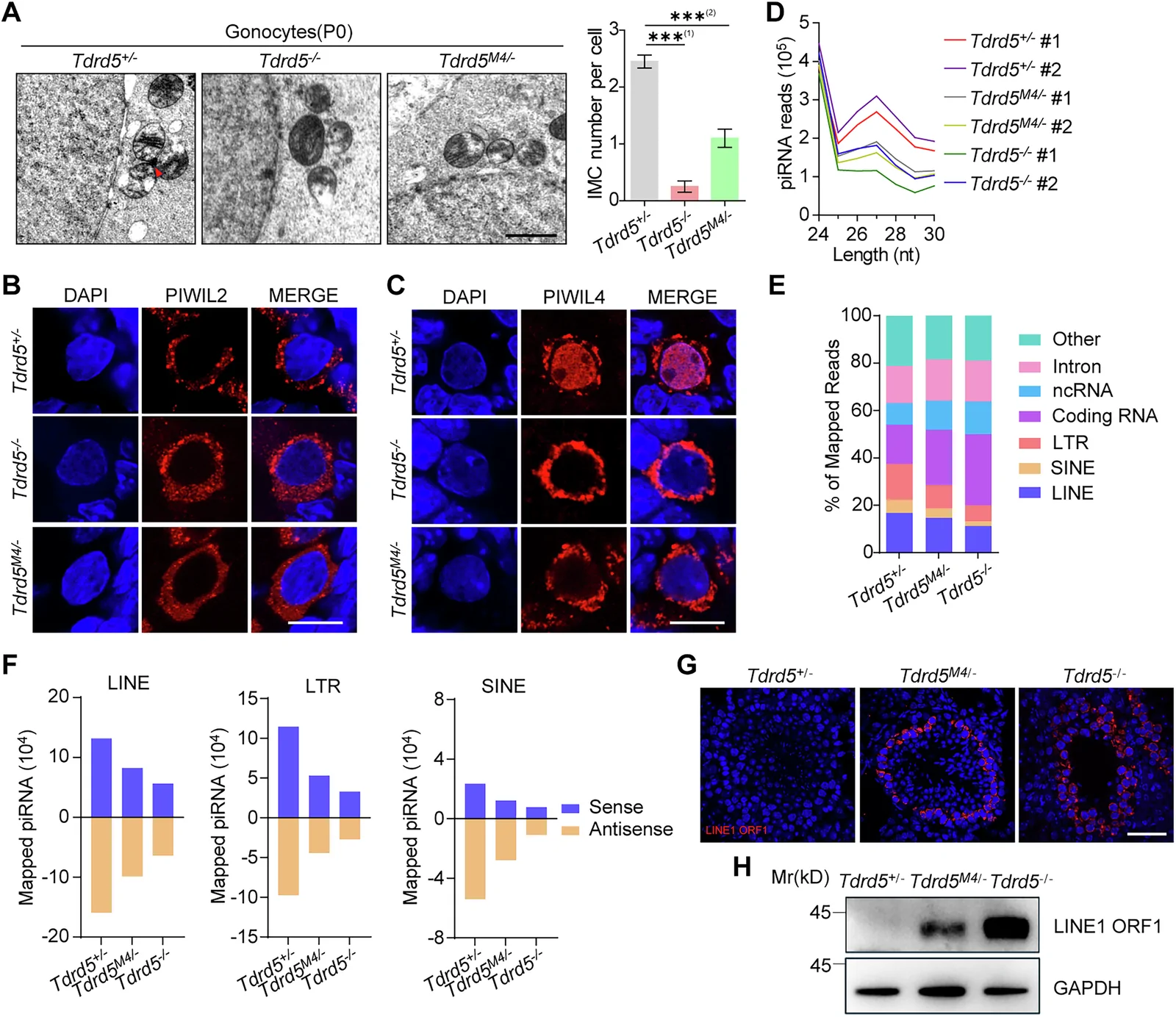
TDRD5 phase separation is required for IMC assembly and fetal piRNA biogenesis in gonocytes. (**A**) TEM on gonocytes from P0 testes. Scale bars, 1 μm. Quantification of the IMC numbers per cell is shown on the right. *n*  = 20 cells. ***P = 6.04878E-17 (1), 6.59092E-08 (2). *P* values were calculated using Welch’s *t* test. The data represent the mean ± s.e.m. The IMC structure is indicated by red arrow. (**B**) Immunostaining of PIWIL2 on P0 gonocytes. Scale bars, 10  μm. (**C**) Immunostaining of PIWIL4 on P0 gonocytes. Scale bars, 10 μm. (**D**) The length distribution of piRNAs in P0 testes. Data were normalized by miRNA reads. (**E**) Genomic annotation of piRNAs in P0 testes. (**F**) Numbers of piRNAs mapped to transposon LINE, LTR, and SINE. Data were normalized by miRNA reads. *n* = 2. (**G**) Immunostaining of LINE1 ORF1 on testis sections from adult mice. Scale bars, 50  μm. (**H**) Western blotting of LINE1 ORF1 for adult mice. GAPDH served as a control. Source data are available online for this figure.

We next asked whether *Tdrd5*^*M4*^ mutation impairs IMC formation and piRNA biogenesis in germ cells. We harvested testes from P0 mice for TEM examination. In *Tdrd5*^*+/−*^ mice, IMC were readily detected in gonocytes. However, the IMC structures were impaired in both *Tdrd5*^*M4/−*^and *Tdrd5*^*−/−*^ mice (Fig. 6A). Notably, the IMC defect was less severe in *Tdrd5*^*M4/−*^ than *Tdrd5*^*−/−*^ gonocytes (Fig. 6A), suggesting that the M4 mutation does not completely abolish TDRD5 phase separation, or that TDRD5 retains additional functions supporting IMC assembly independent of phase separation. We next assessed the impact on fetal piRNA biogenesis by analyzing the localization of PIWI proteins PIWIL2 and PIWIL4 in P0 gonocytes. PIWIL2 formed cytoplasmic granules in all genotypes (Fig. 6B), whereas PIWIL4 was mislocalized from the nucleus to the cytoplasm in *Tdrd5*^*M4/−*^and *Tdrd5*^*−/−*^ germ cells (Fig. 6C), indicating defective piRNA-mediated regulation. Small RNA sequencing of P0 testes revealed a significant reduction in piRNA (25-30 nt) abundance after normalization to miRNA levels (21–23 nt) in both *Tdrd5*^*M4/−*^ and *Tdrd5*^*−/−*^ mice (Fig. 6D). Genomic mapping of piRNA reads showed a decrease in repeat-associated piRNAs (Fig. 6E), with substantial reductions in piRNAs derived from LINE, LTR, and SINE elements (Fig. 6F). As a result, LINE1 expression was upregulated in *Tdrd5*^*M4/−*^ testes (Fig. 6G,H). Together, these results demonstrate that TDRD5 phase separation is critical for IMC assembly and fetal piRNA biogenesis in gonocytes.

### TDRD5 phase separation is dispensable for pachytene piRNA biogenesis

**Figure EV7:**
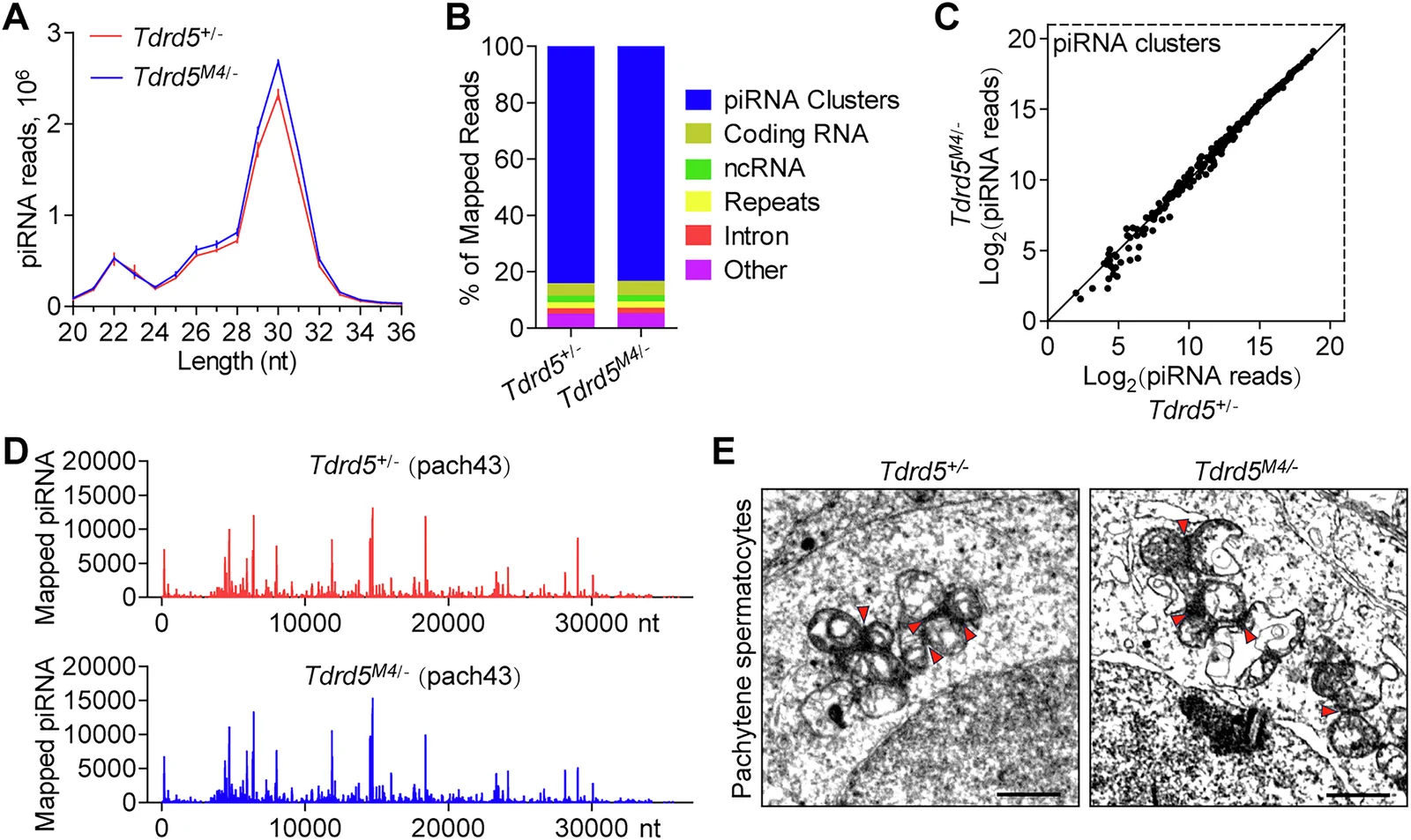
TDRD5 phase separation is dispensable for pachytene piRNA biogenesis. (**A**) The length distribution of piRNAs in adult testes. Data were normalized by miRNA reads. (**B**) Genomic annotation of total piRNA from adult testes. (**C**) Scatter plot of piRNA reads mapped to individual piRNA clusters from *Tdrd5*^*+/−*^ and *Tdrd5*^*M4/−*^ mice. (**D**) Distribution of piRNA reads mapping to a representative piRNA clusters (pach43) from *Tdrd5*^*+/−*^ and *Tdrd5*^*M4/−*^ mice. (**E**) TEM on pachytene spermatocytes from adult testes. Scale bars, 1 μm. The IMC structure is indicated by red arrow.

Although TDRD5 binds piRNA precursors and supports pachytene piRNA production, the role of its phase separation in this process remained unclear. We sequenced small RNAs from adult testes and found that pachytene piRNA abundance was not significantly affected in *Tdrd5*^*M4/−*^ mice (Fig. EV7A). The proportion of cluster-derived piRNAs also remained unchanged (Fig. EV7B), and processing of individual piRNA clusters appeared normal (Fig. EV7C,D). In line with these observations, IMC structures were largely intact in *Tdrd5*^*M4/−*^ adult testes (Fig. EV7E). These data indicate that TDRD5 phase separation is not essential for IMC formation or pachytene piRNA biogenesis in pachytene spermatocytes.

### TDRD5 is recruited to the IMC through interaction with PIWIL2

**Figure 7:**
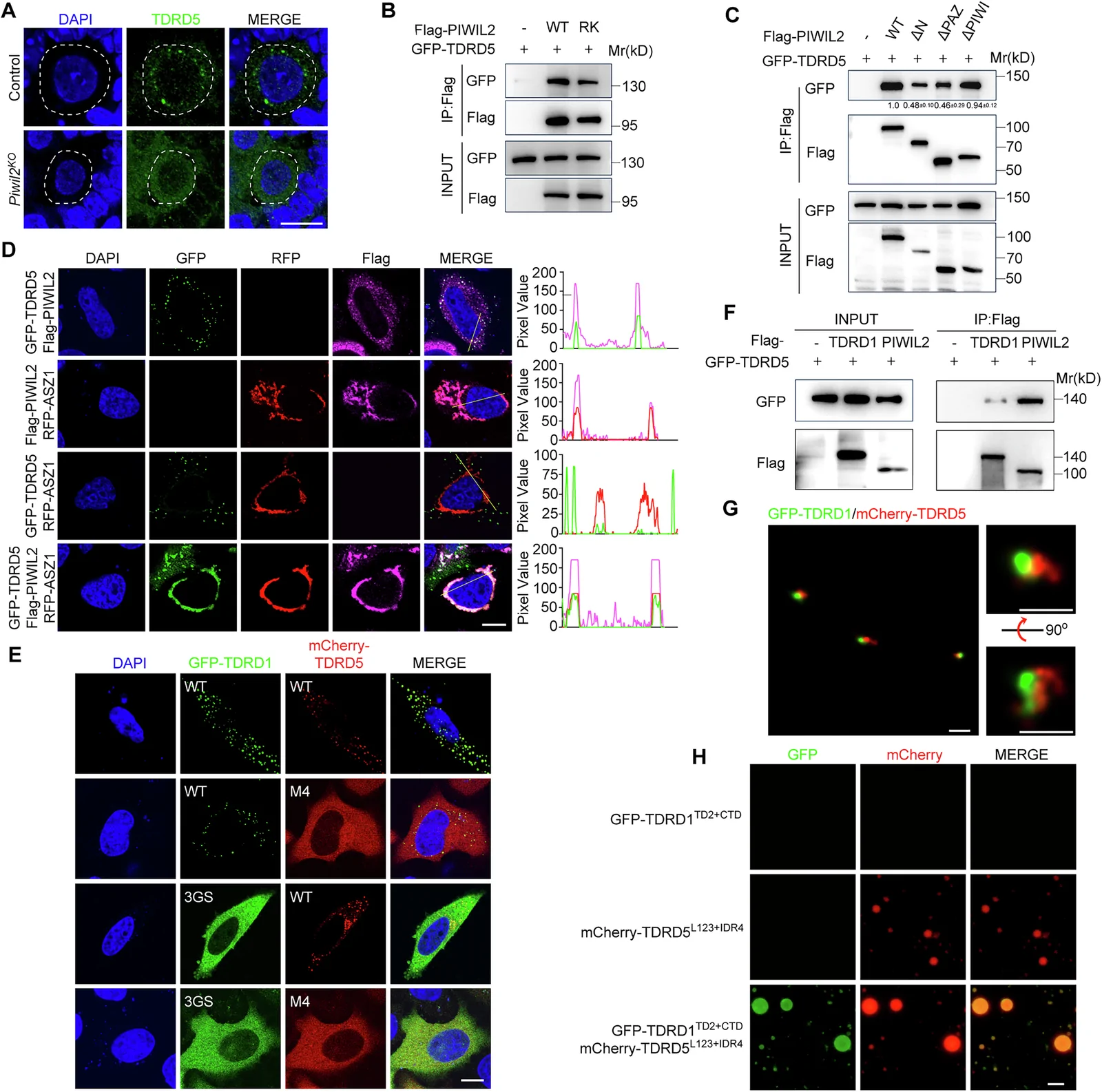
TDRD5 Is Recruited to the IMC through Interaction with PIWIL2. (**A**) Immunostaining of TDRD5 on P0 gonocytes. Scale bars, 10 μm. (**B**, **C**) Co-IP assay in 293 T cells transfected with indicated plasmids. Flag and GFP tagged proteins were detected by WB. Quantification of blot intensity of Co-IP is shown in numbers (the one from WT is set as 1.0 after normalization with IP-flag blotting). *n* = 3 (biological replicates). (**D**) Images of HeLa cells transfected with indicated plasmids. Scale bars, 10 μm. Fluorescence intensity through positions denoted by the yellow lines are shown in right. (**E**) Images of HeLa cells transfected with indicated plasmids. Scale bars, 10 μm. (**F**) Co-IP assay in 293T cells transfected with indicated plasmids. Flag and GFP tagged proteins were detected by WB. (**G**) N-storm images of HeLa cells transfected with GFP-TDRD1 and mCherry-TDRD5. Scale bars, 1 μm. (**H**) Purified recombinant mCherry-TDRD5^L123+IDR4^ and GFP-TDRD1^TD2+CTD^ in vitro. Scale bars, 10 μm. Source data are available online for this figure.

The mechanism underlying TDRD5 recruitment to the IMC was previously unclear. Given the central role of PIWIL2 in anchoring the piRNA biogenesis machinery to mitochondria in gonocytes, we hypothesized that PIWIL2 mediates TDRD5 recruitment. In support of this, TDRD5 granule localization was disrupted in *Piwil2*^−/−^ gonocytes (Fig. 7A). Co-IP assays confirmed a direct interaction between TDRD5 and PIWIL2 (Fig. 7B). To assess whether this interaction depends on arginine methylation—a modification critical for PIWIL2-TDRD1 binding—we generated a PIWIL2 RK mutant in which all nine arginine residues within the N-terminal RG/RA motif were replaced with lysines. This mutation did not affect the PIWIL2-TDRD5 interaction (Fig. 7B), indicating that arginine methylation is dispensable for TDRD5 binding. We next sought to identify the PIWIL2 domains required for TDRD5 binding using a series of truncation mutants. Co-IP assays in 293 T cells showed that deletion of either the N-terminal region or the PAZ domain, but not the PIWI domain, impaired TDRD5-PIWIL2 binding (Fig. 7C). Since the mitochondrial-anchored protein ASZ1 interacts with PIWIL2 and recruits it to the IMC (Gao et al, 2025), we co-expressed TDRD5, PIWIL2, and ASZ1 in HeLa cells and observed marked enrichment of all three proteins in mitochondrial regions (Fig. 7D). Taken together, these results demonstrate that TDRD5 is recruited to the IMC via the ASZ1-PIWIL2 complex.

### TDRD1 and TDRD5 colocalize and mutually enhance phase separation

TDRD1 and TDRD5 are both localized to the IMC and undergo phase separation (Gao et al, 2024; Yabuta et al, 2011). To investigate the potential interplay between them, we co-transfected HeLa cells with GFP-tagged TDRD1 and mCherry-tagged TDRD5. Imaging revealed near-complete colocalization of TDRD1 with TDRD5 condensates (Fig. 7E). Surprisingly, co-IP in 293 T cells showed only minimal interaction between TDRD5 and TDRD1, especially when compared to the strong TDRD5-PIWIL2 interaction (Fig. 7F). We therefore speculated that their colocalization relies on weak multivalent interactions facilitated by phase separation. Consistent with this, phase separation-deficient mutants of either TDRD1 or TDRD5 substantially disrupted their colocalization (Fig. 7E). To further examine their spatial relationship, we performed super-resolution microscopy (N-STORM). 3D surface reconstruction of GFP-TDRD1 and mCherry-TDRD5 showed that the two proteins form closely associated but non-overlapping foci (Fig. 7G).

**Figure EV8:**
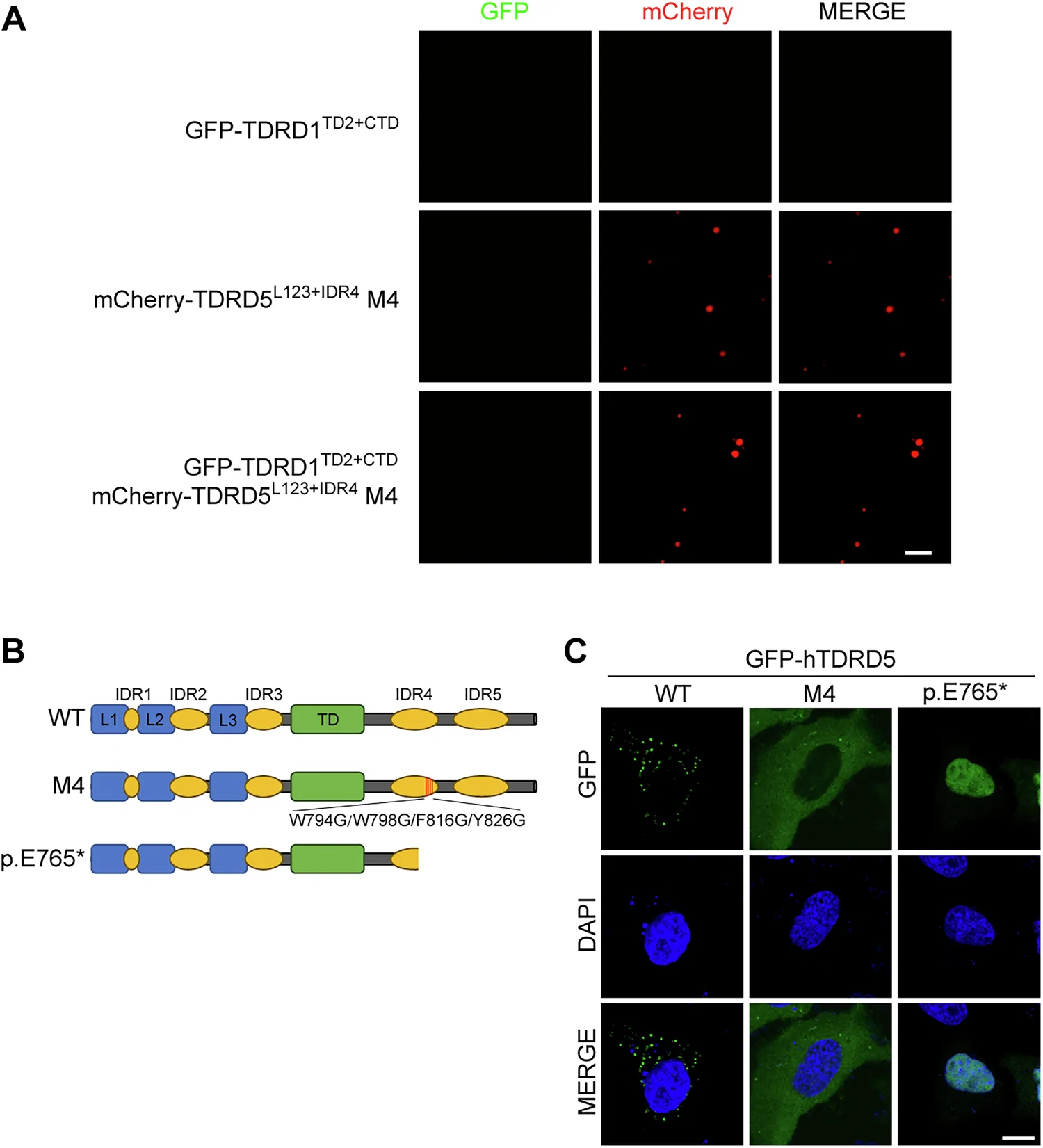
TDRD5 phase separation may play roles in human male fertility. (**A**) Droplet formation using purified recombinant mCherry-TDRD5^L123+IDR4^ M4 and GFP-TDRD1^TD2+CTD^ in vitro. Scale bars, 10 μm. (**B**) Schematic representation of human TDRD5 constructs. (**C**) Images of HeLa cells transfected with indicated human TDRD5 constructs were shown. Scale bars, 10 μm.

We next asked whether their condensation cooperates in vitro. TDRD1 phase separation is mediated by its second Tudor domain (TD2) and C-terminal domain (CTD), while TDRD5 condensation depends on its three Lotus domains (L123) and the fourth intrinsically disordered region (IDR4). We purified the recombinant proteins TDRD1^TD2+CTD^ and TDRD5^L123+IDR4^. While TDRD1^TD2+CTD^ alone failed to form droplets without crowding reagents, mixing it with TDRD5^L123+IDR4^ promoted phase separation of both proteins (Fig. 7H). Moreover, TDRD1^TD2+CTD^ enhanced the droplet formation of TDRD5^L123+IDR4^ (Fig. 7H). To investigate whether the intact phase separation capacity of TDRD5^L123+IDR4^ is required for the recruitment of the TDRD1^TD2+CTD^, we performed co-condensation assays using the TDRD5^L123+IDR4^ M4 mutant and TDRD1^TD2+CTD^. The results revealed that TDRD5^L123+IDR4^ M4 mutant completely failed to recruit the TDRD1^TD2+CTD^ to the droplets (Fig. EV8A). This suggests that the intact phase separation capacity of TDRD5 is a prerequisite for the efficient recruitment of TDRD1. Together, these data indicate that TDRD1 and TDRD5 colocalize in condensates and mutually enhance each other’s phase separation, likely through weak, multivalent intermolecular interactions.

### TDRD5 phase separation may contribute to human male fertility

The human TDRD5 protein also forms condensates in HeLa cells (Fig. EV2D), and the aromatic residues within its IDR4 region are conserved in humans (Fig. EV5). Consistent with observations in mouse TDRD5, mutations of these aromatic residues in IDR4 disrupt the ability of human TDRD5 to form condensates in cells (Fig. EV8). Interestingly, a homozygous nonsense variant (c.2293 G > T, p.E765*) in the human TDRD5 gene has been reported to cause infertility. The affected patient exhibited severe oligoasthenoteratozoospermia, characterized by the presence of abnormal spermatozoa (Guo et al, 2025). This nonsense mutation leads to partial deletion of the IDR4 region, including the four critical aromatic residues (Fig. EV8B). We therefore hypothesized that this mutation disrupts TDRD5-mediated phase separation. Indeed, the human TDRD5 p.E765* and M4 variant failed to form condensates in HeLa cells (Fig. EV8C). These results suggest that TDRD5 phase separation may play a crucial role in human male fertility.

## Discussion

We previously reported that the IMC component TDRD1 undergoes phase separation to trigger IMC formation in germ cells (Gao et al, 2024). In this study, we identified another Tudor domain protein TDRD5 which also undergoes phase separation and plays roles in IMC assembly and piRNA biogenesis. Interestingly, the molecular mechanism underlying how TDRD1 and TDRD5 exert phase separation is completely different. TDRD1 acquires phase separation activity through its C-terminal coiled-coil domain and Tudor domain. The coiled-coil domain mediates self-interaction of TDRD1, while the Tudor domain promotes phase separation by recognizing proteins with arginine methylation modifications (Gao et al, 2024; Mathioudakis et al, 2012). In contrast to TDRD1, TDRD5 represents a more “classical” phase-separation protein: it gains phase separation capability via its N-terminal tandem Lotus domains and an intrinsically disordered region (IDR). The Lotus domains possess RNA-binding activity, and the IDR of TDRD5 mediates multivalent protein interactions through aromatic amino acids. The combination of an RNA-binding domain and an IDR is a more commonly observed mechanism for conferring phase separation activity. Interestingly, the Tudor domain of TDRD5 is not essential for its phase separation, nor does TDRD5’s interaction with PIWIL2 rely on the Tudor domain’s recognition of arginine methylation sites. These findings highlight the complexity of phase separation mechanisms and the diverse functions of Tudor domain proteins.

Although both TDRD1 and TDRD5 are involved in IMC assembly and piRNA biogenesis, their functional roles in these processes are different. The phase separation activity of TDRD1 plays a more prominent role in IMC assembly. TDRD1 phase separation mutation completely abolishes IMC assembly in gonocytes, whereas loss of TDRD5 phase separation only partially impairs it. Moreover, TDRD1 phase separation is critical for IMC assembly in pachytene spermatocytes, while TDRD5 phase separation appears dispensable in this context. We propose that TDRD1 acts as the primary driver and the most essential scaffold protein for IMC assembly. In contrast, TDRD5 fine-tunes the process, possibly by modulating the phase separation behavior of TDRD1. Supporting this notion, TDRD5 significantly enhances the phase separation of TDRD1 in vitro. Notably, although TDRD1 and TDRD5 exhibit strong colocalization and mutually enhance phase separation, their direct interaction appears minimal. This apparent discrepancy suggests that their association may not rely on a stable, high-affinity protein-protein interaction. Instead, it is more consistent with a model of biophysical co-condensation, in which weak, multivalent interactions promote their cooperative partitioning into a shared condensed phase.

One remarkable finding of this study is that the phase separation activity of TDRD5 specifically affects IMC assembly and the production of fetal piRNA in gonocytes, but is dispensable for IMC assembly and pachytene piRNA biogenesis in pachytene spermatocytes. A plausible explanation is that in gonocytes, where TDRD1 expression is relatively low, TDRD5 is required to assist TDRD1 in promoting IMC assembly. In contrast, in pachytene spermatocytes, TDRD1 is abundantly expressed and can sufficiently drive IMC assembly even in the absence of TDRD5 phase separation activity. On the other hand, our previous work demonstrated that TDRD5 selectively facilitates the production of cluster-derived piRNAs in pachytene spermatocytes, possibly through its involvement in a ribosome-mediated piRNA biogenesis pathway (Sun et al, 2021; Sun et al, 2020). Clearly, the phase separation activity cannot explain the molecular role of TDRD5 in pachytene piRNA production, and the precise mechanism remains to be elucidated. In summary, TDRD5 plays distinct roles in piRNA biogenesis at different stages: during fetal piRNA generation, it promotes IMC assembly via its phase separation activity to enhance piRNA production efficiency; whereas in pachytene piRNA biogenesis, it employs an unknown mechanism to selectively promote the processing of cluster-derived piRNAs.

Through systematic screening of piRNA-related proteins, we have identified three proteins with significant droplet-forming capabilities: TDRD1, TDRD5, and RNF17. We have demonstrated that TDRD1 and TDRD5 localize to the IMCs and promote IMC assembly as well as piRNA production through phase separation. RNF17 is another Tudor domain-containing protein primarily localized to processing bodies in germ cells. During pachytene piRNA biogenesis, RNF17 maintains normal piRNA production by suppressing the activity of the ping-pong cycle (Wasik et al, 2015). Whether RNF17 possesses phase separation activity, as well as the molecular mechanisms underlying its role in piRNA biogenesis and function, requires further investigation.\

## Methods

**Table:** Reagents and tools table

Reagent/resource	Reference or source	Identifier or catalog number
**Experimental models**		
*Piwil2*^*−/−*^ mice	Gao et al, 2024	N/A
*Tdrd5*^*−/−*^ mice	This paper	N/A
*Tdrd5*^*M4/-*^ mice	This paper	N/A
Human: 293T	TCCCAS	SCSP-502
Human: HeLa	TCCCAS	SCSP-504
Human: U2OS	TCCCAS	SCSP-503
Escherichia coli DH5α Chemically Competent Cell	Tsingke	Cat# TSC-C01
Escherichia coli BL21 (DE3) Chemically Competent Cell	Tsingke	Cat# TSC-E01
**Recombinant DNA**		
See Table EV1		
**Oligonucleotides**		
Cy3-UUAGGGUUAGGGUUAGGGUUAGGG	Sangon	N/A
Cy3-UUACCGUUACCGUUACCGUUACCG	Sangon	N/A
**Antibodies**		
Rabbit polyclonal anti-PIWIL2	MBL	Cat# PM044, RRID: AB_1279201
Rabbit polyclonal anti-PIWIL4	Gao et al, 2024	N/A
Rabbit polyclonal anti-TDRD5	Ding et al, 2018	N/A
GAPDH Monoclonal antibody	Proteintech	Cat# 21550-1-AP, RRID: AB_10858622
mouse polyclonal anti-PIWIL2	Santa Cruz	Cat# sc-377258
Rabbit polyclonal anti-LINE1 ORF1	Ding et al, 2017	N/A
Mouse FITC-conjugated anti-γH2AX	Millipore	Cat# 16-202 A, RRID: AB_568825
Rabbit polyclonal anti-ACRV1	Proteintech	Cat# 14040-1-AP, RRID: AB_10640426
HRP-conjugated Mouse monoclonal anti-Flag	ABclonal	Cat# AE024, RRID: AB_2769864
Rabbit monoclonal anti-GFP	ABclonal	Cat# AE078
Mouse monoclonal anti-Flag	ABclonal	Cat# AE005, RRID: AB_2770401
**Chemicals, enzymes and other reagents**		
Glutathione Sepharose beads	Smart-Lifesciences	Cat# SA008005
Proteinase inhibitor cocktail	ThermoFisher	Cat# A32963
Ni-TED Sefinose (TM) Resin 6FF	Sangon	Cat# C610030-0010
Protein A/G Agarose Beads	Beyotime	Cat# P2055
1, 6-Hexanediol	Millipore	Cat# 240117
2, 5-Hexanediol	Millipore	Cat# H11904
TRIzol	ThermoFisher	Cat# 15596026
EZ Trans	Life-iLab	Cat# AC04L092
jetPRIME	Polyplus	Cat# 101000046
Hematoxylin and Eosin	Beyotime	Cat# E607318-0200
PreScission Protease	Beyotime	Cat# P2303
Antifade Mounting Medium with DAPI	Beyotime	Cat# P0131
**Software and algorithms**		
Cutadapt v4.4		https://pypi.org/project/cutadapt/4.4/
Bowtie v1.0.0		https://sourceforge.net/projects/bowtie-bio/files/bowtie/1.0.0/
GraphPad Prism	GraphPad Software	https://www.graphpad.com
PONDR		https://www.pondr.com
Clustal Omega		https://www.clustal.org/lander
ImageJ	NIH	https://imagej.nih.gov/ij/
**Other**		
ClonExpress Ultra One Step Cloning Kit	Vazyme	Cat# C115-01
VAHTS Small RNA Library Prep Kit for Illumina V2	Vazyme	Cat# NR811-01

### Mice

To generate *Tdrd5* knockout (*Tdrd5*^*−/−*^) mice, a conditional *Tdrd5*-floxed (*Tdrd5*^*fl*^) allele was first created in C57BL/6 embryos using CRISPR/Cas9-mediated genome editing, in which exon 4 was flanked by loxP sites. *Tdrd5*^*fl/+*^ mice were then crossed with mice expressing Cre recombinase under the control of the germ cell-specific Stra8 promoter (*Stra8*-GFP-Cre) (Chen et al, 2018) to obtain *Tdrd5* heterozygous (*Tdrd5*^*+/−*^) mice. Finally, *Tdrd5*^*+/−*^ males and females were intercrossed to yield *Tdrd5*^*−/−*^ homozygous knockout mice. *Piwil2* knockout (*Piwil2*^*−/−*^) mice were generated as described previously (Gao et al, 2024).

*Tdrd5*^*M4*^ mutant mice were generated in a C57BL/6 background using CRISPR/Cas9-mediated genome editing by Cyagen Biosciences. Founder (F0) animals were genotyped by PCR amplification and subsequent DNA sequencing. Heterozygous *Tdrd5*^*M4/+*^ mice were then crossed with *Tdrd5*^*+/−*^ mice to produce *Tdrd5*^*M4/−*^ offspring for phenotypic analysis.

This study was conducted in accordance with institutional guidelines and ethical regulations, with all experimental protocols approved by the Institutional Animal Care and Use Committee (IACUC) of Tongji University. All mice were housed under specific pathogen-free (SPF) conditions in the university’s animal facility, where they received standard rodent chow and water ad libitum, with routine care provided by facility staff. A 12-h light/dark cycle was maintained throughout the study period.

### Cell culture and transfection

HeLa, U2OS and 293T cell lines were acquired from the Cell Bank of the Type Culture Collection of the Chinese Academy of Sciences (TCCCAS). Cells were cultured in DMEM (Corning) supplemented with 10% fetal bovine serum and 1% penicillin–streptomycin at 37 °C under a humidified atmosphere containing 5% CO₂. Transfection was carried out using EZ Trans (Life-iLab) for 293T cells and jetPRIME (Polyplus) for HeLa and U2OS cells, in accordance with the manufacturers’ protocols.

### Plasmids

The full-length cDNAs of mouse ASZ1, GPAT2, mitoPLD, TDRKH, TDRD1, RNF17, TDRD5, TDRD6, TDRD7, TDRD9, TDRD12, EXD1, ADAD2, PIWIL1, PIWIL2, PIWIL4, MVH, MOV10L1, GTSF1, MAEL, PNLDC1, HENMT1, FKBP6, HSP90a were amplified by PCR. The DNA fragments were then cloned into expression vectors using the ClonExpress Ultra One Step Cloning Kit (Vazyme, C115-01). Eukaryotic expression vectors used in this study were: pcDNA3-Flag (N-terminal Flag tag), pcDNA3-RFP (N-terminal TurboRFP tag), pmCherry (N-terminal mCherry tag), pCRY2PHR-mCherry (CRY2-mCherry at N-terminal), pEGFP-C1 (N-terminal GFP tag) and pEGFP-N1 (C-terminal GFP tag). The prokaryotic expression vectors used in this study contain pET-28a-GFP (HIS-GFP at N-terminal) and pGEX-6P-1-GFP (GST-GFP at N-terminal). All oligonucleotide primers were synthesized by Tsingke Biotech (Beijing, China), and all plasmid constructs were verified by Sanger sequencing. Additional details regarding plasmid construction are provided in Table EV1.

### Immunostaining of mouse testes

Testes from postnatal day 0 (P0) and adult mice were collected and fixed overnight at 4 °C in 4% paraformaldehyde (PFA) prepared in phosphate-buffered saline (PBS), followed by paraffin embedding. Sections of 5 μm thickness were prepared, dewaxed, and rehydrated. Antigen retrieval was carried out by microwave treatment in 0.01 M Tris-EDTA buffer (pH 9.0) for 2 min. After rinsing with PBS, tissue sections were blocked with 5% normal goat serum (NGS) for 30 min. Subsequently, the sections were incubated with primary antibodies diluted in 5% NGS at 37 °C for 1 h. Following PBS washes, the sections were incubated with fluorescently labeled secondary antibodies at room temperature for 1 h and mounted using an antifade mounting medium containing DAPI (Beyotime). Imaging was performed with a FV3000 inverted confocal microscope (Olympus, Japan). The following primary antibodies were used for immunofluorescence: rabbit anti-TDRD5 (1:200; homemade) (Ding et al, 2018), rabbit anti-LINE1 Orf1 (1:500; homemade) (Gao et al, 2024), rabbit anti-PIWIL2 (1:200; PM044, MBL), rabbit anti-PIWIL4 (1:200; homemade) (Gao et al, 2024) and FITC-conjugated mouse anti-γH2AX (1:500; 16-202A, Millipore).

### In vitro droplet formation assay

Purified proteins (His-GFP-TDRD1^TD2+CTD^, GFP-TDRD5^L123+IDR4^, GFP-TDRD5^L123+IDR4^-M4, mCherry-TDRD5^L123+IDR4^) were diluted in phase-separation buffer (20 mM HEPES, pH 7.5) containing specified NaCl concentrations. Samples were loaded into glass sandwich chambers with double-sided spacers and imaged for droplet formation using an Olympus CKX53 inverted microscope (Olympus, Japan).

### Hexanediol treatment

GFP-TDRD5 transfected HeLa cells cultured in glass-bottom plates were exposed to PBS (control), 1,6-hexanediol, or 2,5-hexanediol at indicated concentrations (2% in this study) for 10 min at 37 °C. Following treatment, cells were fixed with 4% paraformaldehyde (PFA) for subsequent immunostaining. Fluorescence images were acquired using a FV3000 inverted confocal microscope (Olympus, Japan).

### Computer-assisted semen analysis (CASA)

To assess sperm motility, the caudal epididymis was collected immediately post-euthanasia. The epididymal tail was subjected to two incisions and immersed in 500 μL of TYH medium (Nanjing AIBI Bio-Tech). After a 15 min incubation at 37 °C, sperm motility parameters were measured with the Hamilton Thorne Ceros II system (Beverly, MA, USA).

### Fluorescence recovery after photobleaching (FRAP)

HeLa cells were seeded into 35 mm glass-bottom imaging dishes and transfected with GFP-TDRD5 expression constructs. After 24 h, fluorescence recovery after photobleaching was performed using an Olympus SpinSR10 spinning disk super-resolution microscope equipped with a live-cell environmental chamber (Tokai Hit; 37 °C, 5% CO₂, 85% humidity). Selected regions of interest (ROIs) were photobleached with 488 nm laser illumination (5 iterations, 30% intensity, 700 μs dwell time). Post-bleach recovery was monitored over 120 s at 10 s intervals. GFP fluorescence intensity quantification was conducted using ImageJ software.

GFP-TDRD5^L123+IDR4^ were diluted in phase-separation buffer (20 mM HEPES, pH 7.5) containing 150 mM NaCl concentrations. Samples were loaded into glass sandwich chambers with double-sided spacers. Fluorescence recovery after photobleaching was performed using a FV3000 inverted confocal microscope (Olympus, Japan). Post-bleach recovery was monitored over 104 s at 8 s intervals. GFP fluorescence intensity quantification was conducted using ImageJ software.

### OptoDroplet assay

The OptoDroplet assay was conducted following established methodology (Zhang et al, 2019). Briefly, DNA fragments were cloned into the pCRY2PHR-mCherry vector. 293T cells plated in 35 mm glass-bottom dishes were transfected with specified constructs. At 36 h post-transfection, liquid-liquid phase separation was optically induced using 488 nm blue light (160 s exposure). mCherry fluorescence was excited at 561 nm. Live-cell imaging was performed on a Nikon Ti2-E inverted confocal microscope (Nikon, Japan).

### Fixed cell imaging

HeLa, 293T, and U2OS cell lines were transfected with specified fluorescent protein-tagged expression vectors. Transfected cells grown on coverslips were fixed with 4% paraformaldehyde (PFA) in phosphate-buffered saline (PBS) for 10 min at ambient temperature. Following fixation, cells were permeabilized with 0.5% Triton X-100 in PBS (5 min). Nuclei were counterstained using DAPI-containing antifade mounting medium (Beyotime). Fluorescent signals (GFP, mCherry) were imaged on Ti2-E inverted confocal microscope (Nikon, Japan) or FV3000 inverted confocal microscope (Olympus, Japan). For quantitative analysis, GFP-positive puncta were enumerated in randomly selected cells per experimental condition using ImageJ software.

### Quantification of relative protein expression levels

To evaluate the relative expression levels of the 24 overexpressed proteins, fluorescence intensity was quantified from the acquired micrographs by calculating the Corrected Total Cell Fluorescence (CTCF) using ImageJ/Fiji software (NIH). For each protein construct, whole-cell regions of randomly selected cells were manually outlined to measure the cell area and integrated density. To account for background signal, three distinct cell-free regions adjacent to the measured cells were selected to calculate the mean background fluorescence. The CTCF for each individual cell was subsequently determined using the following formula: CTCF = integrated density – (area of selected ×  mean fluorescence of background). The calculated CTCF values were then plotted to compare the relative expression levels across the different protein constructs.

### STORM imaging

For direct STORM imaging, experiments were performed using both Olympus IX-81 and Nikon N-STORM microscopes (Tokyo, Japan). Samples were illuminated with a 642 nm diode laser (~20 mW at the back focal plane of the objective) with an exposure time of 50 ms per frame and a pixel size of 85 nm. Time-series TIRF images were acquired and subsequently reconstructed to generate super-resolution images. An oxygen-scavenging solution consisting of 1 mg glucose oxidase, 1.2 μl catalase (from bovine liver), 4 mM TCEP, 5.2 mM KCl, 2 mM Tris-HCl (pH 7.5), 0.5 ml glycerol, and 0.45 ml H₂O was prepared, and 20 μl of this solution was added to a 35-mm imaging chamber. The chamber was then filled to capacity with filter-sterilized PBS (pH 7.4).

### Western blotting (WB)

Protein extracts were prepared from harvested 293 T cells and mouse testes using lysis buffer (150 mM NaCl, 50 mM Tris-HCl pH 7.4, 1% Triton X-100, 1 mM EDTA, plus protease inhibitors). Following 30 min incubation on ice, lysates were clarified by centrifugation (12,000 rpm, 10 min, 4 °C). Supernatants were denatured in SDS-loading buffer at 100 °C for 5 min prior to SDS-PAGE electrophoresis. Immunoblotting employed the following primary antibodies: anti-TDRD5 (1:1000; homemade), anti-LINE1 Orf1 (1:5000; homemade), anti-Flag (1:1000; ABclonal AE005), anti-GFP (1:1000; Beyotime AF1483) and anti-GAPDH (1:20000; Proteintech, 60004-1-Ig). Protein signals were detected using chemiluminescence on a Tanon-5200 Imaging System.

### Co-immunoprecipitation (Co-IP)

293T cells underwent transient transfection with specified plasmids using EZ-trans (Life-iLab). Post-transfection, cells were collected, PBS-washed, and lysed in ice-cold buffer (150 mM NaCl, 50 mM Tris-HCl pH 7.4, 1% Triton X-100, 1 mM EDTA, plus protease inhibitors). Following 30-min incubation on ice, lysates were centrifuged (12,000 rpm, 10 min, 4 °C). Supernatants were subjected to overnight co-immunoprecipitation at 4 °C with Protein A/G beads coupled to target antibodies. Bead complexes were then washed thrice with chilled wash buffer (300 mM NaCl, 50 mM Tris-HCl pH 7.4, 1% Triton X-100, 1 mM EDTA, plus protease inhibitors), resuspended in SDS-loading buffer, and denatured at 100 °C for 5 min prior to SDS-PAGE. Immunoblots were probed with designated antibodies.

### Protein purification

For affinity purification of GST-tagged proteins, pGEX-6P-1 constructs harboring GST-GFP-TDRD5^L123+IDR4^, GST-GFP-TDRD5^L123+IDR4^-M4 and GST-mCherry-TDRD5^L123+IDR4^ were introduced into *E.coli* BL21 (Tsingke) via transformation. Bacterial cultures were grown until reaching OD values of 0.6–0.8, followed by overnight induction at 16 °C with 0.2 mM IPTG (Beyotime). Cells were pelleted and lysed through brief sonication in lysis buffer (300 mM NaCl, 25 mM Tris-HCl pH 7.4, 2 mM DTT, 1 mM PMSF). After centrifugation (12,000 rpm, 30 min, 4 °C), the clarified supernatant was affinity-purified using pre-equilibrated Glutathione Agarose Resin (Smart-Lifescience). The GST tag was removed using PreScission Protease (Beyotime). Proteins were then further resolved by size-exclusion chromatography on a Superdex-200 column (GE Healthcare) using storage buffer (1 M NaCl, 20 mM HEPES pH 7.4, 1 mM DTT). Final protein purity was verified through SDS-PAGE with Coomassie Blue staining.

### H&E staining

Mouse testicular and epididymal tissues were fixed immediately after harvest by immersion overnight at 4 °C in Bouin’s solution. Subsequently, the fixed tissues were processed for paraffin embedding. Tissue sections (5 μm thickness) were cut from the paraffin blocks, subjected to deparaffinization and rehydration procedures, and then underwent staining with Hematoxylin and Eosin (H&E) using reagents from Beyotime, strictly following the manufacturer’s protocol.

### Centrifugation-based supernatant depletion assay

To quantify the apparent saturation concentration of TDRD5^L123+IDR4^ and evaluate the effect of TERRA RNA on its phase separation, a centrifugation-based sedimentation assay was performed. Purified TDRD5^L123+IDR4^ protein was diluted to a constant final concentration of 2.5 μM in the phase separation buffer. TERRA RNA was then added to the protein solutions at varying final concentrations ranging from 0 to 1000 nM. A negative control containing only TERRA RNA (1000 nM) without the protein was prepared in parallel to rule out background interference. The mixtures were thoroughly mixed and incubated at room temperature for 30 min to allow the condensates to fully assemble and reach thermodynamic equilibrium. Following incubation, the samples were centrifuged at 12,000×*g* for 15 min at room temperature to separate the condensed phase (pellet) from the dilute phase (supernatant). The supernatant, containing the soluble protein fraction, was carefully transferred to a new tube. The concentration of the protein remaining in the supernatant was determined using the BCA Protein Assay Kit (Beyotime). The apparent Csat was defined as the plateau concentration of the soluble protein in the supernatant. All assays were performed in triplicate, and data were plotted as soluble protein concentration versus RNA concentration.

### Transmission electron microscopy (TEM)

Mouse testes were fixed immediately after harvest by immersion overnight at 4 °C in 2.5% glutaraldehyde prepared in 0.2 M phosphate buffer (pH 7.0). Following primary fixation, specimens underwent post-fixation for 1 h at room temperature using a solution containing 1% osmium tetroxide and 2% potassium ferricyanide. Subsequently, dehydration was performed through a graded ethanol series. The dehydrated samples were then infiltrated with resin and polymerized at 60 °C for 48 h. Ultrathin sections were collected onto copper grids, sequentially stained with uranyl acetate followed by lead citrate, and examined using a Tecnai Spirit transmission electron microscope (FEI, USA) operating at an accelerating voltage of 120 kV.

### Small RNA libraries and bioinformatic analyses

Small RNA libraries were prepared from total RNA utilizing the VAHTS Small RNA Library Prep Kit for Illumina V2 (Vazyme) in accordance with the manufacturer’s protocol. Pooled libraries containing unique barcodes were subsequently sequenced on an Illumina NovaSeq 6000 platform (Novogene, China). Raw sequencing reads underwent adapter trimming with Cutadapt. These size-selected reads were then aligned against multiple genomic annotation categories including piRNA clusters, protein-coding RNAs, non-coding RNAs (ncRNAs), RepeatMasker-classified repetitive elements, intronic regions, and other sequences using Bowtie with a tolerance of one base mismatch (Ding et al, 2018). To enable cross-library comparisons, small RNA read counts were normalized using miRNA abundance (21–23 nt) derived from the same sequencing data.

### Quantification and statistical analysis

The sample sizes (*n*) for biological replicates or mice are provided in the respective figure legends. No randomization was implemented, and sample sizes were not predetermined using statistical methods. No blinding was performed during the experiments or data analysis, as the genotypes of the samples were required for experimental grouping and interpretation. Data analysis was performed using Welch’s *t* test. All data are presented as mean ± SEM. Statistical significance was defined as follows: ***P < 0.001; **P < 0.01; *P < 0.05; ns, not significant. All statistical analyses were performed using GraphPad Prism software.

## Supplementary information

Table EV1

Peer Review File

Source data Fig. 1

Source data Fig. 2

Source data Fig. 3

Source data Fig. 4

Source data Fig. 5

Source data Fig. 6

Source data Fig. 7

Expanded View Figures

## Data Availability

Data files of small RNA-seq have been uploaded to CNCB with an accession number CRA031313. The source data of this paper are collected in the following database record: biostudies:S-SCDT-10_1038-S44319-026-00890-6.
