# Supplementary material for: TDRD5 facilitates piRNA biogenesis and male fertility through phase separation
Source: EMBO Rep. 2026 Aug 4;27(18):5292–316. doi: 10.1038/s44319-026-00890-6 (PMC13601554; doi:10.1038/s44319-026-00890-6)
Supplement: Supplementary file 1 [file 44319_2026_890_MOESM10_ESM.pdf]

Expanded View Figures

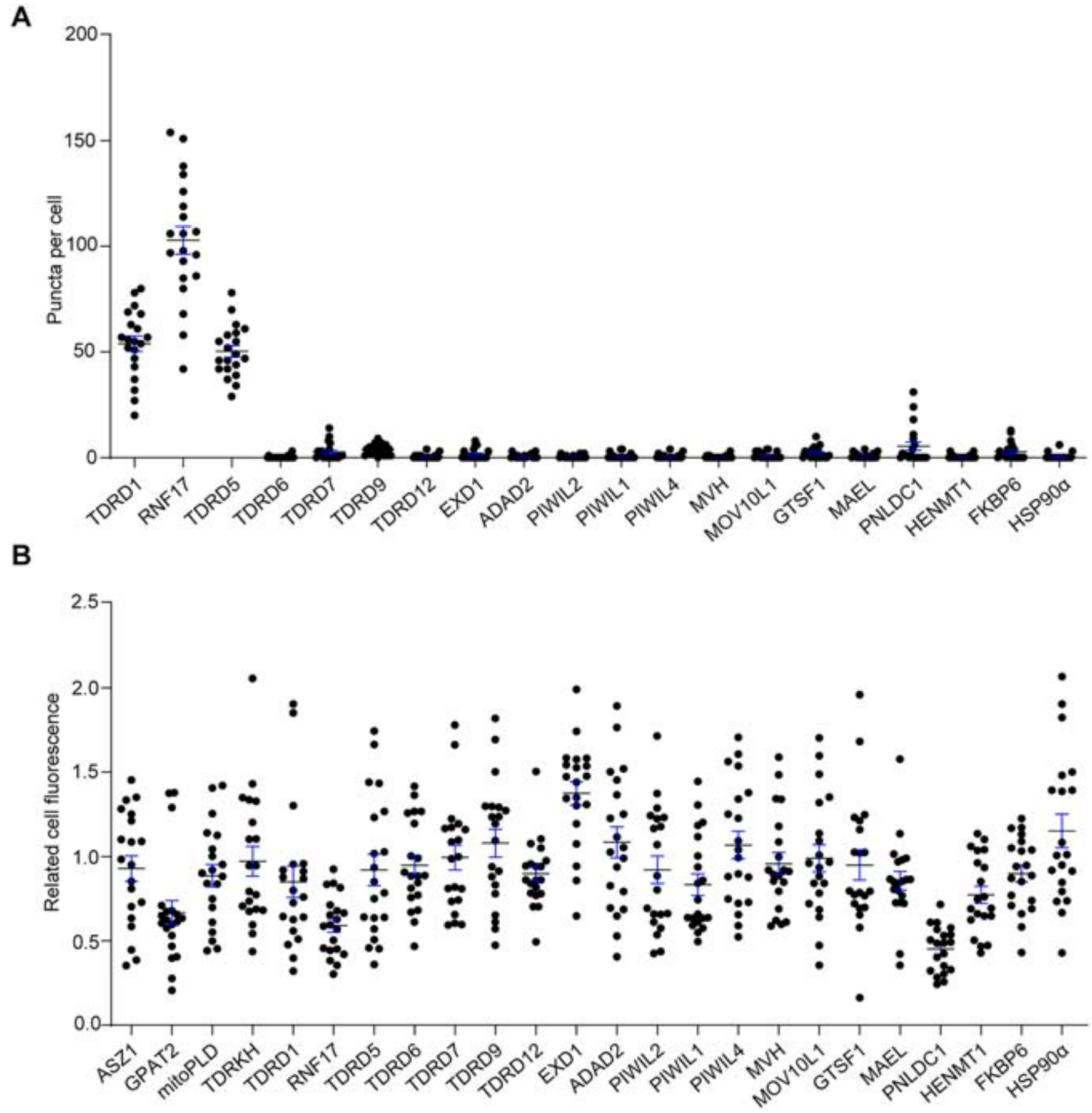

Figure EV1. Quantification of 24 overexpressed proteins in HeLa cells.

(**A**) Quantification of the puncta number per cell were shown in transfected HeLa cells. $n = 20$ cells. The data represent the mean ± s.e.m. (**B**) Quantification of the relative expression levels for the 24 overexpressed proteins in HeLa cells, measured as Corrected Total Cell Fluorescence (CTCF). $n = 20$ cells. The data represent the mean ± s.e.m.

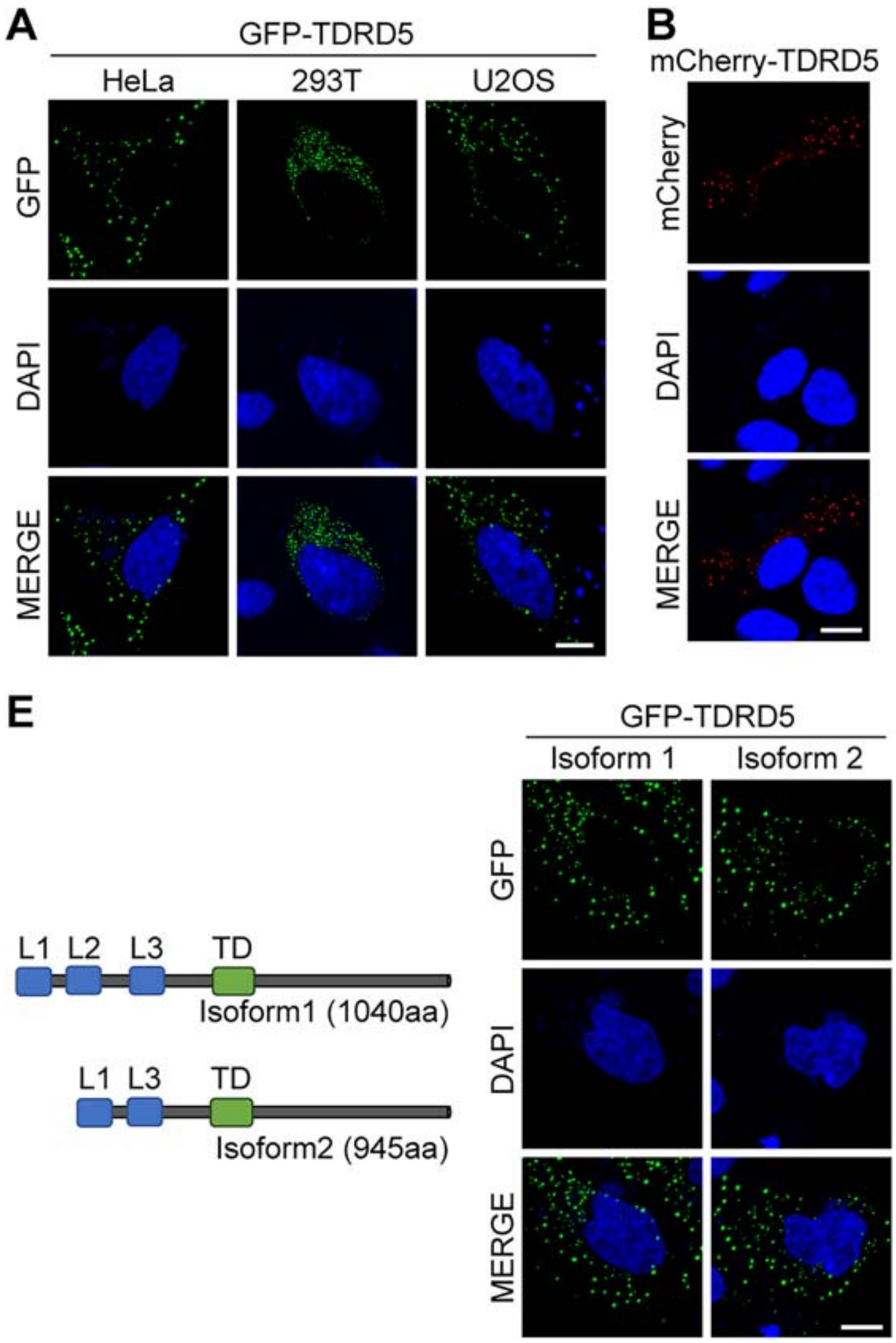

Figure EV2. TDRD5 assembles fluid-like macromolecular condensates in cells.

(**A**) Images of HeLa, 293T and U2OS cells transfected with GFP-TDRD5. Scale bars, 10 μm. (**B**) Images of HeLa cells transfected with mCherry-TDRD5. Scale bars, 10 μm. (**C**) Images of HeLa cells transfected with TDRD5-GFP. Scale bars, 10 μm. (**D**) Images of HeLa cells transfected with GFP tagged human TDRD5. Scale bars, 10 μm. (**E**) Images of HeLa cells transfected with GFP-TDRD5 Isoform 1 and 2. Scale bars, 10 μm.

 © The Author(s)

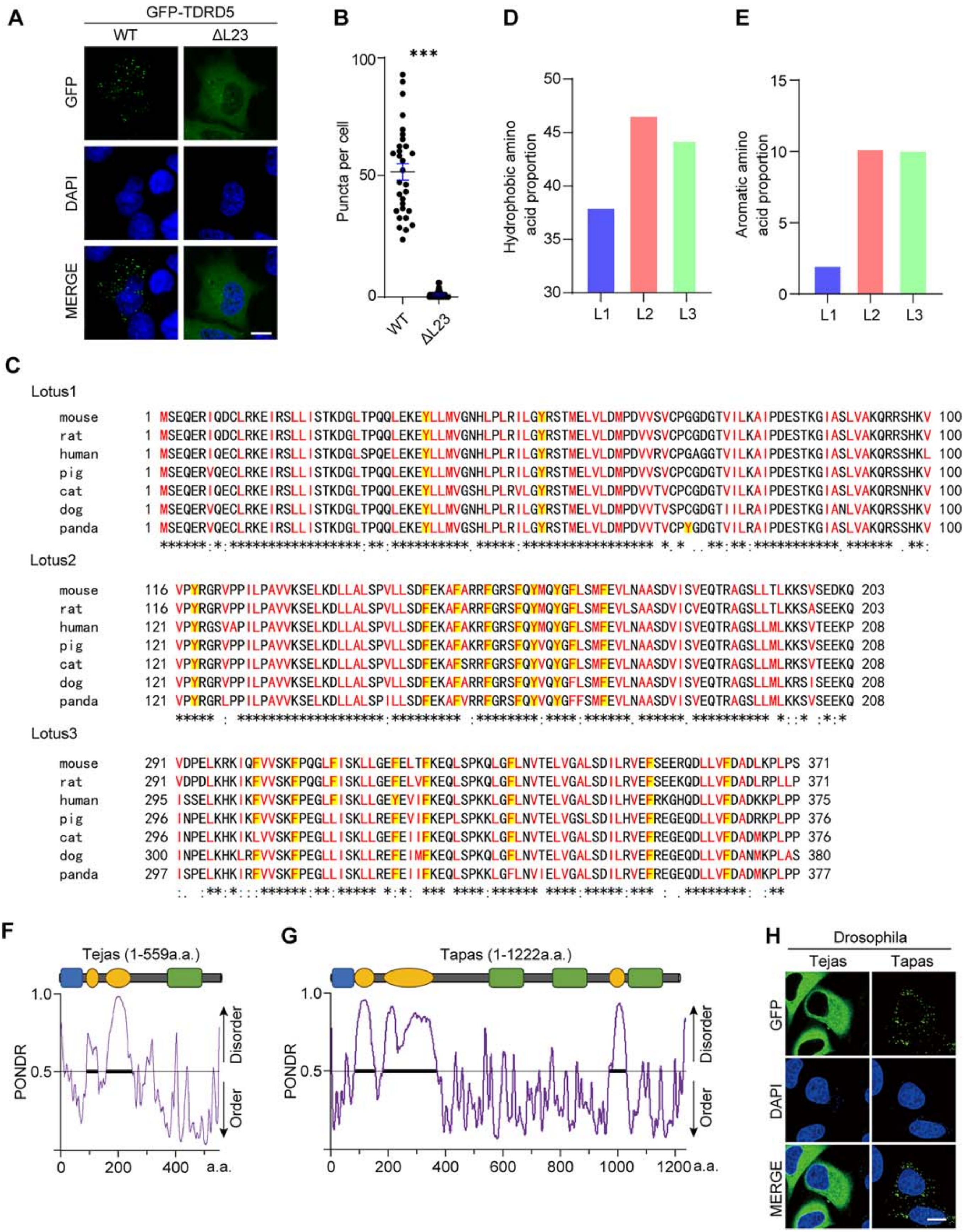
A
GFP-TDRD5
WT
ΔL23
GFP
DAPI
MERGE
B
***
100
50
0
Puncta per cell
WT
ΔL23
D
50
45
40
35
30
Hydrophobic amino acid proportion
L1
L2
L3
E
15
10
5
0
Aromatic amino acid proportion
L1
L2
L3
C
Lotus1
mouse 1 MSEQERIQDCLRKEIRSLLISTKDGLTPQQLEKEYLLMVGNHLPLRILGYRSTMELVLDMPDVVSVCPGGDGTVILKAIPDESTKGIASLVAKQRRSHKV 100
rat 1 MSEQERIQDCLRKEIRSLLISTKDGLTPQQLEKEYLLMVGNHLPLRILGYRSTMELVLDMPDVVSVCPCGDGTVILKAIPDESTKGIASLVAKQRRSHKV 100
human 1 MSEQERIQECLRKEIRSLLISTKDGLSPQELEKEYLLMVGNHLPLRILGYRSTMELVLDMPDVVRVCPGAGGTVILKAIPDESTKGIASLVAKQRSSHKL 100
pig 1 MSEQERVQECLRKEIRSLLISTKDGLTPQQLEKEYLLMVGNHLPLRILGYRSTMELVLDMPDVVSVCPCGDGTVILKAIPDESTKGIASLVAKQRSSHKV 100
cat 1 MSEQERIQECLRKEIRSLLISTKDGLTPQQLEKEYLLMVGSHLPLRVLGYRSTMELVLDMPDVVTVCPCGDGTVILKAIPDESTKGIASLVAKQRSNHKV 100
dog 1 MSEQERVQECLRKEIRSLLISTKDGLTPQQLEKEYLLMVGNHLPLRILGYRSTMELVLDMPDVVTVSPCGDGTIILRAIPDESTKGIANLVAKQRSSHKV 100
panda 1 MSEQERVQECLRKEIRSLLISTKDGLTPQQLEKEYLLMVGSHLPLRILGYRSTMELVLDMPDVVTVCPYGDGTVILRAIPDESTKGIASLVAKQRSSHKV 100
Lotus2
mouse 116 VPYRGRVPPILPAVVKSELKDLLALSPVLLSDFEKAFARRFGRSFQYMQYGFLSMFEVLNAASDVISVEQTRAGSLLTLKKSVSEDKQ 203
rat 116 VPYRGRVPPILPAVVKSELKDLLALSPVLLSDFEKAFARRFGRSFQYMQYGFLSMFEVLSAASDVICVEQTRAGSLLTLKKSASEEKQ 203
human 121 VPYRGSVAPILPAVVKSELKDLLALSPVLLSDFEKAFAKRFGRSFQYMQYGFLSMFEVLNAASDVISVEQTRAGSLLMLKKSVTEEKP 208
pig 121 VPYRGRVPPILPAVVKSELKDLLALSPVLLSDFEKAFAKRFGRSFQYVQYGFLSMFEVLNAASDVISVEQTRAGSLLMLKKSVSEEKQ 208
cat 121 VPYRGRVPPILPAVVKSELKDLLALSPVLLSDFEKAFSRRFGRSFQYVQYGFLSMFEVLNAASDVISVEQTRAGSLLMLRKSVTEEKQ 208
dog 121 VPYRGRVPPILPAVVKSELKDLLALSPVLLSDFEKAFARRFGRSFQYVQYGFLSMFEVLNAASDVISVEQTRAGSLLMLKRSISEEKQ 208
panda 121 VPYRGRLPPILPAVVKSELKDLLALSPILLSDFEKAFVRRFGRSFQYVQYGFFSMFEVLNAASDVISVEQTRAGSLLMLKKSVSEEKQ 208
Lotus3
mouse 291 VDPELKRKIQFVVSKFPQGLFISKLLGEFELTFKEQLSPKQLGFLNVTELVGALSDILRVEFSEERQDLLVFDADLKPLPS 371
rat 291 VDPDLKHKIKFVVSKFPQGLFISKLLGEFELVFKEQLSPKQLGFLNVTELVGALSDILRVEFSEEKQDLLVFDADLRPLLP 371
human 295 ISSELKHKIKFVVSKFPEGLFISKLLGEYEVIFKEQLSPKKLGFLNVTELVGALSDILHVEFRKGHQDLLVFDADKKPLPP 375
pig 296 INPELKHKIKFVVSKFPEGLLISKLLREFEVIFKEPLSPKKLGFLNVTELVGSLSDILHVEFREGEQDLLVFDADRKPLPP 376
cat 296 INPELKHKIKLVVSKFPEGLLISKLLGEFEIIFKEQLSPKKLGFLNVTELVGALSDILRVEFREGEQDLLVFDADMKPLPP 376
dog 300 INPELKHKLRFVVSKFPEGLLISKLLREFEIMFKEQLSPKQLGFLNVTELVGALSDILRVEFREGEQDLLVFDANMKPLAS 380
panda 297 ISPELKHKIRFVVSKFPEGLLISKLLREFEIIFKEQLSPKKLGFLNVIELVGALSDILRVEFREGEQDLLVFDADMKPLPP 377
F
Tejas (1-559a.a.)
1.0
0.5
PONDR
Disorder
Order
0
200
400
a.a.
G
Tapas (1-1222a.a.)
1.0
0.5
PONDR
Disorder
Order
0
200
400
600
800
1000
1200
a.a.
H
Drosophila
Tejas
Tapas
GFP
DAPI
MERGE

Figure EV3. Lotus domains and IDR4 drive TDRD5 condensation.

(**A**) Images of HeLa cells transfected with indicated plasmids. Scale bars, 10 µm. (**B**) Quantification of the puncta number per cell were shown in transfected HeLa cells. $n = 30$ cells. The data represent the mean ± s.e.m. ***$P = 3.48374E\text{-}15$. P values were calculated using Welch's t test. (**C**) Multiple sequence alignment of Lotus domains of TDRD5 from mouse, human, rat, panda, cat, pig and dog using Clustal Omega. The hydrophobic amino acids were shown in red. The aromatic amino acids were highlighted in yellow. (**D**) Proportion of hydrophobic amino acids in three Lotus domains of TDRD5. (**E**) Proportion of Aromatic amino acids in three Lotus domains of TDRD5. (**F**, **G**) Intrinsic disorder prediction and domain mapping of Tejas (**A**) and Tapas (**B**): PONDR VSL2 disorder profile (bottom) and annotated domain schematic (top). (**H**) Images of HeLa cells transfected with Tejas and Tapas were shown. Scale bars, 10 µm.

 © The Author(s)

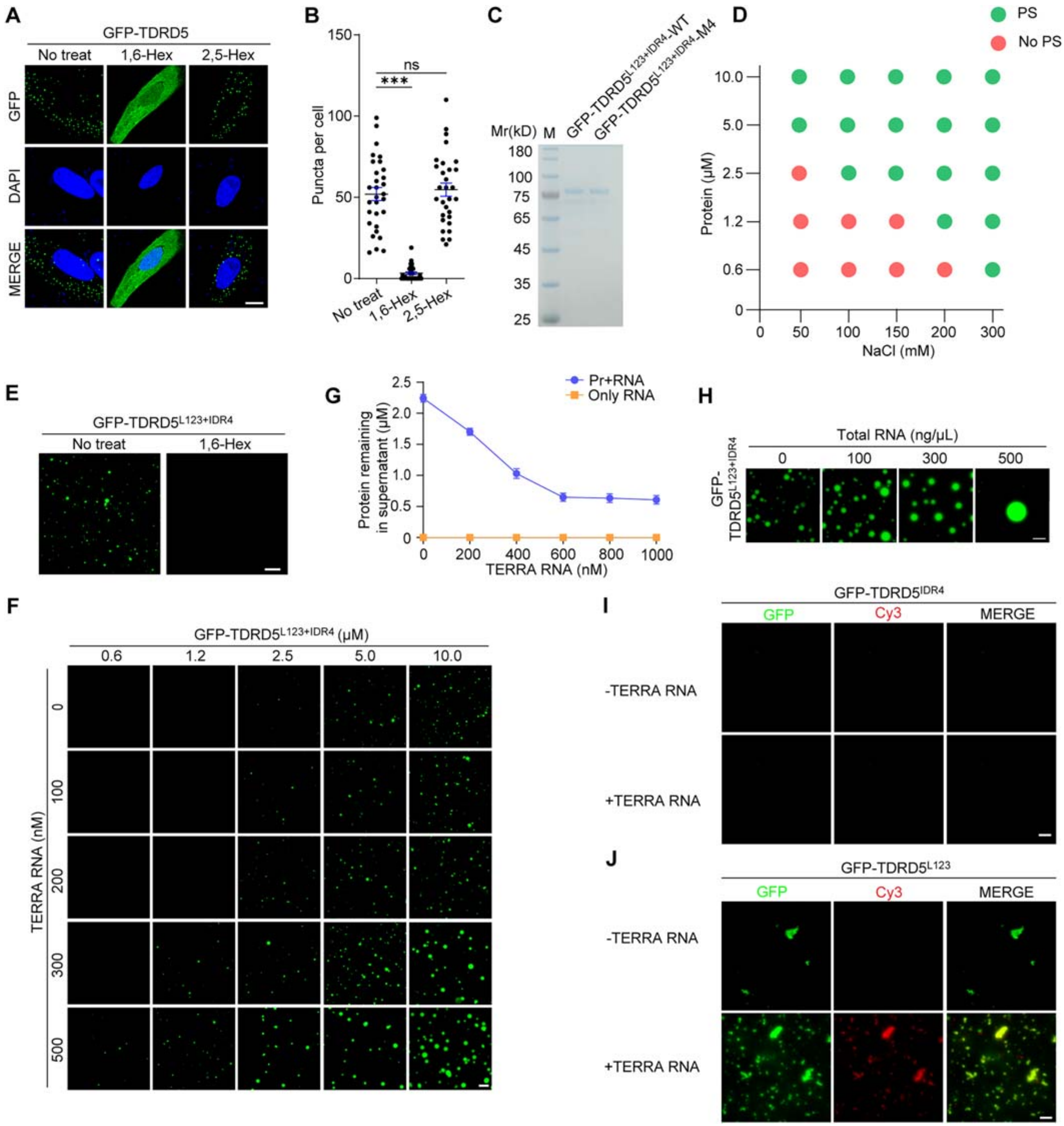

Figure EV4. TDRD5 undergoes RNA-dependent phase separation.

(**A**) Images of HeLa cells transfected with GFP-TDRD5 exposed to 1,6-hexanediol (1,6-Hex) or 2,5-hexanediol (2,5-Hex) were shown. Scale bars, 10 μm. (**B**) Quantification of the puncta number per cell were shown based on (**A**). $n = 30$ cells. ns, not significant, $P = 0.6341$; ***$P = 4.73229\text{E-}13$. P values were calculated using Welch's t test. The data represent the mean ± s.e.m. (**C**) SDS-PAGE analysis showing the purified proteins. The protein standard molecular weights are labeled. (**D**) Phase diagram of purified recombinant GFP-TDRD5$^{L123+IDR4}$ at various amounts and with increasing concentrations of NaCl. (**E**) Purified protein of GFP-TDRD5$^{L123+IDR4}$ exposed to 1,6-hexanediol were shown (5 μM Protein, 150 mM NaCl). Scale bars, 10 μm. (**F**) Phase separation of purified recombinant GFP-TDRD5$^{L123+IDR4}$ at various amounts and with increasing concentrations of TERRA RNA (150 mM NaCl). Scale bar, 10 μm. (**G**) Protein remaining in supernatant after phase separation for 2.5 μM GFP-TDRD5$^{L123+IDR4}$ for 0–1000 nM TERRA RNA with 150 mM NaCl. $n = 3$. (**H**) In vitro droplet formation of purified GFP-TDRD5$^{L123+IDR4}$ (5 μM Protein, 150 mM NaCl) at the indicated total RNA concentrations in the presence of 150 mM NaCl. Scale bars, 10 μm. (**I**) Phase separation of purified recombinant GFP-TDRD5^{IDR4} with Cy3 labeled TERRA RNA (10 μM Protein, 150 mM NaCl). Scale bars, 10 μm. (**J**) Phase separation of purified recombinant GFP-TDRD5^{L123} with Cy3 labeled TERRA RNA (10 μM Protein, 150 mM NaCl). Scale bars, 10 μm.

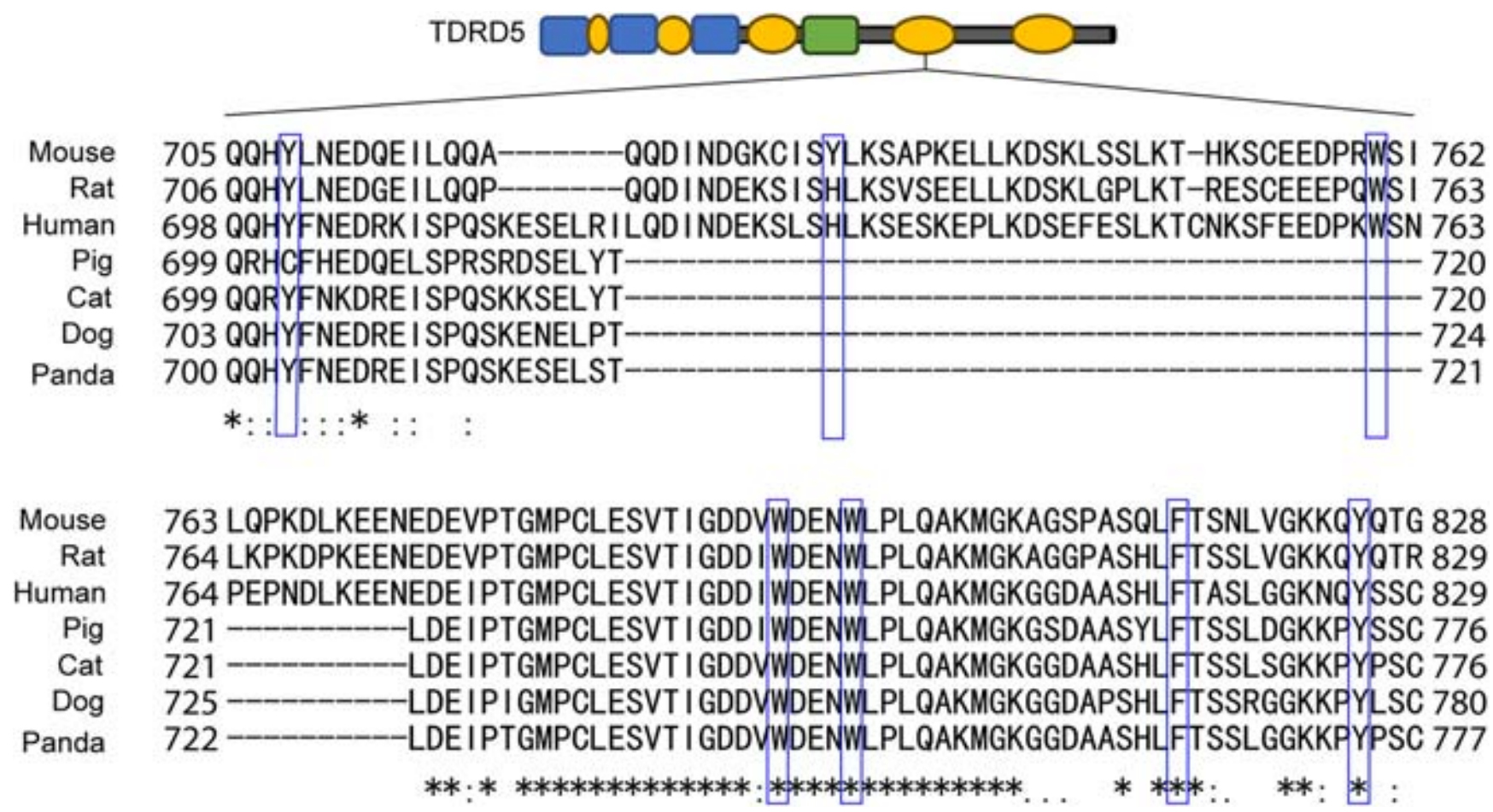

Figure EV5. Aromatic residues in IDR4 are conserved in mammals.

Multiple sequence alignment of TDRD5 IDR4 from mouse, human, rat, panda, cat, pig and dog using Clustal Omega. The aromatic amino acids were shown in blue square.

 © The Author(s)

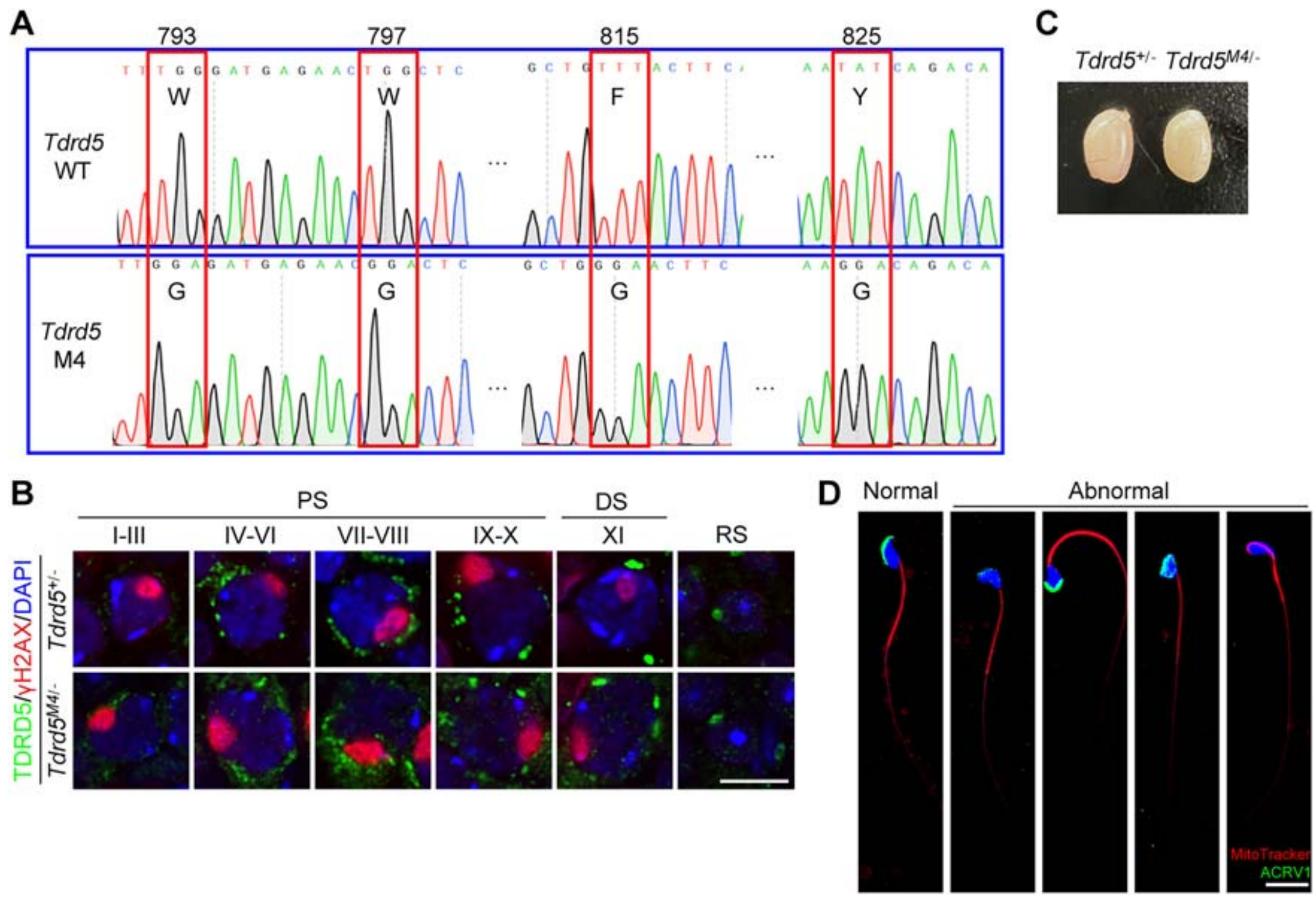

Figure EV6. TDRD5 phase separation deficiency impairs male fertility in mice.

(**A**) Sanger sequencing of genotyping PCR products amplified to confirm the *Tdrd5* M4 mutant alleles. (**B**) Co-immunostaining of TDRD5 and γH2AX on testis sections from adult *Tdrd5*$^{+/-}$ and *Tdrd5*$^{M4/-}$ mice. Different germ cell types were distinguished according to γH2AX and DAPI staining. PS, pachytene spermatocyte; DS, diplotene spermatocyte; RS, round spermatid. Scale bars, 10 μm. (**C**) Testis size of adult *Tdrd5*$^{+/-}$ and *Tdrd5*$^{M4/-}$ mice are shown. (**D**) Co-immunostaining of ACRV1 and MitoTracker on sperms from adult *Tdrd5*$^{M4/-}$ mouse epididymis. Scale bars, 10 μm.

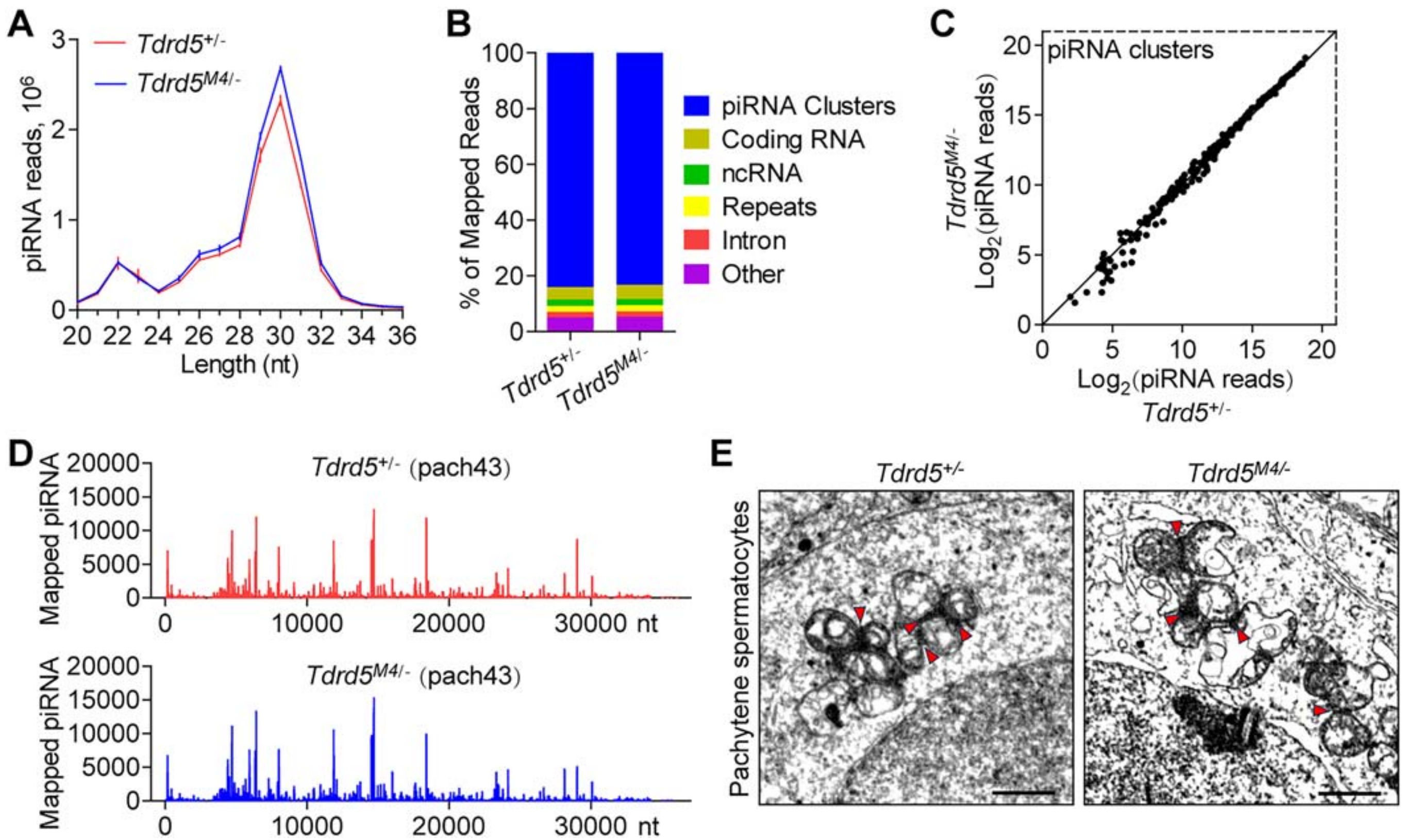

Figure EV7. TDRD5 phase separation is dispensable for pachytene piRNA biogenesis.

(**A**) The length distribution of piRNAs in adult testes. Data were normalized by miRNA reads. (**B**) Genomic annotation of total piRNA from adult testes. (**C**) Scatter plot of piRNA reads mapped to individual piRNA clusters from *Tdrd5*$^{+/-}$ and *Tdrd5*$^{M4/-}$ mice. (**D**) Distribution of piRNA reads mapping to a representative piRNA clusters (pach43) from *Tdrd5*$^{+/-}$ and *Tdrd5*$^{M4/-}$ mice. (**E**) TEM on pachytene spermatocytes from adult testes. Scale bars, 1 µm. The IMC structure is indicated by red arrow.

 © The Author(s)

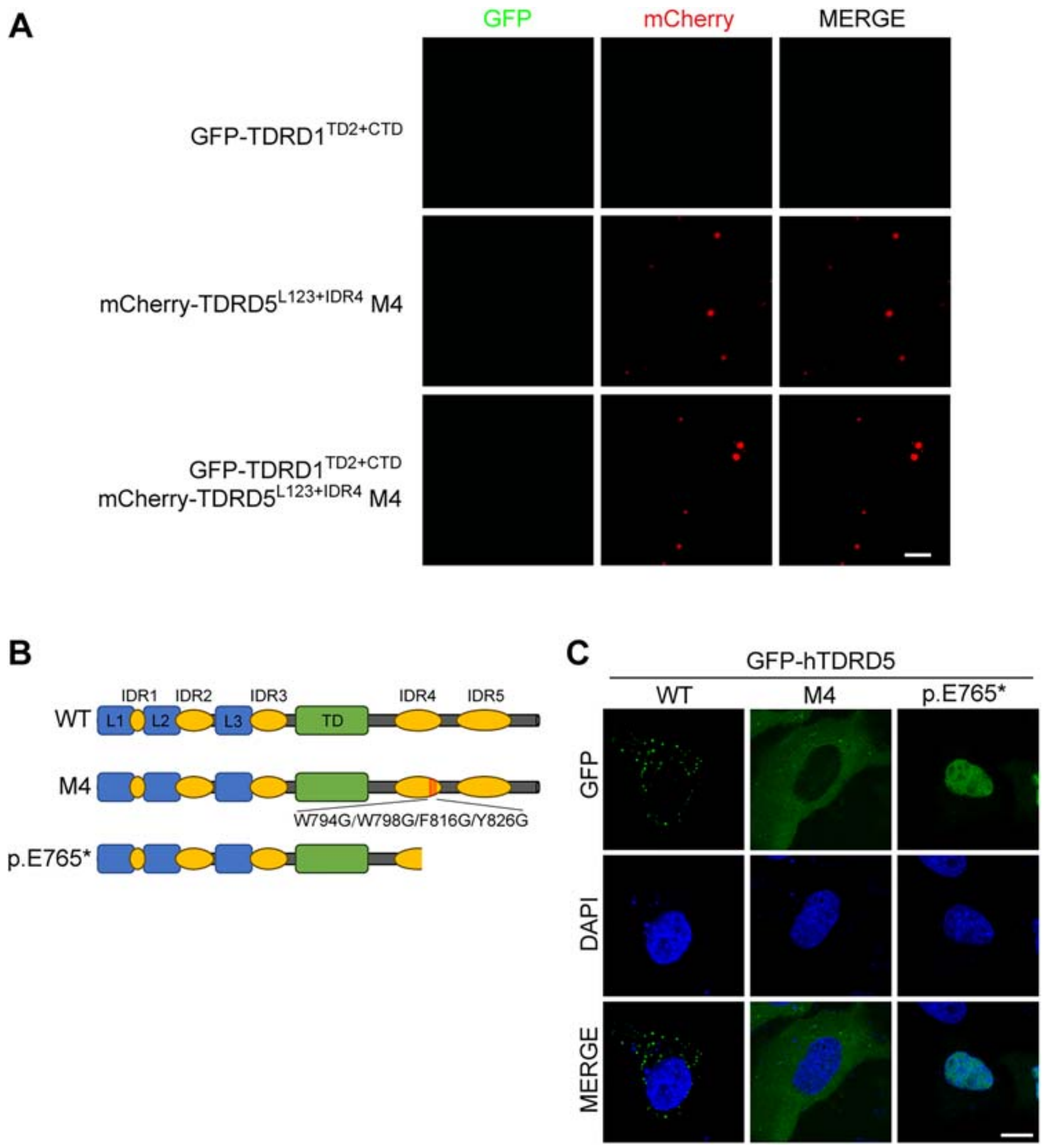

Figure EV8. TDRD5 phase separation may play roles in human male fertility.

(**A**) Droplet formation using purified recombinant mCherry-TDRD5$^{L123+IDR4}$ M4 and GFP-TDRD1$^{TD2+CTD}$ in vitro. Scale bars, 10 µm. (**B**) Schematic representation of human TDRD5 constructs. (**C**) Images of HeLa cells transfected with indicated human TDRD5 constructs were shown. Scale bars, 10 µm.
