# Supplementary material for: TDRD5 facilitates piRNA biogenesis and male fertility through phase separation
Source: EMBO Rep. 2026 Aug 4;27(18):5292–316. doi: 10.1038/s44319-026-00890-6 (PMC13601554; doi:10.1038/s44319-026-00890-6)
Supplement: Supplementary file 2 [file 44319_2026_890_MOESM2_ESM.pdf]

EMBO reports

TDRD5 facilitates piRNA biogenesis and male fertility through phase separation

Wenyang Yu, Jie Gao, Guanyi Shang, Canmei Chen, Ting Zhao, Ke Wang, Miao Li, Na jiang, and Deqiang Ding

Corresponding author(s): Deqiang Ding (dingdeqiang@tongji.edu.cn) , Miao Li (miao_li@tongji.edu.cn), Na jiang (jiangna@renji.com)

Review Timeline:

Submission Date:	17th Jan 26
Editorial Decision:	23rd Mar 26
Revision Received:	31st May 26
Editorial Decision:	6th Jul 26
Revision Received:	10th Jul 26
Accepted:	16th Jul 26

Editor: Esther Schnapp

Transaction Report:

(Note: With the exception of the correction of typographical or spelling errors that could be a source of ambiguity, letters and reports are not edited. Depending on transfer agreements, referee reports obtained elsewhere may or may not be included in this compilation. Referee reports are anonymous unless the Referee chooses to sign their reports.)

1st Editorial Decision 23rd Mar 2026

Dear Prof. Ding,

Thank you for the submission of your manuscript to EMBO reports. We have now received the full set of referee reports that is pasted below.

As you will see, the referees acknowledge that the findings are interesting. However, they also have several suggestions for how the study could be improved and the findings strengthened. I think all suggestions are good and should be addressed, but please let me know in case you disagree and we can discuss the exact revision requirements further, also in a video chat, if you like.

I would thus like to invite you to revise your manuscript with the understanding that the referee concerns must be fully addressed and their suggestions taken on board. Please address all referee concerns in a complete point-by-point response.

Acceptance of the manuscript will depend on a positive outcome of a second round of review. It is EMBO reports policy to allow a single round of major revision only and acceptance or rejection of the manuscript will therefore depend on the completeness of your responses included in the next, final version of the manuscript.

We realize that it is difficult to revise to a specific deadline. In the interest of protecting the conceptual advance provided by the work, we recommend a revision within 3 months (23rd Jun 2026). Please discuss the revision progress ahead of this time with the editor if you require more time to complete the revisions.

IMPORTANT NOTE: we perform an initial quality control of all revised manuscripts before re-review. Your manuscript will FAIL this control and the handling will be DELAYED if the following APPLIES:
1) A data availability section providing access to data deposited in public databases is missing. If you have not deposited any data, please add a sentence to the data availability section that explains that.
2) Your manuscript contains statistics and error bars based on n=2. Please use scatter blots in these cases. No statistics should be calculated if n=2.

When submitting your revised manuscript, please review the submission guidelines (https://link.springer.com/journal/44319/submission-guidelines#cms-Revised-submissions) and carefully review the instructions that follow below. Failure to include requested items will delay the evaluation of your revision.

1) a .docx formatted version of the manuscript text (including legends for main figures, EV figures and tables). Please make sure that the changes are highlighted to be clearly visible.

2) individual production quality figure files as .eps, .tif, .jpg (one file per figure). See hhttps://media.springernature.com/original/springer-cms/rest/v1/content/27825798/data/v1 for more info on how to prepare your figures.

3) We replaced Supplementary Information with Expanded View (EV) Figures and Tables that are collapsible/expandable online. A maximum of 5 EV Figures can be typeset. EV Figures should be cited as 'Figure EV1, Figure EV2" etc... in the text and their respective legends should be included in the main text after the legends of regular figures.

- For the figures that you do NOT wish to display as Expanded View figures, they should be bundled together with their legends in a single PDF file called *Appendix*, which should start with a short Table of Content. Appendix figures should be referred to in the main text as: "Appendix Figure S1, Appendix Figure S2" etc. See 'Expanded View' section of the submission guidelines.

- Additional Tables/Datasets should be labeled and referred to as Table EV1, Dataset EV1, etc. Legends have to be provided in a separate tab in case of .xls files. Alternatively, the legend can be supplied as a separate text file (README) and zipped together with the Table/Dataset file.

4) a .docx formatted letter INCLUDING the reviewers' reports and your detailed point-by-point responses to their comments. As part of the EMBO Press transparent editorial process, the point-by-point response is part of the Review Process File (RPF), which will be published alongside your paper.

5) a complete author checklist, which you can download from our author guidelines. Please insert information in the checklist that is also reflected in the manuscript. The completed author checklist will also be part of the RPF.

6) Please note that all corresponding authors are required to supply an ORCID ID for their name upon submission of a revised manuscript (<https://orcid.org/>), which can be linked to your profile in the manuscript submission system.

7) Before submitting your revision, primary datasets produced in this study need to be deposited in an appropriate public

database. Please remember to provide a reviewer password if the datasets are not yet public. The accession numbers and database should be listed in a formal "Data Availability" section placed after Methods. Please note that the Data Availability Section is restricted to new primary data that are part of this study. * Note - All links should resolve to a page where the data can be accessed. *

If your study has not produced novel datasets, please mention this fact in the Data Availability Section.

8) At EMBO Press we ask authors to provide source data for the main manuscript figures. You will receive a separate email with instructions for providing source data with your revised manuscript, including how to upload and organize the files.

9) Our journal also encourages inclusion of *data citations in the reference list* to directly cite datasets that were re-used and obtained from public databases. Data citations in the article text are distinct from normal bibliographical citations and should directly link to the database records from which the data can be accessed. In the main text, data citations are formatted as follows: "Data ref: Smith et al, 2001" or "Data ref: NCBI Sequence Read Archive PRJNA342805, 2017". In the Reference list, data citations must be labeled with "[DATASET]". A data reference must provide the database name, accession number/identifiers and a resolvable link to the landing page from which the data can be accessed at the end of the reference.

10) Regarding data quantification, the following points must be specified in each figure legend:

- the name of the statistical test used to generate error bars and P values,

- the number (n) of independent experiments (please specify technical or biological replicates) underlying each data point,

- the nature of the bars and error bars (s.d., s.e.m.),

- If the data are obtained from n Program fragment delivered error ``Can't locate object method "less" via package "than" (perhaps you forgot to load "than"?) at //ejpvfs23/sites23b/embor_www/letters/embor_decision_revise_and_review.txt line 54.' 2, use scatter blots showing the individual data points.

Discussion of statistical methodology can be reported in the materials and methods section, but figure legends should contain a basic description of n, P and the test applied.

- Please also include scale bars in all microscopy images.

11) The journal requires a statement specifying whether or not authors have competing interests (defined as all potential or actual interests that could be perceived to influence the presentation or interpretation of an article). In case of competing interests, this must be specified in your disclosure statement.

12) All Materials and Methods need to be described in the main text using our 'Structured Methods' format, which is required for all research articles. According to this format, the Methods section includes a separate Reagents and Tools Table file (listing key reagents, experimental models, software and relevant equipment and including their sources and relevant identifiers) and a Methods and Protocols section describing the methods using a step-by-step protocol format. The aim is to facilitate adoption of the methodologies across labs. More information on how to adhere to this format as well as a downloadable template (.docx) for the Reagents and Tools Table can be found in our author guidelines.

We would also welcome the submission of cover suggestions, or motifs to be used by our Graphics Illustrator in designing a cover.

As part of the EMBO publication's Transparent Editorial Process, EMBO reports publishes online a Review Process File (RPF) to accompany accepted manuscripts. This File will be published in conjunction with your paper and will include the referee reports, your point-by-point response and all pertinent correspondence relating to the manuscript.

You are able to opt out of this by letting the editorial office know (emboreports@embo.org). If you do opt out, the Review Process File link will point to the following statement: "No Review Process File is available with this article, as the authors have chosen not to make the review process public in this case."

I look forward to seeing a revised form of your manuscript when it is ready.

Best regards,
Esther

Esther Schnapp, PhD
Senior Editor

Referee #1:

In this manuscript, the authors investigate the molecular mechanism of TDRD5 phase separation activity and its physiological role in piRNA biogenesis and spermatogenesis. They began by screening known piRNA-associated proteins for phase separation activity and observed that TDRD1, TDRD5, and TDRD4 form prominent condensates. Focusing on TDRD5, the authors first demonstrated its ability to undergo phase separation. This activity is mediated by RNA binding through the LOTUS domain and multivalent interactions involving its intrinsically disordered region (IDR). Furthermore, using phase separation-deficient mice, the authors showed that TDRD5 phase separation activity is functionally important for piRNA production in gonocytes but appears less critical for pachytene piRNA biogenesis. Overall, the work presents solid and reliable data, lending high credibility to the findings. It provides valuable insights into piRNA generation and germ granule assembly. I have a few suggestions for further improvement before publication。

Major Comments

1.The authors show that TDRD1 and TDRD5 colocalize and enhance each other's phase separation in vitro (Fig. 7H), yet their physical interaction in Co-IP is "minimal" (Fig. 7F). This suggests the interaction might be purely biophysical (co-condensation) rather than a rigid protein-protein interaction. The authors should discuss this more clearly.

2.In Figure 1A, the authors expressed 25 GFP-tagged piRNA-related proteins in HeLa cells to assess their phase separation potential. However, unlike in subsequent figures, no quantitative assessment of condensate numbers per cell was provided. It is recommended that the authors include a bar chart summarizing these counts, which would allow for a more intuitive and comparative presentation of the screening results.

3.Although Figure 7 presents fluorescence colocalization images, visual inspection alone may not be conclusive for some panels. To strengthen the validity of the colocalization claims, the authors should perform quantitative analyses-such as calculating Pearson's correlation coefficients (PCC) or generating line-scan intensity profiles-to provide robust statistical evidence of spatial overlap.

Minor Comments

1.page 2 line29-30: the phrase "TDRD5 phase separation deficiency impaired male infertility" is grammatically confusing (impairing infertility
implies improving fertility). It should likely be "caused male infertility" or "impaired male fertility."

2.Page 13 line 292: "undergoes phase separation and paly roles in IMC assembly" should be "plays"

3.Page 16 line 413: "imaged for doplet formation" should be " droplet"

4.Page 15 line 380: " FKBP6, HSP90awere amplified by PCR" should be add " HSP90a were "

5.Page 3 line 60: "there are two distinct piRNA production: fetal piRNAs" should be "two distinct modes of piRNA production"

Referee #2:

In their manuscript, Yu et al. screen 25 piRNA-pathway factors for their ability to form condensates in HeLa cells and report that, besides the known scaffold TDRD1, TDRD5 and RNF17/TDRD4 form cytoplasmic granules upon overexpression. They then focus on TDRD5 and demonstrate that it forms dynamic, liquid-like condensates in cell lines and in vitro using a recombinant fragment. TDRD5 condensation is driven cooperatively by its N-terminal tandem LOTUS domains and a specific intrinsically disordered region (IDR4), whereas the Tudor domain is dispensable for droplet formation. The authors further show that phase separation is strongly enhanced by G-quadruplex-forming TERRA RNA and testis total RNA, and that mutating aromatic residues within IDR4 strongly impairs condensate formation in cells and in vitro. Using TDRD5 M4 mutant and knockout mice, the authors provide in vivo evidence that TDRD5 condensation is required for proper IMC assembly and fetal piRNA production in neonatal gonocytes. Interestingly, they find that pachytene piRNA biogenesis and IMC morphology in pachytene spermatocytes are only mildly affected, whereas adult males exhibit relatively preserved spermatogenesis but reduced sperm quality and fertility. Finally, the authors show that TDRD1 and TDRD5 colocalize in IMCs, mutually enhance condensate formation, and that TDRD5 is recruited to the IMC via interaction with PIWIL2.
Overall, this work advances the concept that specific phase-separating scaffolds contribute in a stage-specific and mechanistically distinct manner to germ granule organization and piRNA biogenesis. It also shows potential implications for understanding certain forms of human male infertility due to their conserved residues involved in phase separation. This manuscript is a strong fit for EMBO Reports, and I have minor and a few major suggestions.

Minor:

-In Figure 1A, the authors show the only 24 overexpress proteins in HeLa cells, while the text says 25. From those, only three

proteins can phase separate. However, since phase separation is highly dependent on protein concentration, it would be informative to show the relative expression levels of those 24 proteins. Quantification from the micrographs might be sufficient.
-In line 105, it would be informative to explicitly state the reasoning behind the choice of those 25/4? proteins, to better target a broader audience.
-In line 107, RNF17/TDRD4 is identified as a phase-separating protein, but there is no further data or discussion about it. It would be important to explain why it was not used for further characterization.
-In lines 137-139, it is mentioned that other IDR deletions had minimal effects on phase separation. It would be important to add statistics to support a more objective interpretation. This comment applies more generally to all quantification plots without statistics, for example (but not limited to) Figures 2D, 2H, 2K, 4C, and 4E.
-In lines 155-156, it says: "This treatment significantly reduced TDRD5 granules, whereas the control reagent 2,5-hexanediol did not (Figs. EV2A and 3B)." However, the cited figure appears incorrect. This should likely read "Figs. EV2A, B" rather than 3B.
-In lines 181-182, it states: "Purified TDRD5L123+IDR4 M4 protein failed to undergo phase separation in vitro (Fig. 4F)," but the panel shows condensates in the M4 mutant at 10 μM. This sentence needs to be rephrased to reflect the data.
-In lines 186-188, it states: "To assess the physiological role of TDRD5 phase separation, we used CRISPR-Cas9 to generate mice carrying the M4 mutation (Tdrd5M4) and Tdrd5 knockout (Tdrd5−/−) mice (Fig. EV3A)." However, only the M4 mutation (Tdrd5M4) together with WT is shown. This description should be corrected.
-In lines 188-190, it is mentioned that Tdrd5 M4 does not affect TDRD5 protein stability, as shown in Figures 5A and EV3B. These data should be quantified to support that claim. In addition, the western blot shows two bands: is this due to antibody cross-reactivity or another TDRD5 isoform? A brief explanation would be helpful. Including the TDRD5 knockout sample in the western blot would also clarify this point.
-In lines 195-196, it is mentioned that morphological analysis revealed that Tdrd5M4/− mice exhibited more abnormal sperm. It would be helpful to briefly describe the criteria used to define "abnormal" morphologies in Figure EV3D for a general audience.
-In lines 200-212 and Figure EV4, the human data section feels somewhat out of place in the flow of the Results. I recommend moving this part toward the end of the Results.
-In most panels of Figure 6, data from both the M4 mutant and knockout are shown, except for 6G and 6H. It would be useful to also include knockout data in those panels. The same comment applies to Figure 5 and Figure EV5.
-In line 219, the genotype name "drd5M4/−" is miswritten and should be corrected to "Tdrd5M4/−".

Major:
-It would be interesting to determine whether the LOTUS domains or IDR4 alone can form condensates in vitro. It would also be important to test whether G-rich RNA (G-quadruplex RNA TERRA) enhances phase separation in these minimal constructs.
-For Figure 3A, since the authors already have the data, it would be valuable to present a phase diagram to summarize the salt- and concentration-dependent phase behavior.
-For Figure 3E, to make the conclusion more quantitative, the authors could estimate the saturation concentration (Csat) to quantify the effect of RNA on phase separation. Maybe you can use FCS or centrifugation-based assays for this purpose. It would also strengthen the argument to include a control panel where only RNA is added as control.
-For Figure 7E-H, the in vivo and in vitro data appear contradictory. While TDRD1 and TDRD5 do not interact strongly or colocalize in HeLa cells, TDRD1 is nonetheless recruited into TDRD5 condensates. One would expect that using the TDRD1 3GS mutant together with TDRD5 WT, which forms condensates, would be sufficient to recruit TDRD1 into these condensates, and vice versa. Perhaps the authors could add RNA to the system and test whether this better recapitulates the in vivo results. Another explanation is that the same amino acids responsible for phase separation, e.g. M4, of each protein are also responsible for their mixing behavior. In that context, it would be helpful to perform the same assay as in Figure 7H with the TDRD5 M4 mutant (at concentrations above 10 μM, where it still shows condensation).

Referee #3:

piRNAs, which suppress transposon expression, are produced in the intermitochondrial cement (IMC), a germ granule that assembles through phase separation. Previous work showed that TDRD1 undergoes phase separation to promote IMC formation. In this study, the authors demonstrate that TDRD5 also undergoes phase separation and contributes to IMC organization.

They show that TDRD5 phase separation requires cooperative action of its tandem Lotus domains and IDR4, with aromatic residues in IDR4 playing a critical role. Interaction with PIWIL2 (MILI) is required for recruitment of TDRD5 to the IMC. Notably, loss of TDRD5 phase separation significantly reduces fetal piRNA abundance in gonocytes, whereas pachytene piRNA biogenesis is largely unaffected, suggesting stage-specific roles.

Furthermore, RNA enhances TDRD5 phase separation in vitro, consistent with the reported binding of Lotus domains to G-quadruplex (G4) RNA. The mechanism identified here differs from that reported for TDRD1, highlighting mechanistic diversity in IMC assembly. Overall, the study provides physiologically relevant evidence linking phase separation to piRNA production and male fertility. While some mechanistic aspects require further clarification, the work advances our understanding of how phase separation contributes to stage-specific regulation of the piRNA pathway.

Major concerns

1, Line 131: The optoDroplet assay indicates that the second and third Lotus domains (L2 and L3) possess phase separation capacity. However, it remains unclear whether deletion of L2 and L3 abolishes condensation to the same extent as deletion of all three Lotus domains. A direct comparison of these mutants would clarify the relative contribution of each Lotus domain and determine whether L2 and L3 act as dominant drivers of TDRD5 condensation.

2, L133: In contrast to L2 and L3, the first Lotus domain (L1) does not exhibit phase separation capacity in the optoDroplet assay (Fig. 2E). Given that the authors provide a multi-species conservation analysis for IDR4 (Appendix Fig. S2), a similar comparative analysis of L1 versus L2/L3 (e.g., sequence alignment and conservation profiling across species) would help clarify whether specific conserved residues or multivalent features correlate with the distinct condensation behavior. Such analysis could strengthen the mechanistic interpretation of functional divergence among the Lotus domains.

3, Line 141: The authors show that L123+IDR4 forms pronounced condensates (Figs. 2J and K). However, the construct containing L123 without IDR4 was not presented. This control would be necessary to demonstrate functional cooperativity between the Lotus domains and IDR4. In addition, single Lotus domain + IDR4 constructs localize to both cytoplasm and nucleus, whereas L123+IDR4 condensates appear restricted to the nucleus. What accounts for this difference in subcellular localization? Is nuclear enrichment required for efficient condensation?

4, Line 166: Addition of Cy3-labeled TERRA RNA enhances droplet formation in vitro (Fig. 3E), particularly at 200-500 nM RNA. Is there biochemical evidence that TDRD5 binds RNA within this concentration range? Does IDR4 contribute to RNA-mediated enhancement, or is the effect entirely dependent on the Lotus domains? Further clarification would help define the molecular basis of RNA-dependent phase separation.

5, L257: The authors conclude that deletion of the N-terminal region or PIWI domain of PIWIL2 impairs interaction with TDRD5 (Fig. 7C). However, the observed differences appear modest. Quantitative analysis (e.g., band intensity normalization from multiple independent experiments) would strengthen this conclusion.

Minor concerns

1, L105: The manuscript states that 25 GFP-tagged piRNA-related proteins were constructed; however, Figure 1A appears to show 24 proteins. Please clarify this discrepancy.

2, L75-87 and L147: A previous study on the Drosophila TDRD5 homolog Tejas (J. Cell Biol., 2023) demonstrated that its IDR contributes to granule assembly. This work should be cited and briefly discussed in the Introduction, and its relevance acknowledged at L147, to appropriately position the present findings within an evolutionary context.

3, L156: The text refers to "Figs. EV2A and 3B," but this appears to be incorrect. It should likely read "Figs. EV2A and 2B." Please verify.

4, L218: The sentence states that the IMC defect was less severe in "drd5M4/- and Tdrd5-/-" gonocytes. This appears to be a typographical error and should read "less severe in Tdrd5M4/- than in Tdrd5-/- gonocytes."

5, L292: "paly" should be corrected to "play."

6, Fig. EV1C: Purified GFP-TDRD5L123+IDR4 (WT and M4 mutant) appear to migrate slightly differently despite having identical predicted lengths. Please clarify whether this reflects conformational differences, partial degradation, or tag processing efficiency.

7, L139: The optoDroplet assay shows that IDR4 robustly forms light-induced condensates (Fig. 2I). However, IDR1 also appears to form nuclear condensates. Does IDR1 contain a nuclear localization signal? Clarification would help determine whether IDR1 has a distinct nuclear-specific role or whether its condensation is incidental.

Below is the point-by-point response to comments from the reviewers.

Reviewer #1:

In this manuscript, the authors investigate the molecular mechanism of TDRD5 phase separation activity and its physiological role in piRNA biogenesis and spermatogenesis. They began by screening known piRNA-associated proteins for phase separation activity and observed that TDRD1, TDRD5, and TDRD4 form prominent condensates. Focusing on TDRD5, the authors first demonstrated its ability to undergo phase separation. This activity is mediated by RNA binding through the LOTUS domain and multivalent interactions involving its intrinsically disordered region (IDR). Furthermore, using phase separation-deficient mice, the authors showed that TDRD5 phase separation activity is functionally important for piRNA production in gonocytes but appears less critical for pachytene piRNA biogenesis. Overall, the work presents solid and reliable data, lending high credibility to the findings. It provides valuable insights into piRNA generation and germ granule assembly. I have a few suggestions for further improvement before publication.

We sincerely appreciate the reviewer's positive feedback and valuable recognition of our work. We are also very grateful for the constructive suggestions provided. We have carefully considered all comments and revised the manuscript accordingly.

Major Comments

1. The authors show that TDRD1 and TDRD5 colocalize and enhance each other's phase separation in vitro (Fig. 7H), yet their physical interaction in Co-IP is "minimal" (Fig. 7F). This suggests the interaction might be purely biophysical (co-condensation) rather than a rigid protein-protein interaction. The authors should discuss this more clearly.

Response: We thank the reviewer for this comment. We agree that the apparent discrepancy between the minimal interaction observed in the Co-IP assay (Fig. 7F) and the strong colocalization and mutual enhancement of phase separation *in vitro* (Fig. 7H) is noteworthy. These results are consistent with a model in which TDRD1 and TDRD5 do not form a stable, high-affinity complex, but instead undergo biophysical co-condensation driven by multivalent and weak interactions. Such interactions are often transient and dynamic, and therefore may not be efficiently captured by conventional Co-IP assays, which typically favor stable protein-protein interactions. In contrast, phase separation assays can reveal cooperative assembly mediated by low-affinity, multivalent contacts that promote condensate formation without requiring a rigid

binding interface. We have now revised the manuscript to clarify this point (Discussion section), emphasizing that the enhancement of phase separation likely reflects cooperative partitioning into a shared condensate phase, rather than a direct, stable interaction between TDRD1 and TDRD5. We also note that this behavior is consistent with emerging models of biomolecular condensates, where components can co-localize and functionally interact through collective physicochemical properties rather than defined stoichiometric complexes.

2. In Figure 1A, the authors expressed 25 GFP-tagged piRNA-related proteins in HeLa cells to assess their phase separation potential. However, unlike in subsequent figures, no quantitative assessment of condensate numbers per cell was provided. It is recommended that the authors include a bar chart summarizing these counts, which would allow for a more intuitive and comparative presentation of the screening results.

Response: We thank the reviewer for this helpful suggestion. Based on the reviewer's suggestions, we quantified the number of puncta formed by 20 piRNA pathway proteins in cells. During the quantification process, we excluded the mitochondrial-localized proteins, including ASZ1, GPAT2, mitoPLD, and TDRKH. According to the quantification results, only TDRD1, TDRD5, and RNF17 exhibited a significant ability to form puncta. The corresponding quantitative results are provided in the revised Appendix Fig. S1A.

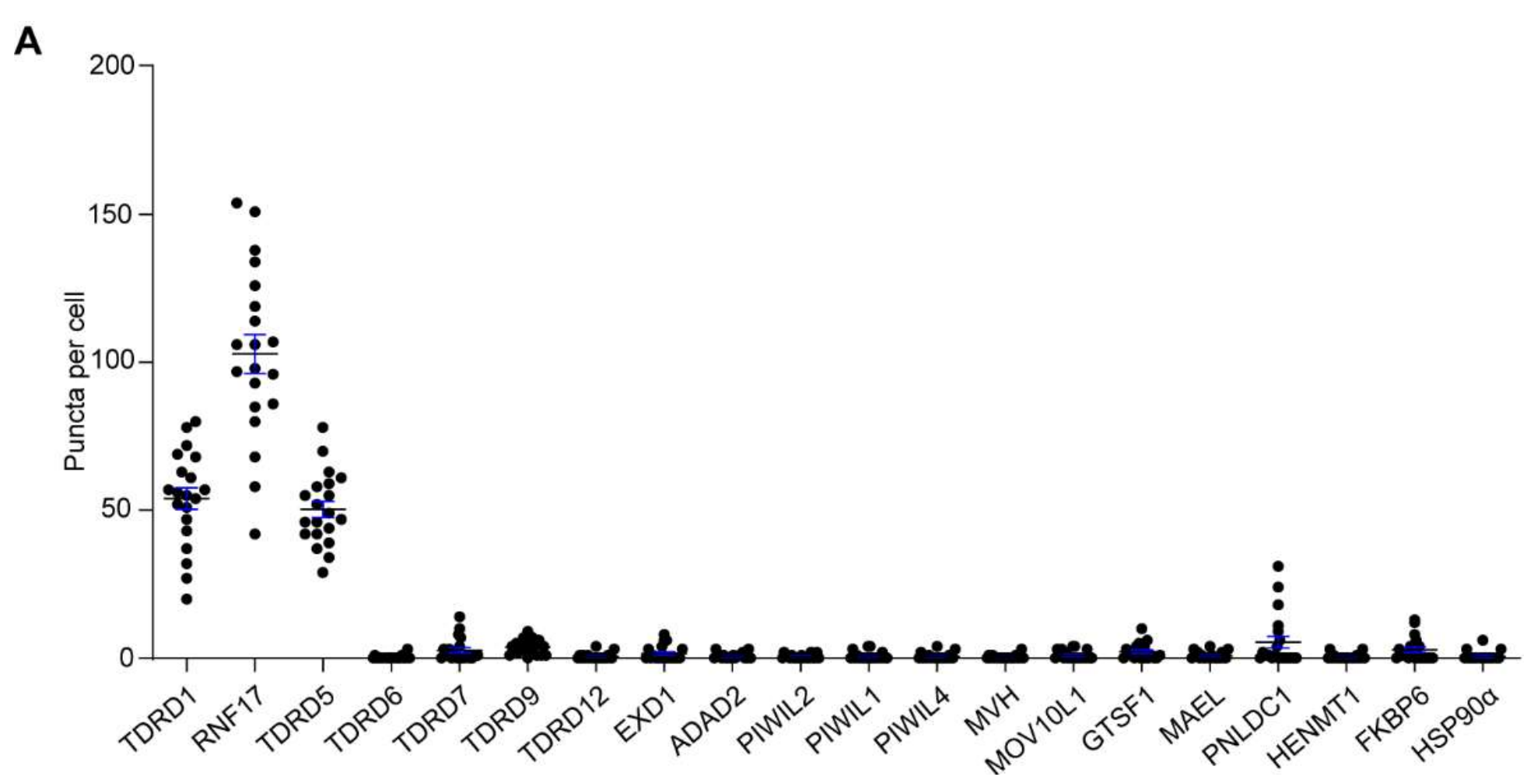

Appendix Fig. S1A

3. Although Figure 7 presents fluorescence colocalization images, visual inspection alone may not be conclusive for some panels. To strengthen the validity of the colocalization claims, the authors should perform quantitative analyses-such as calculating Pearson's correlation coefficients (PCC) or generating line-scan intensity

profiles-to provide robust statistical evidence of spatial overlap.

Response: We thank the reviewer for this important suggestion. To strengthen the colocalization conclusions beyond visual inspection, we have now performed quantitative colocalization analysis for the fluorescence channels shown in Figure 7D. The quantitative results have been added to the revised Fig. 7D.

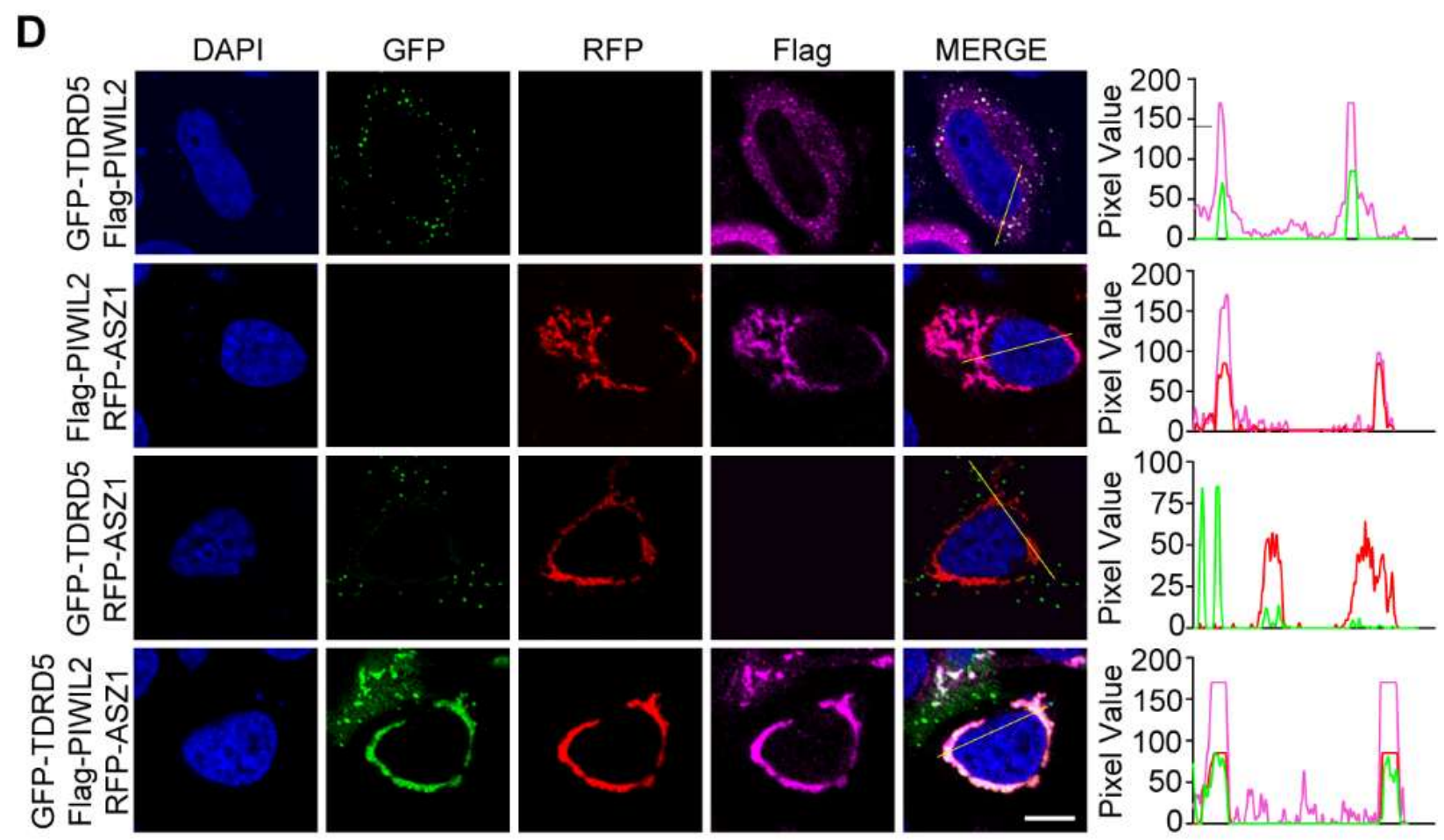

Fig. 7D

Minor Comments

1.page 2 line29-30: the phrase "TDRD5 phase separation deficiency impaired male infertility" is grammatically confusing (impairing infertility implies improving fertility). It should likely be "caused male infertility" or "impaired male fertility."

Response: We thank the reviewer for this valuable suggestion. We have changed “impaired male infertility” to “impaired male fertility”.

2.Page 13 line 292: "undergoes phase separation and paly roles in IMC assembly" should be "plays"

Response: We thank the reviewer for pointing out this mistake. We have changed “paly” to “plays”.

3.Page 16 line 413: "imaged for doplet formation" should be " droplet"

Response: We thank the reviewer for pointing out this mistake. We have corrected it in the revised manuscript.

4.Page 15 line 380: " FKBP6, HSP90awere amplified by PCR" should be add " HSP90a were "

Response: We thank the reviewer for pointing out this mistake. We have corrected it in the revised manuscript.

5.Page 3 line 60: "there are two distinct piRNA production: fetal piRNAs" should be "two distinct modes of piRNA production"

Response: We thank the reviewer for this valuable suggestion. We have changed “distinct piRNA production” to “two distinct modes of piRNA production”.

Reviewer #2:

In their manuscript, Yu et al. screen 25 piRNA-pathway factors for their ability to form condensates in HeLa cells and report that, besides the known scaffold TDRD1, TDRD5 and RNF17/TDRD4 form cytoplasmic granules upon overexpression. They then focus on TDRD5 and demonstrate that it forms dynamic, liquid-like condensates in cell lines and in vitro using a recombinant fragment. TDRD5 condensation is driven cooperatively by its N-terminal tandem LOTUS domains and a specific intrinsically disordered region (IDR4), whereas the Tudor domain is dispensable for droplet formation. The authors further show that phase separation is strongly enhanced by G-quadruplex-forming TERRA RNA and testis total RNA, and that mutating aromatic residues within IDR4 strongly impairs condensate formation in cells and in vitro. Using TDRD5 M4 mutant and knockout mice, the authors provide in vivo evidence that TDRD5 condensation is required for proper IMC assembly and fetal piRNA production in neonatal gonocytes. Interestingly, they find that pachytene piRNA biogenesis and IMC morphology in pachytene spermatocytes are only mildly affected, whereas adult males exhibit relatively preserved spermatogenesis but reduced sperm quality and fertility. Finally, the authors show that TDRD1 and TDRD5 colocalize in IMCs, mutually enhance condensate formation, and that TDRD5 is recruited to the IMC via interaction with PIWIL2.

Overall, this work advances the concept that specific phase-separating scaffolds contribute in a stage-specific and mechanistically distinct manner to germ granule organization and piRNA biogenesis. It also shows potential implications for understanding certain forms of human male infertility due to their conserved residues involved in phase separation. This manuscript is a strong fit for EMBO Reports, and I have minor and a few major suggestions.

We sincerely appreciate the reviewer’s positive feedback and valuable recognition of our work. We are also very grateful for the constructive suggestions provided. We have carefully considered all comments and revised the manuscript accordingly.

Minor:

-In Figure 1A, the authors show the only 24 overexpress proteins in HeLa cells, while

the text says 25. From those, only three proteins can phase separate. However, since phase separation is highly dependent on protein concentration, it would be informative to show the relative expression levels of those 24 proteins. Quantification from the micrographs might be sufficient.

Response: We thank the reviewer for this insightful comment. We apologize for the inconsistency between the text and Figure 1A, and we have corrected the number to 24 proteins in the revised manuscript. As suggested, we quantified the relative expression levels of the 24 GFP-tagged proteins using fluorescence intensity measurements from the micrographs. This analysis showed that the expression levels among these 24 proteins were overall comparable. The corresponding results have been added to the revised Appendix Fig. S1B.

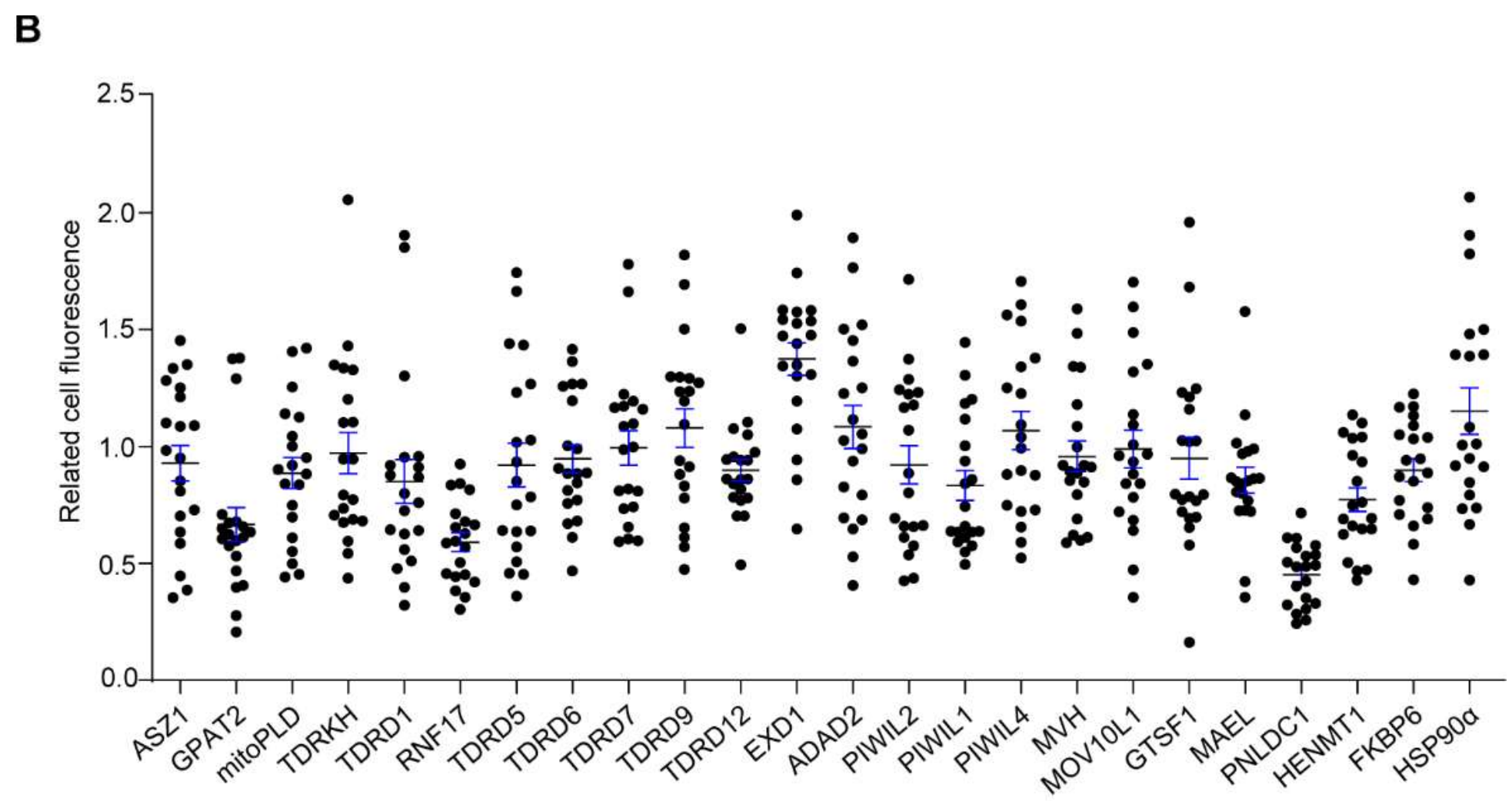

Appendix Fig. S1B

-In line 105, it would be informative to explicitly state the reasoning behind the choice of those 25/4? proteins, to better target a broader audience.

Response: We thank the reviewer for this excellent suggestion. The logic behind choosing these 24 specific proteins is that they represent the core, well-established protein components currently known to be essential for the piRNA pathway. By focusing on this comprehensively defined set, we aimed to systematically investigate the phase separation potential within the piRNA processing machinery. We have added this reasoning to the revised manuscript for general readers.

-In line 107, RNF17/TDRD4 is identified as a phase-separating protein, but there is no further data or discussion about it. It would be important to explain why it was not used for further characterization.

Response: We thank the reviewer for this comment. TDRD5 has been reported to localize primarily to the IMC, where it participates in pachytene piRNA biogenesis. RNF17 is mainly located in P-bodies. Although loss of RNF17 has been reported to cause aberrant activation of the secondary piRNA pathway, RNF17 does not substantially affect total piRNA production. This work focuses on the assembly mechanism of the IMC, a germ granule that hosts the piRNA processing complex; therefore, we primarily focused on TDRD5. Nevertheless, RNF17 also exhibits phase separation potential, and we plan to investigate its phase separation ability and physiological functions in future work. To clarify this for the readers, we have now added a brief explanation in the revised manuscript regarding our rationale for prioritizing TDRD5 over RNF17 in this manuscript.

-In lines 137-139, it is mentioned that other IDR deletions had minimal effects on phase separation. It would be important to add statistics to support a more objective interpretation. This comment applies more generally to all quantification plots without statistics, for example (but not limited to) Figures 2D, 2H, 2K, 4C, and 4E.

Response: We thank the reviewer for this important suggestion. We agree that statistical analyses are necessary to support an objective interpretation of the effects of IDR deletions on phase separation and, more broadly, for all quantification plots. Following the reviewer's suggestion, we have carefully reviewed all quantification plots in the manuscript and performed the appropriate statistical tests (Fig. 2D, 2H, 2K, 4C, 4E and EV2B). We revealed that the deletion of IDR1, IDR2, or IDR3 mildly impaired TDRD5 droplet formation, whereas loss of IDR4 completely disrupted it. We have revised this in the manuscript to clarify this result more accurately.

-In lines 155-156, it says: "This treatment significantly reduced TDRD5 granules, whereas the control reagent 2,5-hexanediol did not (Figs. EV2A and 3B)." However, the cited figure appears incorrect. This should likely read "Figs. EV2A, B" rather than 3B.

Response: We thank the reviewer for pointing out this mistake. We have corrected it in the revised manuscript.

-In lines 181-182, it states: "Purified TDRD5$^{L123+IDR4}$ M4 protein failed to undergo phase separation in vitro (Fig. 4F)," but the panel shows condensates in the M4 mutant at 10 μM. This sentence needs to be rephrased to reflect the data.

Response: We thank the reviewer for this comment. We have now revised the sentence to clarify that the M4 mutant shows reduced or weakened phase separation capacity, rather than a complete loss of phase separation. The revised sentence is as follows:

"Purified TDRD5$^{L123+IDR4}$ M4 protein exhibited markedly reduced phase separation and only formed condensates at high concentrations (10 μM), indicating impaired phase separation capacity."

-In lines 186-188, it states: "To assess the physiological role of TDRD5 phase separation, we used CRISPR-Cas9 to generate mice carrying the M4 mutation (Tdrd5M4) and Tdrd5 knockout (Tdrd5−/−) mice (Fig. EV3A)." However, only the M4 mutation (Tdrd5M4) together with WT is shown. This description should be corrected.

Response: We thank the reviewer for this comment. We have corrected it in the revised manuscript.

-In lines 188-190, it is mentioned that Tdrd5 M4 does not affect TDRD5 protein stability, as shown in Figures 5A and EV3B. These data should be quantified to support that claim. In addition, the western blot shows two bands: is this due to antibody cross-reactivity or another TDRD5 isoform? A brief explanation would be helpful. Including the TDRD5 knockout sample in the western blot would also clarify this point.

Response: We thank the reviewer for this helpful suggestion. To support the statement that the Tdrd5 M4 mutation does not affect TDRD5 protein stability, we have now performed densitometric quantification of the western blots in Figures 5A. As suggested, we included a TDRD5 knockout (KO) control in the western blot, which confirms the specificity of the TDRD5 antibody. The results were showed in the revised Fig. 5A.

Regarding the two observed TDRD5 bands, two isoforms of TDRD5 are expressed in mouse testes: isoform 1 represents the longest TDRD5, which contains all three Lotus domains, and isoform 2 represents a shorter TDRD5 that lacks Lotus2(Ding *et al*, 2018; Yabuta *et al*, 2011). We have added a brief explanation of this point to the revised manuscript for clarity.

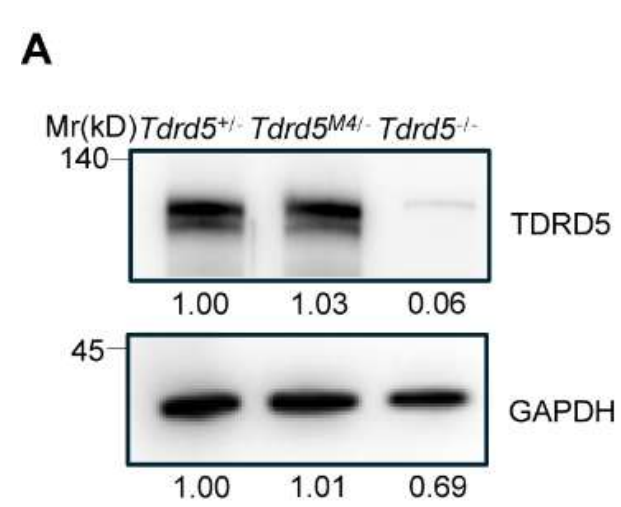

Fig. 5A

-In lines 195-196, it is mentioned that morphological analysis revealed that Tdrd5M4/− mice exhibited more abnormal sperm. It would be helpful to briefly describe the criteria used to define "abnormal" morphologies in Figure EV3D for a general audience.

Response: We thank the reviewer for this helpful suggestion. In the revised manuscript,

we have now included a brief description of the criteria used to classify abnormal sperm.

-In lines 200-212 and Figure EV4, the human data section feels somewhat out of place in the flow of the Results. I recommend moving this part toward the end of the Results.

Response: We thank the reviewer for this constructive suggestion. We have relocated this entire section and the corresponding extended data to the very end of the Results section. We believe this structural adjustment significantly improves the overall readability and narrative flow of the manuscript.

-In most panels of Figure 6, data from both the M4 mutant and knockout are shown, except for 6G and 6H. It would be useful to also include knockout data in those panels. The same comment applies to Figure 5 and Figure EV5.

Response: We thank the reviewer for this helpful suggestion. Accordingly, we have now included the *Tdrd5* KO data in the revised Fig. 6G and 6H, as well as in the revised Fig. 5A-D. Fig. 5E-H does not contain *Tdrd5* KO data, because no mature sperm were observed in the *Tdrd5* KO epididymis. In addition, *Tdrd5* KO may lead to defect of spermatocyte in adult testes and fails to accurately reflect pachytene piRNA deficiency. Moreover, our previously published article has comprehensively demonstrated the impact of conditional *Tdrd5* knockout on pachytene piRNA biogenesis(Ding *et al.*, 2018). Therefore, we did not include *Tdrd5* KO data in Fig. EV5. Importantly, this does not affect our conclusion that the *Tdrd5* M4 mutation does not affect pachytene piRNA biogenesis.

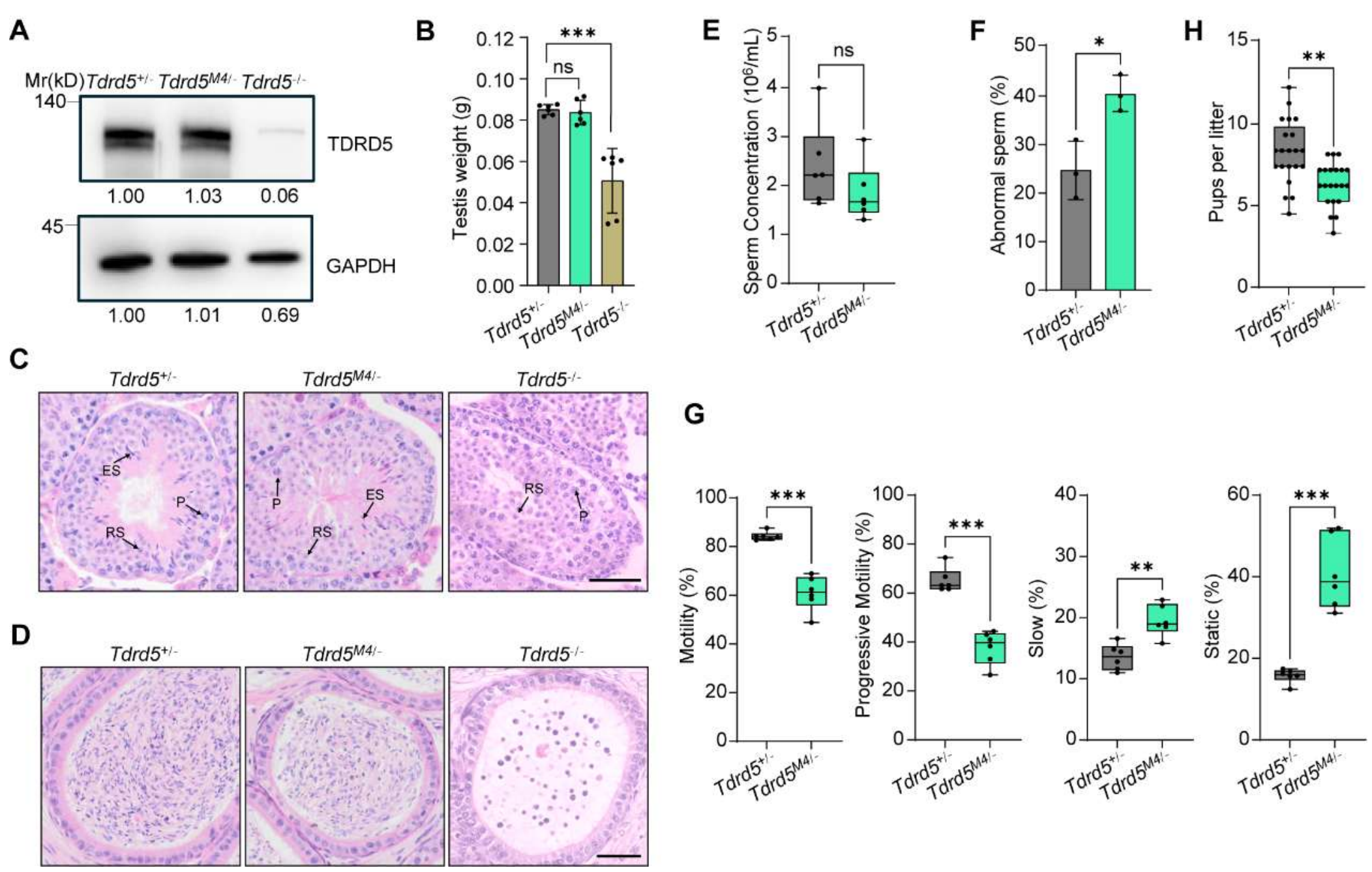

Fig. 5

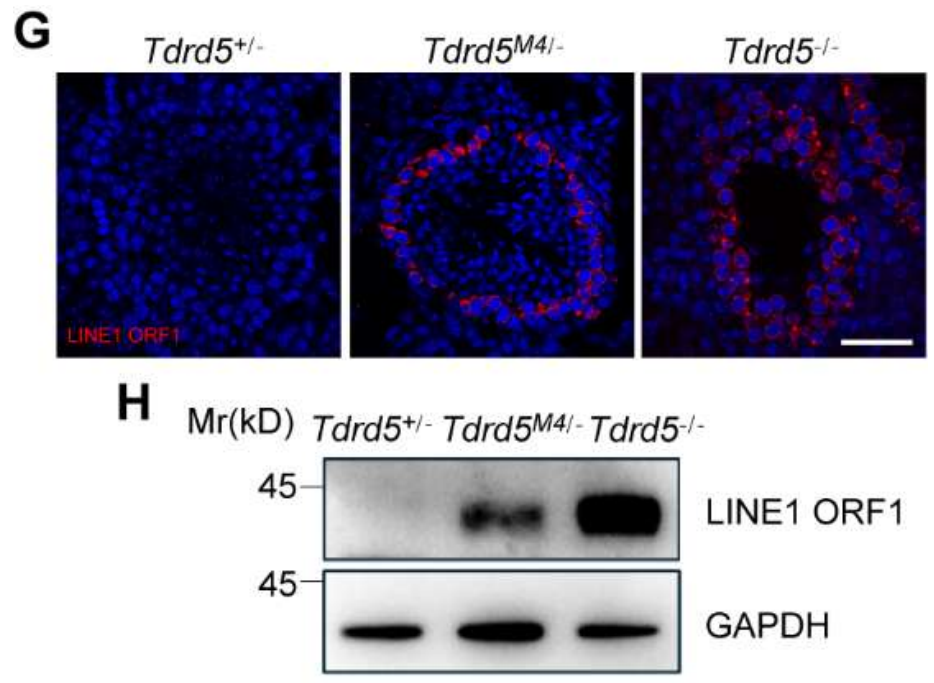

Figs. 6G and 6H

-In line 219, the genotype name "drd5M4/−" is miswritten and should be corrected to "Tdrd5M4/−".

Response: We thank the reviewer for pointing out this mistake. We have corrected it in the revised manuscript.

Major:

-It would be interesting to determine whether the LOTUS domains or IDR4 alone can form condensates in vitro. It would also be important to test whether G-rich RNA (G-quadruplex RNA TERRA) enhances phase separation in these minimal constructs.

Response: We thank the reviewer for this comment. To address this, we purified recombinant GFP-TDRD5^{L123} and GFP-TDRD5^{IDR4} and examined their phase separation potential *in vitro*. GFP-TDRD5^{IDR4} alone failed to form liquid droplets, even after the addition of G-rich RNA (TERRA). GFP-TDRD5^{L123} alone also did not form liquid droplets; instead, it produced irregular, solid-like precipitates. Notably, adding G-rich RNA further increased the amount of these precipitates rather than inducing liquid condensates. These new data demonstrate that neither the Lotus domains nor IDR4 is sufficient to drive liquid-liquid phase separation (LLPS) on its own; they must act synergistically. The Lotus domains likely provide the primary RNA-binding capacity, but this strictly requires the flexibility and multivalent weak interactions contributed by IDR4 to maintain a dynamic, liquid-like state and to prevent solid aggregation. We have now included these new data in the revised manuscript and the revised Appendix Fig. EV2I and EV2J.

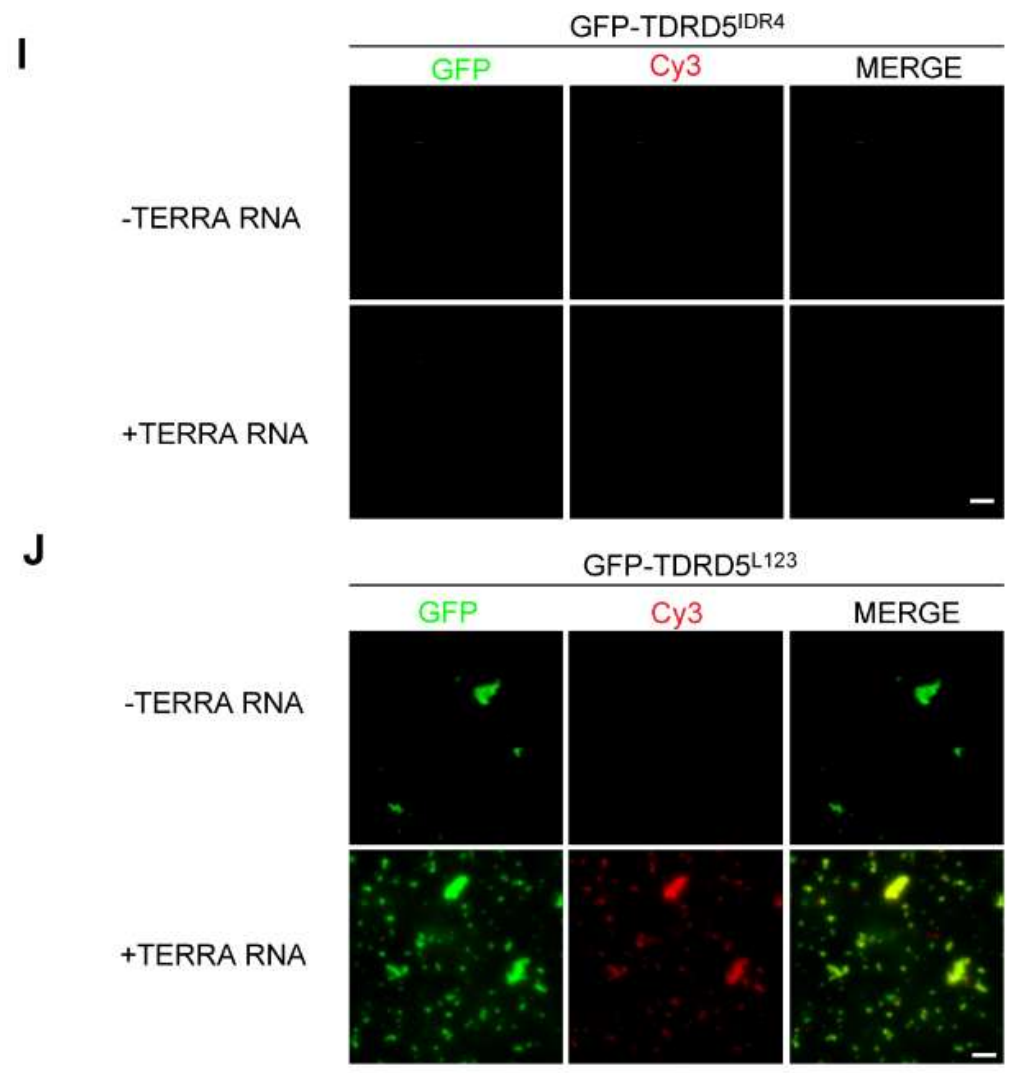

Appendix Fig. EV2I and EV2J

-For Figure 3A, since the authors already have the data, it would be valuable to present a phase diagram to summarize the salt- and concentration-dependent phase behavior.

Response: We thank the reviewer for this comment. Following the reviewer's suggestion, we have now added a phase diagram in the revised Fig. EV2D.

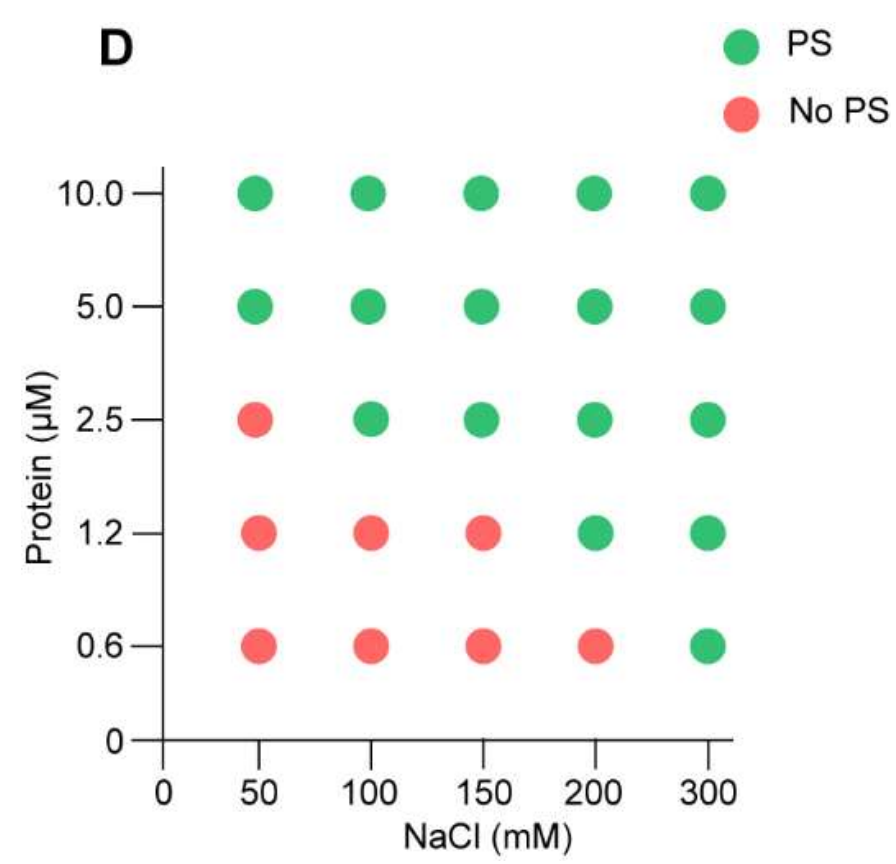

Fig. EV2D

-For Figure 3E, to make the conclusion more quantitative, the authors could estimate the saturation concentration (Csat) to quantify the effect of RNA on phase separation. Maybe you can use FCS or centrifugation-based assays for this purpose. It would also strengthen the argument to include a control panel where only RNA is added as control.

Response: We thank the reviewer for this comment. Following the reviewer's suggestion, we performed a quantitative centrifugation-based supernatant depletion assay(Conicella *et al*, 2020) to evaluate the effect of TERRA RNA on TDRD5 phase separation. The new quantitative results (Fig. EV2G) clearly demonstrate that TERRA RNA promotes TDRD5 condensation in a dose-dependent manner. As the RNA

concentration increased from 0 to 600 nM, the amount of TDRD5$^{L123+IDR4}$ remaining in the soluble fraction decreased progressively. In the absence of RNA, the soluble protein concentration was approximately 2.25 μM; upon RNA addition, it steadily decreased to a plateau of about 0.6 μM at 600 nM RNA, corresponding to a nearly four-fold reduction in the remaining protein in the supernatant. Control experiments with RNA alone showed no detectable protein signal, confirming assay specificity. We have included these quantitative data in the revised Fig. EV2G and updated the Result section accordingly.

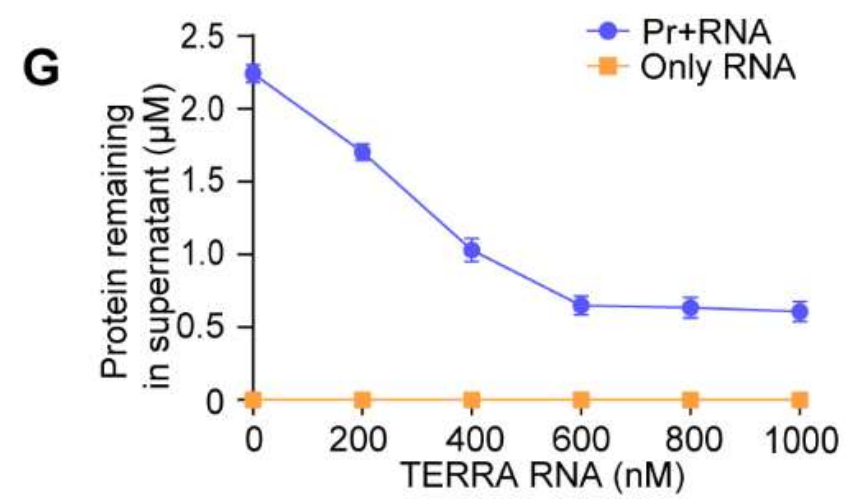

Figures EV2G

-For Figure 7E-H, the in vivo and in vitro data appear contradictory. While TDRD1 and TDRD5 do not interact strongly or colocalize in HeLa cells, TDRD1 is nonetheless recruited into TDRD5 condensates. One would expect that using the TDRD1 3GS mutant together with TDRD5 WT, which forms condensates, would be sufficient to recruit TDRD1 into these condensates, and vice versa. Perhaps the authors could add RNA to the system and test whether this better recapitulates the in vivo results. Another explanation is that the same amino acids responsible for phase separation, e.g. M4, of each protein are also responsible for their mixing behavior. In that context, it would be helpful to perform the same assay as in Figure 7H with the TDRD5 M4 mutant (at concentrations above 10 μM, where it still shows condensation).

Response: We thank the reviewer for this suggestion. Accordingly, we performed the co-condensation assay using the TDRD5$^{L123+IDR4}$-M4 mutant at an elevated concentration (15 μM) mixed with the TDRD1$^{TD2+CTD}$ fragment. As expected, the TDRD5$^{L123+IDR4}$-M4 mutant exhibited weak but detectable condensate formation at 15 μM. However, it failed to recruit the TDRD1$^{TD2+CTD}$ fragment into these condensates. We speculate that the M4 mutation markedly impairs the phase separation capacity of TDRD5. As a result, the compromised condensate network lacks the sufficient driving force to effectively partition or recruit the TDRD1$^{TD2+CTD}$ fragment. These results are fully consistent with the reviewer's hypothesis: the same amino acids that drive intact TDRD5 phase separation are also critical for establishing the proper condensate

environment required for mixing and recruitment. We have included these new data in the revised Appendix Fig. S5 and updated the manuscript accordingly.

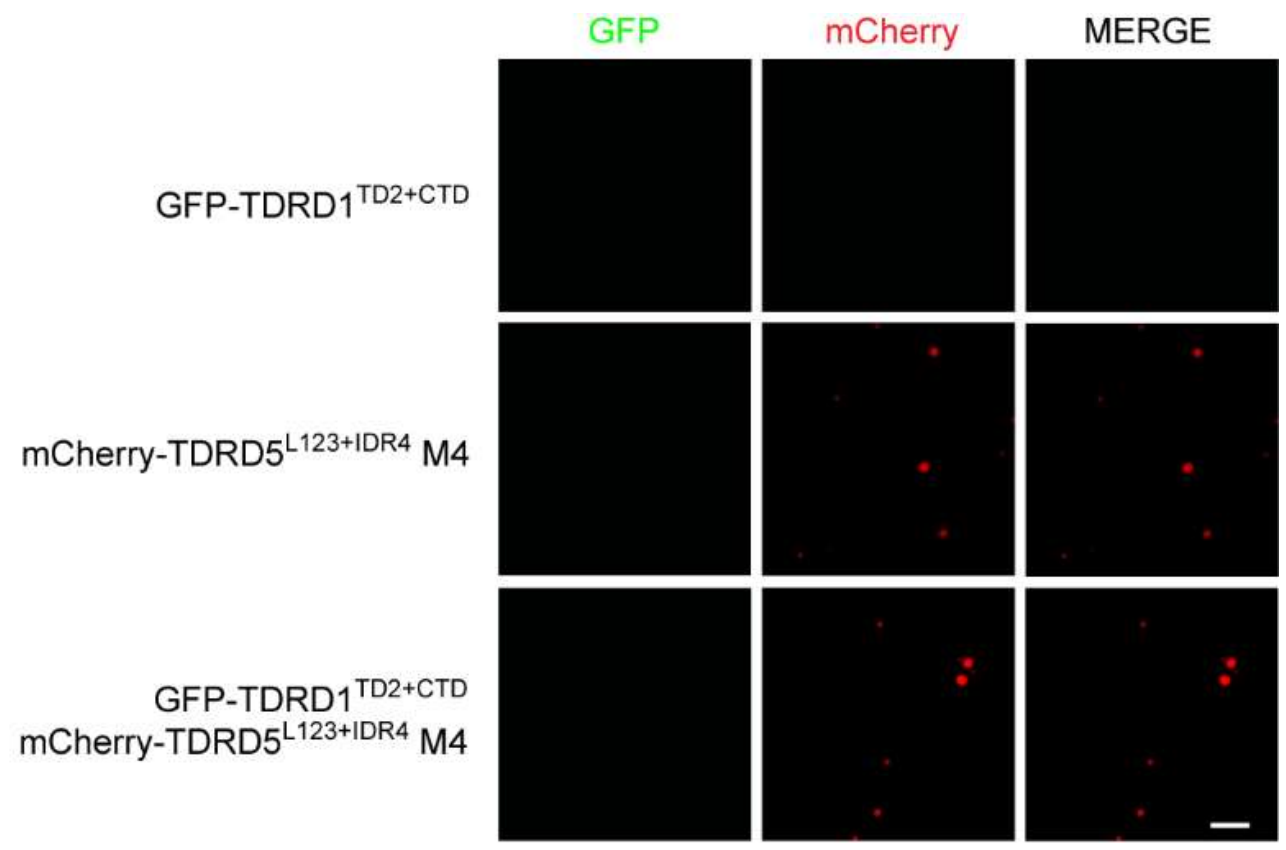

Appendix Fig. S5

Reviewer #3:

piRNAs, which suppress transposon expression, are produced in the intermitochondrial cement (IMC), a germ granule that assembles through phase separation. Previous work showed that TDRD1 undergoes phase separation to promote IMC formation. In this study, the authors demonstrate that TDRD5 also undergoes phase separation and contributes to IMC organization.

They show that TDRD5 phase separation requires cooperative action of its tandem Lotus domains and IDR4, with aromatic residues in IDR4 playing a critical role. Interaction with PIWIL2 (MILI) is required for recruitment of TDRD5 to the IMC. Notably, loss of TDRD5 phase separation significantly reduces fetal piRNA abundance in gonocytes, whereas pachytene piRNA biogenesis is largely unaffected, suggesting stage-specific roles.

Furthermore, RNA enhances TDRD5 phase separation in vitro, consistent with the reported binding of Lotus domains to G-quadruplex (G4) RNA. The mechanism identified here differs from that reported for TDRD1, highlighting mechanistic diversity in IMC assembly. Overall, the study provides physiologically relevant evidence linking phase separation to piRNA production and male fertility. While some mechanistic aspects require further clarification, the work advances our understanding of how phase separation contributes to stage-specific regulation of the piRNA pathway.

We sincerely appreciate the reviewer's positive feedback and valuable recognition of our work. We are also very grateful for the constructive suggestions provided. We have carefully considered all comments and revised the manuscript accordingly.

Major concerns

1, Line 131: The optoDroplet assay indicates that the second and third Lotus domains (L2 and L3) possess phase separation capacity. However, it remains unclear whether deletion of L2 and L3 abolishes condensation to the same extent as deletion of all three Lotus domains. A direct comparison of these mutants would clarify the relative contribution of each Lotus domain and determine whether L2 and L3 act as dominant drivers of TDRD5 condensation.

Response: We thank the reviewer for this comment. To address this, we performed the suggested experiments and directly compared the mutants. Deletion of L2 and L3 completely abolishes TDRD5 condensation, to the same extent as deletion of all three Lotus domains. This result confirms that L2 and L3 are essential for the phase separation of TDRD5. These new data have been added to the revised Appendix Fig. S2A, and the manuscript text has been updated accordingly.

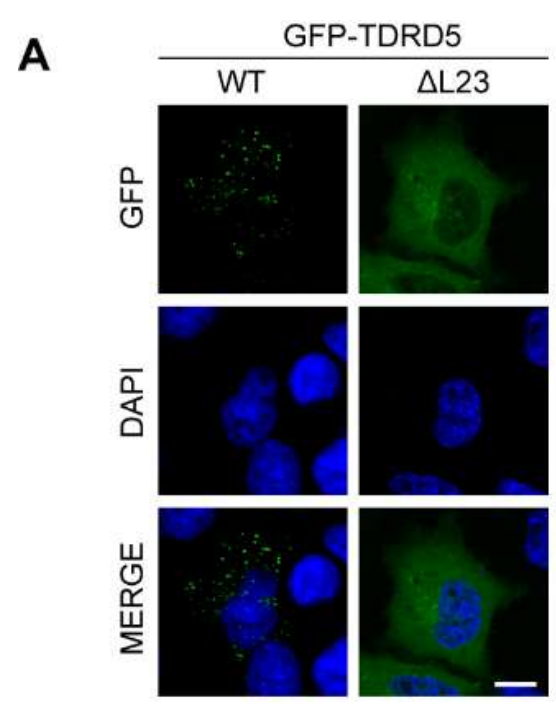

Appendix Fig. S2A

2, L133: In contrast to L2 and L3, the first Lotus domain (L1) does not exhibit phase separation capacity in the optoDroplet assay (Fig. 2E). Given that the authors provide a multi-species conservation analysis for IDR4 (Appendix Fig. S2), a similar comparative analysis of L1 versus L2/L3 (e.g., sequence alignment and conservation profiling across species) would help clarify whether specific conserved residues or multivalent features correlate with the distinct condensation behavior. Such analysis could strengthen the mechanistic interpretation of functional divergence among the Lotus domains.

Response: We thank the reviewer for this insightful comment. To address this, we performed a multi-species sequence alignment and conservation analysis for all three LOTUS domains (L1, L2, and L3). The analysis revealed that all three domains are highly conserved across mammalian species. To further uncover the sequence features underlying their distinct condensation capacities in the optoDroplet assay, we compared the amino acid compositions of L1, L2, and L3. Notably, we found that L2 and L3

contain significantly higher proportions of hydrophobic and aromatic residues than L1. It is well established that hydrophobic and aromatic residues serve as primary drivers of multivalent interactions in phase-separating proteins (e.g., via pi–pi and hydrophobic interactions). Therefore, the enrichment of these residues in L2 and L3 provides a strong mechanistic rationale for why they possess intrinsic phase separation capacity, whereas L1 does not. We believe these findings considerably enhance the validity of our data. We thank the reviewer again for their valuable input. We have now included the new data in the revised Appendix Fig. S2B-D and updated the corresponding text in the manuscript.

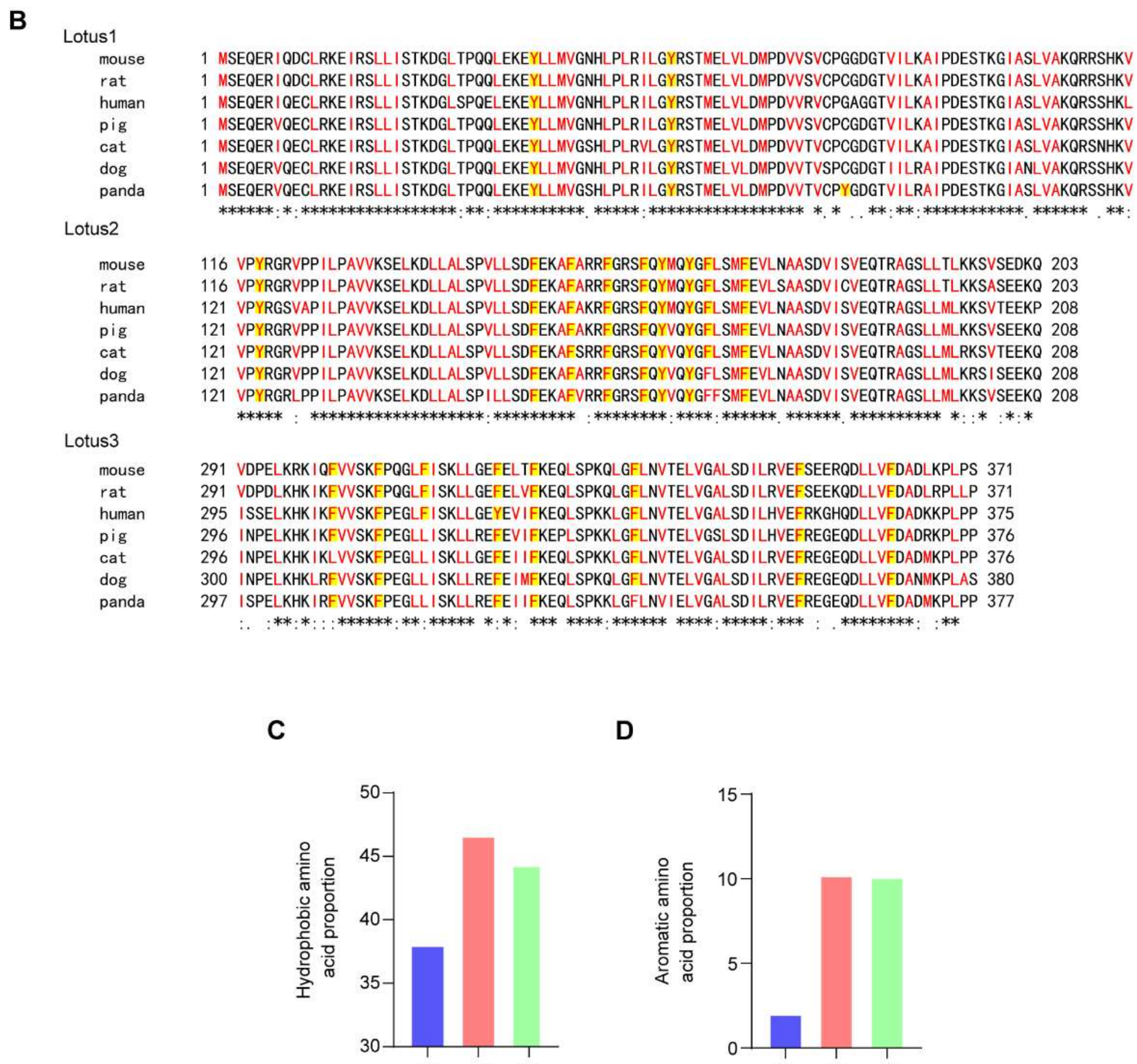

Appendix Fig. S2B-D

3, Line 141: The authors show that L123+IDR4 forms pronounced condensates (Figs. 2J and K). However, the construct containing L123 without IDR4 was not presented. This control would be necessary to demonstrate functional cooperativity between the Lotus domains and IDR4. In addition, single Lotus domain + IDR4 constructs localize to both cytoplasm and nucleus, whereas L123+IDR4 condensates appear restricted to the nucleus. What accounts for this difference in subcellular localization? Is nuclear

enrichment required for efficient condensation?

Response: We thank the reviewer for this insightful comment. We have now included the construct containing L123 without IDR4, which serves as an essential control to assess the functional cooperativity between the Lotus domains and IDR4. As expected, L123 alone fails to form condensates in cells. The new data have been added in the revised Fig. 2J and 2K.

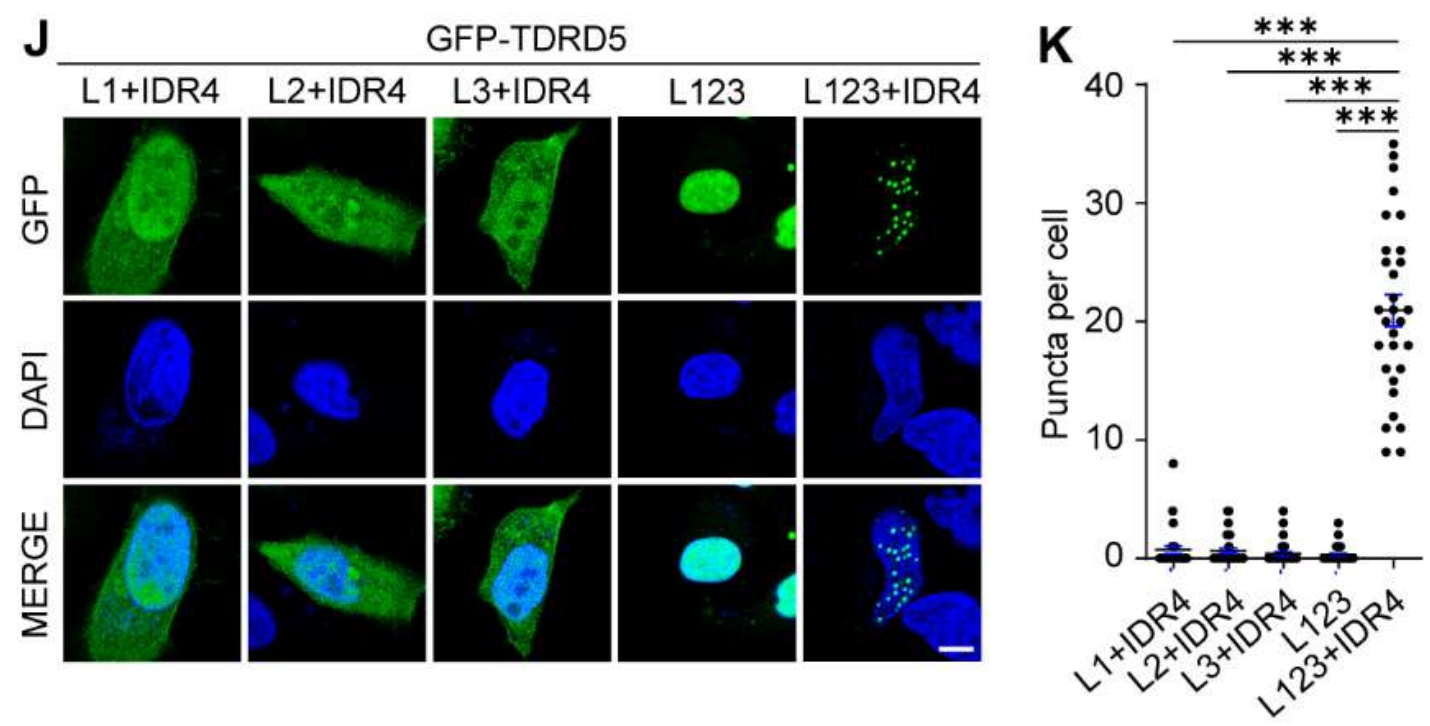

Fig. 2J and 2K

Regarding subcellular localization, we found that individual Lotus domain localizes to both the cytoplasm and the nucleus, whereas multiple tandem Lotus domains localize almost exclusively to the nucleus. We speculate that their nuclear localization is likely due to interactions with nuclear proteins or RNAs that recruit Lotus domain to the nucleus, and the tandem arrangement enhances this recruitment. However, we note that nuclear enrichment is not required for efficient condensation, as L123 (which localizes almost exclusively to the nucleus) fails to form condensates. Thus, the localization of the Lotus domains does not affect our conclusion that L123 and IDR4 cooperatively promote TDRD5 condensate formation.

4, Line 166: Addition of Cy3-labeled TERRA RNA enhances droplet formation in vitro (Fig. 3E), particularly at 200-500 nM RNA. Is there biochemical evidence that TDRD5 binds RNA within this concentration range? Does IDR4 contribute to RNA-mediated enhancement, or is the effect entirely dependent on the Lotus domains? Further clarification would help define the molecular basis of RNA-dependent phase separation.

Response: We thank the reviewer for this comment. To further assess the role of RNA in promoting TDRD5 phase separation, we performed *in vitro* droplet formation assays using a range of protein and RNA concentrations. We found that increasing either protein concentration or RNA concentration significantly enhanced TDRD5 phase separation, demonstrating that this process is strongly dependent on both protein and RNA concentrations. Therefore, TDRD5 is capable of undergoing phase separation well

beyond the previously tested 200-500 nM RNA range.

To address whether IDR4 contributes to RNA-mediated enhancement, we purified GFP-TDRD5^{IDR4} and added TERRA RNA. The results showed that TERRA RNA did not induce phase-separated droplet formation of GFP-TDRD5^{IDR4}. We further purified GFP-TDRD5^{L123} and revealed that GFP-TDRD5^{L123} alone also failed to form liquid droplets; instead, it produced irregular, solid-like precipitates. Notably, adding TERRA RNA further increased the amount of these precipitates rather than inducing liquid condensates. These new data demonstrate that neither the Lotus domains nor IDR4 is sufficient to drive liquid-liquid phase separation (LLPS) on its own; they must act synergistically. The Lotus domains provide the primary RNA-binding capacity, but this strictly requires the flexibility and multivalent weak interactions contributed by IDR4 to maintain a dynamic, liquid-like state and to prevent solid aggregation. We have now included these new data in the revised manuscript and the revised Figures EV2F, EV2I and EV2J.

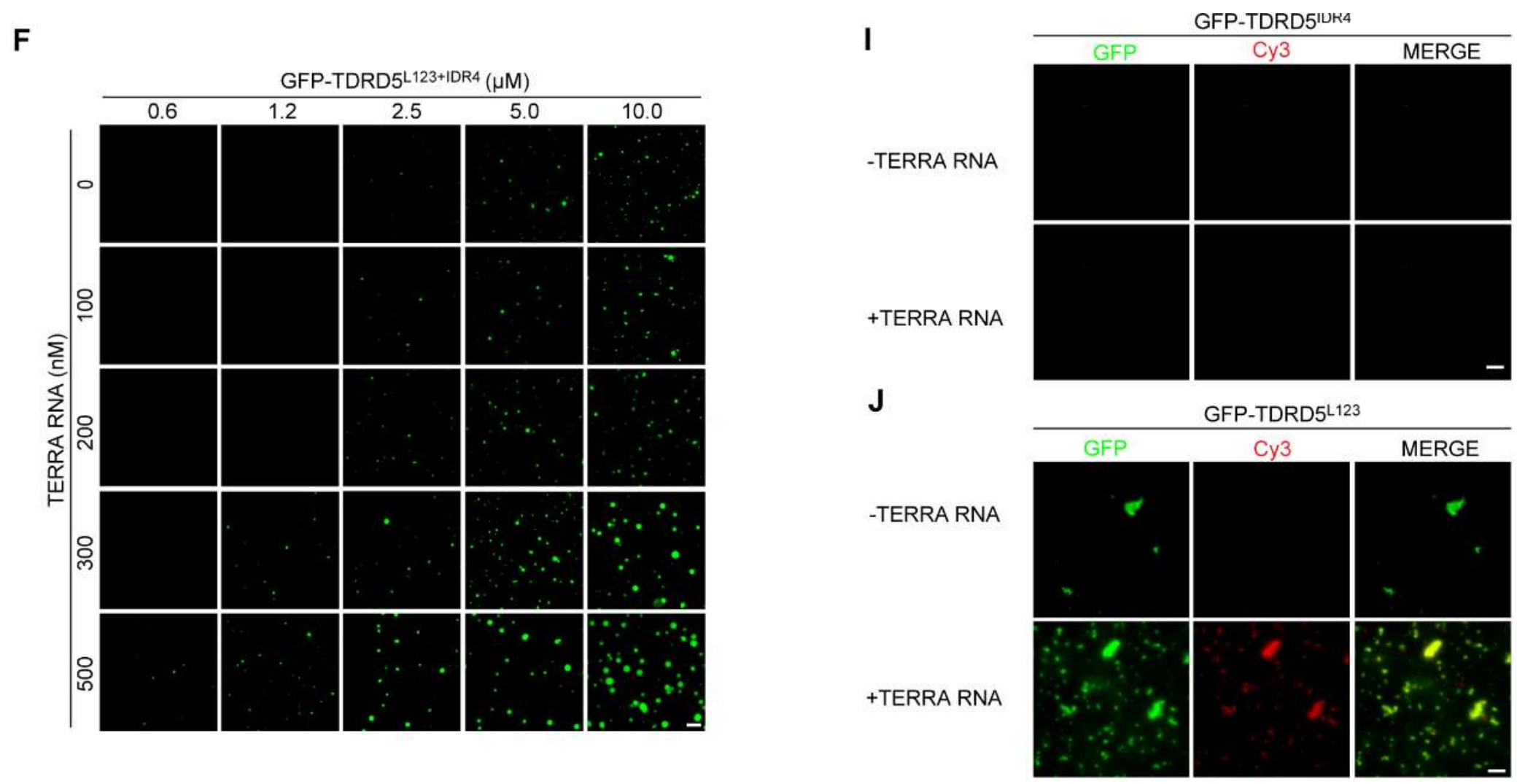

Fig. EV2F, EV2I and EV2J.

5, L257: The authors conclude that deletion of the N-terminal region or PIWI domain of PIWIL2 impairs interaction with TDRD5 (Fig. 7C). However, the observed differences appear modest. Quantitative analysis (e.g., band intensity normalization from multiple independent experiments) would strengthen this conclusion.

Response: We thank the reviewer for this comment. Accordingly, we have now performed quantification of the IP results from three independent experiments, with band intensities normalized to the corresponding immunoprecipitated bait levels. Consistent with our previously described results, deletion of the N-terminal and PAZ regions reduces the interaction between PIWIL2 and TDRD5 to some extent, but

deletion of any single domain does not completely abolish the interaction, indicating that PIWIL2 and TDRD5 have multiple interacting interfaces. The quantified data have been added to the revised Fig. 7C.

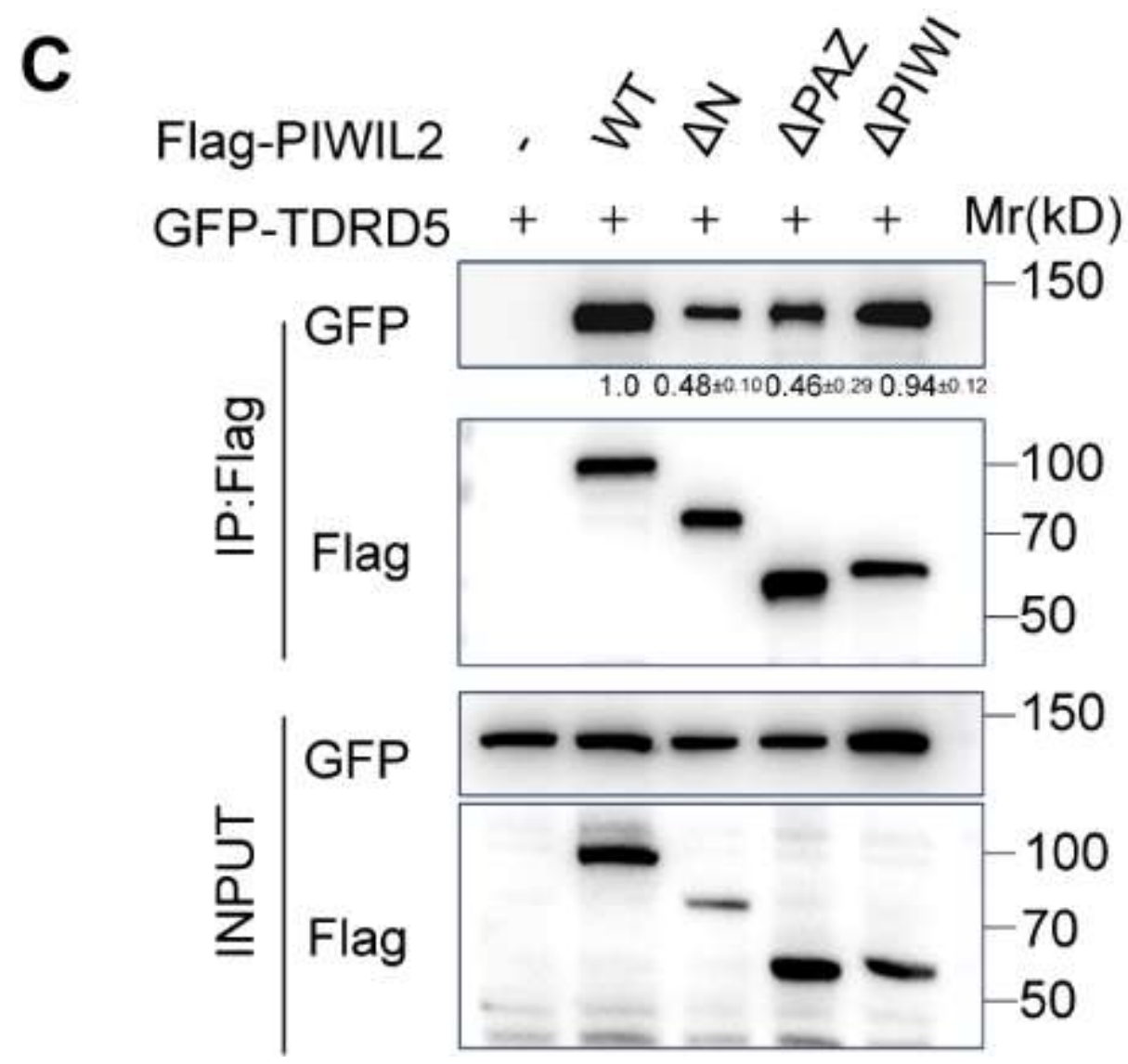

Fig. 7C

Minor concerns

1, L105: The manuscript states that 25 GFP-tagged piRNA-related proteins were constructed; however, Figure 1A appears to show 24 proteins. Please clarify this discrepancy.

Response: We thank the reviewer for pointing out this mistake. We have corrected it in the revised manuscript.

2, L75-87 and L147: A previous study on the Drosophila TDRD5 homolog Tejas (J. Cell Biol., 2023) demonstrated that its IDR contributes to granule assembly. This work should be cited and briefly discussed in the Introduction, and its relevance acknowledged at L147, to appropriately position the present findings within an evolutionary context.

Response: We thank the reviewer for this insightful comment. We agree that the study on the *Drosophila* TDRD5 homolog Tejas (J. Cell Biol., 2023), which demonstrates a role for its intrinsically disordered region (IDR) in granule assembly, is highly relevant to our work. We have now cited this study in the revised Introduction and Result and briefly discussed its findings to better position our study within an evolutionary and functional context.

3, L156: The text refers to "Figs. EV2A and 3B," but this appears to be incorrect. It

should likely read "Figs. EV2A and 2B." Please verify.

Response: We thank the reviewer for pointing out this mistake. We have corrected it in the revised manuscript.

4, L218: The sentence states that the IMC defect was less severe in "drd5M4/- and Tdrd5-/-" gonocytes. This appears to be a typographical error and should read "less severe in Tdrd5M4/- than in Tdrd5-/- gonocytes."

Response: We thank the reviewer for pointing out this mistake. We have corrected it in the revised manuscript.

5, L292: "paly" should be corrected to "play."

Response: We thank the reviewer for pointing out this mistake. We have corrected it in the revised manuscript.

6, Fig. EV1C: Purified GFP-TDRD5L123+IDR4 (WT and M4 mutant) appear to migrate slightly differently despite having identical predicted lengths. Please clarify whether this reflects conformational differences, partial degradation, or tag processing efficiency.

Response: We thank the reviewer for this insightful comment. To address this concern, we repeated the experiment under optimized and consistent electrophoresis conditions. In the revised results, the WT and M4 proteins now exhibit aligned migration patterns, consistent with their identical predicted molecular weights. We speculate that the slight migration difference observed between purified WT and M4 mutant in the original figure was due to suboptimal gel running conditions, which caused minor band misalignment. We apologize for any confusion this may have caused. The corrected results have been updated in the revised Fig. EV2C.

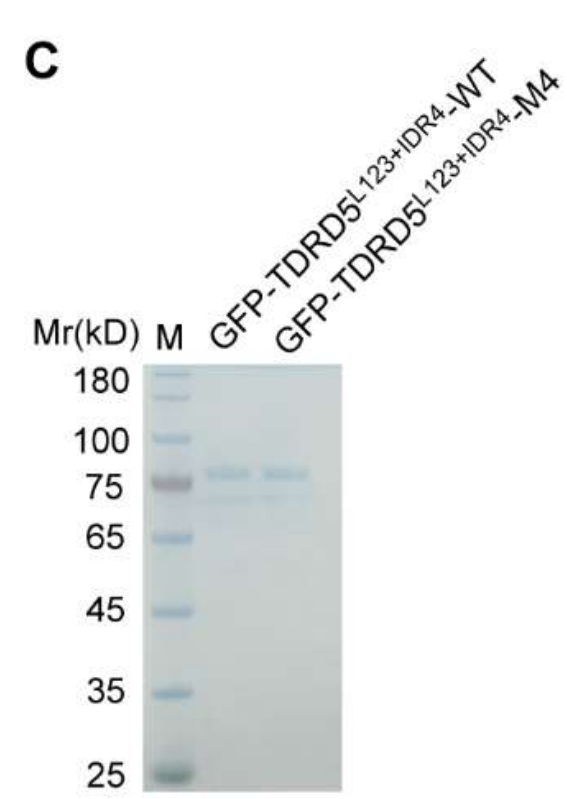

Fig. EV2C

7, L139: The optoDroplet assay shows that IDR4 robustly forms light-induced condensates (Fig. 2I). However, IDR1 also appears to form nuclear condensates. Does

IDR1 contain a nuclear localization signal? Clarification would help determine whether IDR1 has a distinct nuclear-specific role or whether its condensation is incidental.

Response: We thank the reviewer for this comment. We observed that IDR4 robustly forms puncta in the cytoplasm, whereas IDR1 can also form mild puncta in the nucleus in optoDroplet assay (Fig. 2I). Deletion of IDR4 completely abolished the puncta-forming ability of TDRD5, while deletion of IDR1 only weakly affects TDRD5 condensate formation (Fig. 2G). Therefore, we conclude that IDR4 is essential for TDRD5 condensate formation, although we cannot exclude the possibility that IDR1 plays roles in TDRD5 phase separation to some extent.

Regarding the nuclear localization of IDR1, we analyzed the TDRD5 sequence using established nuclear localization signal (NLS) prediction tools (cNLS Mapper). The analyse did not identify a strong or canonical NLS within IDR1. Thus, the nuclear localization of IDR1 observed in our optoDroplet assay is unlikely to be driven by an intrinsic NLS. In fact, fragments of several proteins that are normally localized in the cytoplasm also show nuclear localization in the optoDroplet assay(Courchaine *et al*, 2021; Shin *et al*, 2017). We speculate that this behavior may result from passive diffusion due to its size, interactions with nuclear components, or context-dependent partitioning within the nucleus. We hope that our response adequately addresses your concern.

cNLS Mapper Result

Predicted NLSs in query sequence	
ESTKGIASLVAKQRRSHKVRNSMPKGRPSICSGRVPYRGRVPPI	44

Predicted monopartite NLS		
Pos.	Sequence	Score

Predicted bipartite NLS		
Pos.	Sequence	Score

Fig. R1

References

Conicella AE, Dignon GL, Zerze GH, Schmidt HB, D'Ordine AM, Kim YC, Rohatgi R, Ayala YM, Mittal J, Fawzi NL (2020) TDP-43 α-helical structure tunes liquid-liquid phase separation and function. P Natl Acad Sci USA 117: 5883-5894

Courchaine EM, Barentine AES, Straube K, Lee DR, Bewersdorf J, Neugebauer KM

(2021) DMA-tudor interaction modules control the specificity of condensates. Cell 184: 3612-3625

Ding D, Liu J, Midic U, Wu Y, Dong K, Melnick A, Latham KE, Chen C (2018) TDRD5 binds piRNA precursors and selectively enhances pachytene piRNA processing in mice. Nature communications 9: 1-13

Shin Y, Berry J, Pannucci N, Haataja MP, Toettcher JE, Brangwynne CP (2017) Spatiotemporal Control of Intracellular Phase Transitions Using Light-Activated optoDroplets. Cell 168: 159-171

Yabuta Y, Ohta H, Abe T, Kurimoto K, Chuma S, Saitou M (2011) TDRD5 is required for retrotransposon silencing, chromatoid body assembly, and spermiogenesis in mice. The Journal of cell biology 192: 781-795

Dear Prof. Ding,

Thank you for the submission of your revised manuscript. We have now received the enclosed reports from the referees and I am happy to say that all 3 referees support the publication of your ms now. Referee 3 has a few more suggestions that I would like you to address and incorporate before we can proceed with the official acceptance of your manuscript. Please co-submit a point-by-point response with your final ms.

A few editorial requests will also need to be addressed:

- In the Data Availability Statement, the comment "All data presented are available in the main text and supplementary materials." needs to be removed.
Please add the specific URLs for dataset (CRA031313) to the data availability statement.

- The Disclosure and Competing Interest Statement should be placed after the Acknowledgments.

- The corresponding authors need to provide their institutional emails on the title page (and the system) - Miao Li and Na Jiang currently only have their personal emails on the title page, please correct.

- The author credits need to be removed from the ms file. All credits need to be entered during online ms submission.

- The REFERENCE format is not correct. et al needs to be used after 10 author names. Please use the EMBO reports reference style.

- In the author checklist please answer all questions on statistics and send us a new, completed checklist.

- You can have more than 5 EV figures and I think the Appendix can be deleted, which reduces the complexity of file types, which is often confusing. The Appendix figures are small and may be some can be combined into EV figures? We can accept 8 EV figures. The table in the Appendix file can be Table EV1 or part of the Reagents & Tools table.

* Figure Legends - Comments *

- Please note that the exact p values are not provided in the legends of 2D,H,K; 4C,E; 5B,G; 6A; EV-2B. Please provide exact values as reasonable.
- Please note that the box plots need to be defined in terms of minima, maxima, centre, bounds of box and whiskers, and percentile in the legends of figure 5G.
- Please note that the error bars are not defined in the legends of Appendix Figure S1-A,B.
- Please note that the red arrowheads are not defined in the legend of figure EV-4E; This needs to be rectified.

I would like to suggest some minor changes to the abstract that needs to be written in present tense. Do you agree with this:

piRNAs are small regulatory RNAs with critical roles in transposon silencing and regulation of gametogenesis. Phase separation is a driving force for piRNA biogenesis machinery formation; however, the specific piRNA-related proteins that mediate phase separation and how they coordinate to drive piRNA production remain incompletely understood. Through systematic screening, we show here that TDRD5 undergoes phase separation to promote piRNA biogenesis. TDRD5 condensation is mediated by its Lotus domains and an intrinsically disordered region enriched with aromatic residues. Furthermore, TDRD1 and TDRD5 colocalize and synergistically enhance each other's phase separation. TDRD5 phase separation is required for intermitochondrial cement assembly and fetal piRNA processing in fetal/neonatal gonocytes but is dispensable for pachytene piRNA biogenesis in adult mice. TDRD5 phase separation deficiency impairs male fertility in mice and may contribute to human male infertility. Our findings highlight the critical but diverse contribution of phase-separated proteins to germ granule assembly and piRNA production.

EMBO press papers are accompanied online by A) a short (1-2 sentences) summary of the findings and their significance, B) 2-3 bullet points highlighting key results and C) a synopsis image that is exactly 550 pixels wide and 300 pixels high. The synopsis image should provide a sketch of the major findings, like a graphical abstract. Please note that text needs to be readable at the final size. Please send us this information along with the final manuscript.

I look forward to seeing a final version of your manuscript as soon as possible.

Best regards,
Esther

Esther Schnapp, PhD
Senior Editor
EMBO reports

Referee #2:

The authors addressed the issues adequately. I have no further points.

Referee #3:

Overall, the authors have responded sincerely to almost all of the reviewers' concerns, and the revised manuscript is substantially improved. The study addresses a timely and important question in the field, and I think the manuscript is suitable for publication in EMBO Reports. However, two remaining issues should be addressed before publication.

1. The new data in Appendix Fig. S2A appear clear and support the authors' conclusion. However, because the authors state that deletion of L2 and L3 abolishes TDRD5 condensation to the same extent as deletion of all three Lotus domains, this should be supported by quantification. The authors should quantify the number of puncta per cell, as in Fig. 2D.

2. In the revised manuscript, lines 281-283 state that deletion of the N-terminal region or PIWI domain, but not the PAZ domain, impairs TDRD5-PIWIL2 binding'. However, this description does not seem fully consistent with the revised Fig. 7C. The authors should carefully revise the manuscript text to correctly describe the new data.

Below is the point-by-point response to comments from the reviewers.

Reviewer #3:

Overall, the authors have responded sincerely to almost all of the reviewers' concerns, and the revised manuscript is substantially improved. The study addresses a timely and important question in the field, and I think the manuscript is suitable for publication in EMBO Reports. However, two remaining issues should be addressed before publication.

We greatly appreciate the reviewer for the constructive suggestions.

1. The new data in Appendix Fig. S2A appear clear and support the authors' conclusion. However, because the authors state that deletion of L2 and L3 abolishes TDRD5 condensation to the same extent as deletion of all three Lotus domains, this should be supported by quantification. The authors should quantify the number of puncta per cell, as in Fig. 2D.

Response: We thank the reviewer for this suggestion. We have added the number of puncta per cell in the revised Figure EV3.

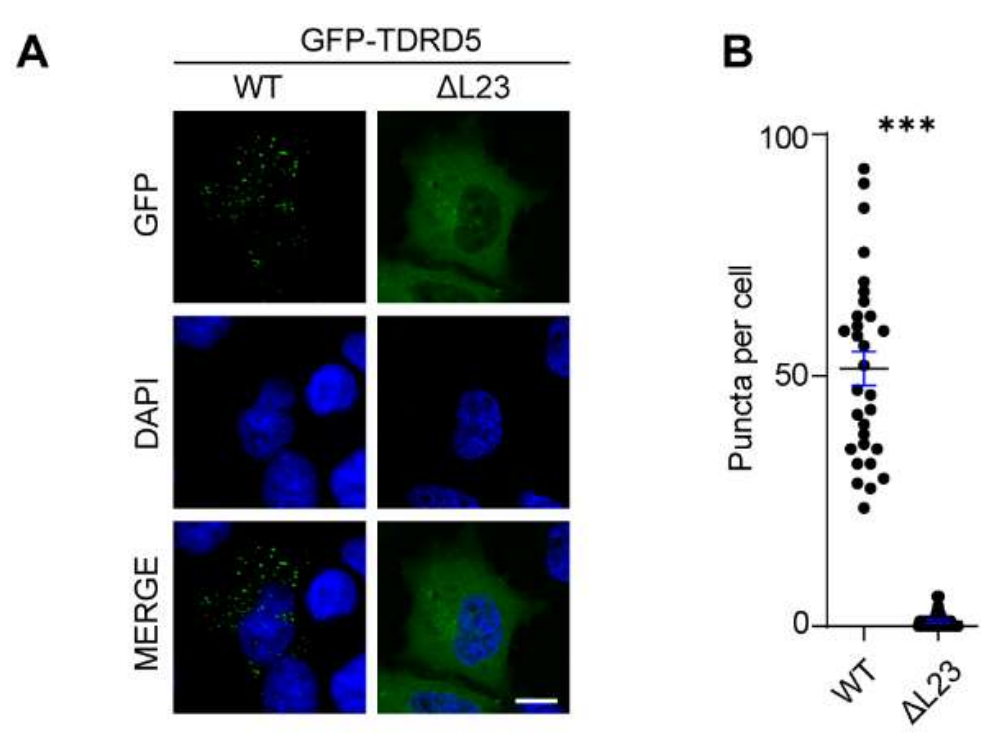

Figure EV3

2, In the revised manuscript, lines 281-283 state that deletion of the N-terminal region or PIWI domain, but not the PAZ domain, impairs TDRD5-PIWIL2 binding'. However, this description does not seem fully consistent with the revised Fig. 7C. The authors should carefully revise the manuscript text to correctly describe the new data

Response: We thank the reviewer for pointing out this mistake. We have corrected it in the revised manuscript.

Prof. Deqiang Ding
Tongji University
School of Life Sciences and Technology
China

Dear Prof. Ding,

I am very pleased to accept your manuscript for publication in the next available issue of EMBO reports. Thank you for your contribution to our journal.

Your manuscript will be processed for publication by EMBO Press. It will be copy edited and you will receive page proofs prior to publication. Please note that you will be contacted by Springer Nature Author Services to complete licensing and payment information.

You may qualify for financial assistance for your publication charges - either via a Springer Nature fully open access agreement or an EMBO initiative. Check your eligibility: https://link.springer.com/journal/44319/how-to-publish-with-us

Should you be planning a Press Release on your article, please notify the production team via this form in order to coordinate publication and release dates: https://forms.cloud.microsoft/e/eH4A8JVP6g

If you have any questions, please do not hesitate to contact the Editorial Office. Thank you for your contribution to EMBO Reports.

Best regards,
Esther

Esther Schnapp, PhD
Senior Editor
EMBO reports

>>> Please note that it is EMBO Reports policy for the transcript of the editorial process (containing referee reports and your response letter) to be published as an online supplement to each paper. If you do NOT want this, you will need to inform the Editorial Office via email immediately. More information is available here: https://link.springer.com/partners/embo-press/editorial-policies#Peer%20review

EMBO Press Author Checklist

Corresponding Author Name: Deqiang Ding	
Journal Submitted to: EMBO Reports	
Manuscript Number: EMBOR-2026-63655V1	

USEFUL LINKS FOR COMPLETING THIS FORM
The EMBO Journal - Author Guidelines
EMBO Reports - Author Guidelines
Molecular Systems Biology - Author Guidelines
EMBO Molecular Medicine - Author Guidelines

Reporting Checklist for Life Science Articles (updated January
This checklist is adapted from Materials Design Analysis Reporting (MDAR) Checklist for Authors. MDAR establishes a minimum set of requirements in transparent reporting in the life sciences (see Statement of Task: 10.31222/osf.io/9sm4x). Please follow the journal's guidelines in preparing your
Please note that a copy of this checklist will be published alongside your article.

Abridged guidelines for figures
1. Data
The data shown in figures should satisfy the following conditions:

- the data were obtained and processed according to the field's best practice and are presented to reflect the results of the experiments in an accurate and unbiased manner.
- ideally, figure panels should include only measurements that are directly comparable to each other and obtained with the same assay.
- plots include clearly labeled error bars for independent experiments and sample sizes. Unless justified, error bars should not be shown for technical
- if n<5, the individual data points from each experiment should be plotted. Any statistical test employed should be justified.
- Source Data should be included to report the data underlying figures according to the guidelines set out in the authorship guidelines on Data

2. Captions
Each figure caption should contain the following information, for each panel where they are relevant:

- a specification of the experimental system investigated (eg cell line, species name).
- the assay(s) and method(s) used to carry out the reported observations and measurements.
- an explicit mention of the biological and chemical entity(ies) that are being measured.
- an explicit mention of the biological and chemical entity(ies) that are altered/varied/perturbed in a controlled manner.
- the exact sample size (n) for each experimental group/condition, given as a number, not a range;
- a description of the sample collection allowing the reader to understand whether the samples represent technical or biological replicates (including how many animals, litters, cultures, etc.).
- a statement of how many times the experiment shown was independently replicated in the laboratory.
- definitions of statistical methods and measures:
  - common tests, such as t-test (please specify whether paired vs. unpaired), simple χ2 tests, Wilcoxon and Mann-Whitney tests, can be unambiguously identified by name only, but more complex techniques should be described in the methods section;
  - are tests one-sided or two-sided?
  - are there adjustments for multiple comparisons?
  - exact statistical test results, e.g., P values = x but not P values < x;
  - definition of 'center values' as median or average;
  - definition of error bars as s.d. or s.e.m.

Please complete ALL of the questions below.
Select "Not Applicable" only when the requested information is not relevant for your study.

Materials

Newly Created Materials	Information included in the manuscript?	In which section is the information available? (Reagents and Tools Table, Materials and Methods, Figures, Data Availability Section)
New materials and reagents need to be available; do any restrictions apply?	Not Applicable	

Antibodies	Information included in the manuscript?	In which section is the information available? (Reagents and Tools Table, Materials and Methods, Figures, Data Availability Section)
For **antibodies** provide the following information:<br>- Commercial antibodies: RRID (if possible) or supplier name, catalogue number and or/clone number<br>- Non-commercial: RRID or citation	Yes	Reagents and Tools Table

DNA and RNA sequences	Information included in the manuscript?	In which section is the information available? (Reagents and Tools Table, Materials and Methods, Figures, Data Availability Section)
**Short novel DNA or RNA including primers, probes**: provide the sequences.	Yes	Figures

Cell materials	Information included in the manuscript?	In which section is the information available? (Reagents and Tools Table, Materials and Methods, Figures, Data Availability Section)
**Cell lines:** Provide species information, strain. Provide accession number in repository **OR** supplier name, catalog number, clone number, and/**OR** RRID.	Yes	Materials and Methods
**Primary cultures:** Provide species, strain, sex of origin, genetic modification status.	Not Applicable	
Report if the cell lines were recently **authenticated** (e.g., by STR profiling) and tested for mycoplasma contamination.	Not Applicable	

Experimental animals	Information included in the manuscript?	In which section is the information available? (Reagents and Tools Table, Materials and Methods, Figures, Data Availability Section)
**Laboratory animals or Model organisms:** Provide species, strain, sex, age, genetic modification status. Provide accession number in repository **OR** supplier name, catalog number, clone number, **OR** RRID.	Yes	Materials and Methods
**Animal observed in or captured from the field:** Provide species, sex, and age where possible.	Not Applicable	
Please detail **housing and husbandry conditions**.	Yes	Materials and Methods

Plants and microbes	Information included in the manuscript?	In which section is the information available? (Reagents and Tools Table, Materials and Methods, Figures, Data Availability Section)
**Plants:** provide species and strain, ecotype and cultivar where relevant, unique accession number if available, and source (including location for collected wild specimens).	Not Applicable	
**Microbes:** provide species and strain, unique accession number if available, and source.	Not Applicable	

Human research participants	Information included in the manuscript?	In which section is the information available? (Reagents and Tools Table, Materials and Methods, Figures, Data Availability Section)
If collected and within the bounds of privacy constraints report on age, sex and gender or ethnicity for all study participants.	Not Applicable	

Core facilities	Information included in the manuscript?	In which section is the information available? (Reagents and Tools Table, Materials and Methods, Figures, Data Availability Section)
If your work benefited from core facilities, was their service mentioned in the acknowledgments section?	Not Applicable	

Design

Study protocol	Information included in the manuscript?	In which section is the information available? (Reagents and Tools Table, Materials and Methods, Figures, Data Availability Section)
If study protocol has been **pre-registered, provide DOI in the manuscript**. For clinical trials, provide the trial registration number **OR** cite DOI.	Not Applicable	
Report the **clinical trial registration number** (at ClinicalTrials.gov or equivalent), where applicable.	Not Applicable	

Laboratory protocol	Information included in the manuscript?	In which section is the information available? (Reagents and Tools Table, Materials and Methods, Figures, Data Availability Section)
Provide DOI OR other citation details if **external detailed step-by-step protocols** are available.	Not Applicable	

Experimental study design and statistics	Information included in the manuscript?	In which section is the information available? (Reagents and Tools Table, Materials and Methods, Figures, Data Availability Section)
Include a statement about **sample size** estimate even if no statistical methods were used.	Yes	Materials and Methods, Figure legends
Were any steps taken to minimize the effects of subjective bias when allocating animals/samples to treatment (e.g. **randomization procedure**)? If yes, have they been described?	Not Applicable	
Include a statement about **blinding** even if no blinding was done.	Yes	Materials and Methods
Describe **inclusion/exclusion criteria** if samples or animals were excluded from the analysis. Were the criteria pre-established?<br><br>If sample or data points were omitted from analysis, report if this was due to attrition or intentional exclusion and provide justification.	Not Applicable	
For every figure, are **statistical tests** justified as appropriate? Do the data meet the assumptions of the tests (e.g., normal distribution)? Describe any methods used to assess it. Is there an estimate of variation within each group of data? **I**s the variance similar between the groups that are being statistically compared?	Yes	Materials and Methods, Figure legends

Sample definition and in-laboratory replication	Information included in the manuscript?	In which section is the information available? (Reagents and Tools Table, Materials and Methods, Figures, Data Availability Section)
In the figure legends: state number of times the experiment was **replicated** in laboratory.	Yes	Figures
In the figure legends: define whether data describe **technical or biological replicates**.	Yes	Figures

Ethics

Ethics	Information included in the manuscript?	In which section is the information available? (Reagents and Tools Table, Materials and Methods, Figures, Data Availability Section)
Studies involving **human participants**: State details of **authority granting ethics approval** (IRB or equivalent committee(s), provide reference number for approval.	Not Applicable	
Studies involving **human participants**: Include a statement confirming that **informed consent** was obtained from all subjects and that the experiments conformed to the principles set out in the WMA Declaration of Helsinki and the Department of Health and Human Services Belmont Report.	Not Applicable	
Studies involving **human participants:** For publication of **patient photos**, include a statement confirming that consent to publish was obtained.	Not Applicable	
Studies involving experimental **animals**: State details of **authority granting ethics approval** (IRB or equivalent committee(s), provide reference number for approval. Include a statement of compliance with ethical regulations.	Yes	Materials and Methods
Studies involving **specimen and field samples:** State if relevant **permits** obtained, provide details of authority approving study; if none were required, explain why.	Not Applicable	

Dual Use Research of Concern (DURC)	Information included in the manuscript?	In which section is the information available? (Reagents and Tools Table, Materials and Methods, Figures, Data Availability Section)
Could your study fall under dual use research restrictions? Please check biosecurity documents and list of **select agents and toxins** (CDC): https://www.selectagents.gov/sat/list.htm	Not Applicable	
If you used a select agent, is the security level of the lab appropriate and reported in the manuscript?	Not Applicable	
If a study is subject to dual use research of concern regulations, is the name of the **authority granting approval and reference number** for the regulatory approval provided in the manuscript?	Not Applicable	

Reporting

The MDAR framework recommends adoption of discipline-specific guidelines, established and endorsed through community initiatives. Journals have their own policy about requiring specific guidelines and recommendations to complement MDAR.

Adherence to community standards	Information included in the manuscript?	In which section is the information available? (Reagents and Tools Table, Materials and Methods, Figures, Data Availability Section)
State if relevant guidelines or checklists (e.g., **ICMJE, MIBBI, ARRIVE, PRISMA**) have been followed or provided.	Not Applicable	
For **tumor marker prognostic studies,** we recommend that you follow the **REMARK** reporting guidelines (see link list at top right). See author guidelines, under 'Reporting Guidelines'. Please confirm you have followed these guidelines.	Not Applicable	
For **phase II and III randomized controlled trials**, please refer to the **CONSORT** flow diagram (see link list at top right) and submit the CONSORT checklist (see link list at top right) with your submission. See author guidelines, under 'Reporting Guidelines'. Please confirm you have submitted this list.	Not Applicable	

Data Availability

Data availability	Information included in the manuscript?	In which section is the information available? (Reagents and Tools Table, Materials and Methods, Figures, Data Availability Section)
Have **primary datasets** been deposited according to the journal's guidelines (see 'Data Deposition' section) and the respective accession numbers provided in the Data Availability Section?	Yes	Data availability
Were **human clinical and genomic datasets** deposited in a public access-controlled repository in accordance to ethical obligations to the patients and to the applicable consent agreement?	Not Applicable	
Are **computational models** that are central and integral to a study available without restrictions in a machine-readable form? Were the relevant accession numbers or links provided?	Not Applicable	
If publicly available data were reused, provide the respective **data citations in the reference list**.	Not Applicable	
