# Supplementary material for: TDRD5 facilitates piRNA biogenesis and male fertility through phase separation
Source: EMBO Rep. 2026 Aug 4;27(18):5292–316. doi: 10.1038/s44319-026-00890-6 (PMC13601554; doi:10.1038/s44319-026-00890-6)
Supplement: Supplementary file 3 [file 44319_2026_890_MOESM1_ESM.docx]

**Table EV1. Plasmid used in this study.**

| **Construct** | **Source** | **Uniprot ID** | **Insert Sequence Region** | **Backbone** |
| --- | --- | --- | --- | --- |
| Flag | (Gao, Jing et al., 2024) | N/A | N/A | pcDNA3-Flag |
| Flag-TDRD1 | (Gao et al., 2024) | [Q99MV1](https://www.uniprot.org/uniprotkb/Q99MV1/entry) | 1-1172 | pcDNA3-Flag |
| Flag-PIWIL2 | (Gao, Chen et al., 2025) | Q8CDG1 | 1-971 | pcDNA3-Flag |
| Flag-PIWIL2-RK | (Gao et al., 2025) | Q8CDG1 | 9/39/45/74/95/100  /144/156/163 R→K | pcDNA3-Flag |
| Flag-PIWIL2-ΔN | (Gao et al., 2025) | Q8CDG1 | 201-971 | pcDNA3-Flag |
| Flag-PIWIL2-ΔPAZ | (Gao et al., 2025) | Q8CDG1 | 1-200; 601-971 | pcDNA3-Flag |
| Flag-PIWIL2-ΔPIWI | (Gao et al., 2025) | Q8CDG1 | 1-600 | pcDNA3-Flag |
| mCherry-TDRD5 | This paper | [Q5VCS6](https://www.uniprot.org/uniprotkb/Q5VCS6/entry) | 1-1040 | pmCherry-C1 |
| mCherry-TDRD5-M4 | This paper | [Q5VCS6](https://www.uniprot.org/uniprotkb/Q5VCS6/entry) | W793G;W797G;F815G;Y825G | pmCherry-C1 |
| RFP-ASZ1 | (Gao et al., 2025) | Q8VD46 | 1-475 | pcDNA3-RFP |
| TDRKH-GFP | (Gao et al., 2024) | Q80VL1 | 1-560 | pEGFP-N1 |
| mitoPLD-GFP | (Gao et al., 2024) | Q5SWZ9 | 1-221 | pEGFP-N1 |
| TDRD5-GFP | This paper | [Q5VCS6](https://www.uniprot.org/uniprotkb/Q5VCS6/entry) | 1-1040 | pEGFP-N1 |
| GPAT2-GFP | (Gao et al., 2024) | Q14DK4 | 1-801 | pEGFP-N1 |
| GFP-ASZ1 | (Gao et al., 2024) | Q8VD46 | 1-475 | pEGFP-C1 |
| GFP-PNLDC1 | (Gao et al., 2024) | B2RXZ1 | 1-531 | pEGFP-C1 |
| GFP-PIWIL2 | (Gao et al., 2024) | Q8CDG1 | 1-971 | pEGFP-C1 |
| GFP-PIWIL1 | (Ding, Liu et al., 2019) | Q9JMB7 | 1-862 | pEGFP-C1 |
| GFP-PIWIL4 | (Ding et al., 2019) | Q8CGT6 | 1-848 | pEGFP-C1 |
| GFP-TDRD1 | (Gao et al., 2024) | [Q99MV1](https://www.uniprot.org/uniprotkb/Q99MV1/entry) | 1-1172 | pEGFP-C1 |
| GFP-TDRD1-3GS | (Gao et al., 2024) | [Q99MV1](https://www.uniprot.org/uniprotkb/Q99MV1/entry) | 1149-1154:  ILLFLL→GSGSGS | pEGFP-C1 |
| GFP-RNF17 | (Wei, Jing et al., 2023) | Q99MV7 | 1-1640 | pEGFP-C1 |
| GFP-TDRD5 | (Ding, Wei et al., 2020) | [Q5VCS6](https://www.uniprot.org/uniprotkb/Q5VCS6/entry) | 1-1040 | pEGFP-C1 |
| GFP-TDRD5-ISFORM2 | This paper | [Q5VCS6](https://www.uniprot.org/uniprotkb/Q5VCS6/entry) | 1-131;228-1040 | pEGFP-C1 |
| GFP-TDRD5-ΔL1 | This paper | [Q5VCS6](https://www.uniprot.org/uniprotkb/Q5VCS6/entry) | 100-1040 | pEGFP-C1 |
| GFP-TDRD5-ΔL3 | This paper | [Q5VCS6](https://www.uniprot.org/uniprotkb/Q5VCS6/entry) | 1-290;366-1040 | pEGFP-C1 |
| GFP-TDRD5-ΔL23 | This paper | [Q5VCS6](https://www.uniprot.org/uniprotkb/Q5VCS6/entry) | 1-114;372-1040 | pEGFP-C1 |
| GFP-TDRD5-ΔL123 | This paper | [Q5VCS6](https://www.uniprot.org/uniprotkb/Q5VCS6/entry) | 350-1040 | pEGFP-C1 |
| GFP-TDRD5-ΔTD | This paper | [Q5VCS6](https://www.uniprot.org/uniprotkb/Q5VCS6/entry) | 1-453;672-1040 | pEGFP-C1 |
| GFP-TDRD5-ΔIDR1 | This paper | [Q5VCS6](https://www.uniprot.org/uniprotkb/Q5VCS6/entry) | 1-81;126-1040 | pEGFP-C1 |
| GFP-TDRD5-ΔIDR2 | This paper | [Q5VCS6](https://www.uniprot.org/uniprotkb/Q5VCS6/entry) | 1-191;275-1040 | pEGFP-C1 |
| GFP-TDRD5-ΔIDR3 | This paper | [Q5VCS6](https://www.uniprot.org/uniprotkb/Q5VCS6/entry) | 1-366;455-1040 | pEGFP-C1 |
| GFP-TDRD5-ΔIDR4 | This paper | [Q5VCS6](https://www.uniprot.org/uniprotkb/Q5VCS6/entry) | 1-707;829-1040 | pEGFP-C1 |
| GFP-TDRD5-ΔIDR5 | This paper | [Q5VCS6](https://www.uniprot.org/uniprotkb/Q5VCS6/entry) | 1-849;976-1040 | pEGFP-C1 |
| GFP-TDRD5^L1+IDR4^ | This paper | [Q5VCS6](https://www.uniprot.org/uniprotkb/Q5VCS6/entry) | 1-100;708-828 | pEGFP-C1 |
| GFP-TDRD5^L2+IDR4^ | This paper | [Q5VCS6](https://www.uniprot.org/uniprotkb/Q5VCS6/entry) | 119-200;708-828 | pEGFP-C1 |
| GFP-TDRD5^L3+IDR4^ | This paper | [Q5VCS6](https://www.uniprot.org/uniprotkb/Q5VCS6/entry) | 290-370;708-828 | pEGFP-C1 |
| GFP-TDRD5^L23+IDR4^ | This paper | [Q5VCS6](https://www.uniprot.org/uniprotkb/Q5VCS6/entry) | 117-371;708-828 | pEGFP-C1 |
| GFP-TDRD5^L123+IDR4^ | This paper | [Q5VCS6](https://www.uniprot.org/uniprotkb/Q5VCS6/entry) | 1-380;708-828 | pEGFP-C1 |
| GFP-TDRD5-M2-1 | This paper | [Q5VCS6](https://www.uniprot.org/uniprotkb/Q5VCS6/entry) | Y708G;Y731G | pEGFP-C1 |
| GFP-TDRD5-M2-2 | This paper | [Q5VCS6](https://www.uniprot.org/uniprotkb/Q5VCS6/entry) | W760G;W793G | pEGFP-C1 |
| GFP-TDRD5-M3 | This paper | [Q5VCS6](https://www.uniprot.org/uniprotkb/Q5VCS6/entry) | Y708G;Y731G;W760G | pEGFP-C1 |
| GFP-TDRD5-M4 | This paper | [Q5VCS6](https://www.uniprot.org/uniprotkb/Q5VCS6/entry) | W793G;W797G;F815G;Y825G | pEGFP-C1 |
| GFP-TDRD5-M7 | This paper | [Q5VCS6](https://www.uniprot.org/uniprotkb/Q5VCS6/entry) | Y708G;Y731G;W760G;W793G;W797G;F815G;Y825G | pEGFP-C1 |
| GFP-hTDRD5 | This paper | Q8NAT2 | 1-1035 | pEGFP-C1 |
| GFP-hTDRD5-M4 | This paper | Q8NAT2 | W794G;W798G;F816G;Y826G | pEGFP-C1 |
| GFP-hTDRD5-E765* | This paper | Q8NAT2 | E765* | pEGFP-C1 |
| GFP-TDRD6 | (Wei et al., 2023) | P61407 | 1-2134 | pEGFP-C1 |
| GFP-TDRD7 | (Gao et al., 2025) | [Q8K1H1](https://www.uniprot.org/uniprotkb/Q8K1H1/entry) | 1-1086 | pEGFP-C1 |
| GFP-TDRD9 | (Gao et al., 2025) | Q14BI7 | 1-1383 | pEGFP-C1 |
| GFP-TDRD12 | (Gao et al., 2025) | Q9CWU0 | 1-1277 | pEGFP-C1 |
| GFP-MVH | (Gao et al., 2025) | Q61496 | 1-702 | pEGFP-C1 |
| GFP-MOV10L1 | (Wei et al., 2023) | Q99MV5 | 1-1187 | pEGFP-C1 |
| GFP-FKBP6 | (Gao et al., 2025) | Q91XW8 | 1-327 | pEGFP-C1 |
| GFP-MAEL | (Gao et al., 2025) | Q8BVN9 | 1-434 | pEGFP-C1 |
| GFP-GTSF1 | (Gao et al., 2025) | Q9DAN6 | 1-167 | pEGFP-C1 |
| GFP-HENMT1 | (Gao et al., 2025) | Q8CAE2 | 1-395 | pEGFP-C1 |
| GFP-EXD1 | (Gao et al., 2025) | Q8CDF7 | 1-570 | pEGFP-C1 |
| GFP-HSP90α | (Gao et al., 2025) | P07901 | 1-733 | pEGFP-C1 |
| GFP-ADAD2 | (Gao et al., 2025) | Q9D5P4 | 1-561 | pEGFP-C1 |
| GFP-TAPAS | This paper | Q0E910 | 1-1222 | pEGFP-C1 |
| GFP-TEJAS | This paper | A1Z9P1 | 1-559 | pEGFP-C1 |
| pCRY2PHR-mCherry | (Zhang, Fan et al., 2019) | N/A | N/A | pCRY2PHR-mCherry |
| CRY2-mCherry-FUS-IDR | (Gao et al., 2024) | P56959 | 1-276 | pCRY2PHR-mCherry |
| CRY2-mCherry-TDRD5^L1^ | This paper | [Q5VCS6](https://www.uniprot.org/uniprotkb/Q5VCS6/entry) | 1-100 | pCRY2PHR-mCherry |
| CRY2-mCherry-TDRD5^L2^ | This paper | [Q5VCS6](https://www.uniprot.org/uniprotkb/Q5VCS6/entry) | 115-203 | pCRY2PHR-mCherry |
| CRY2-mCherry-TDRD5^L3^ | This paper | [Q5VCS6](https://www.uniprot.org/uniprotkb/Q5VCS6/entry) | 283-371 | pCRY2PHR-mCherry |
| CRY2-mCherry-TDRD5^TD^ | This paper | [Q5VCS6](https://www.uniprot.org/uniprotkb/Q5VCS6/entry) | 452-671 | pCRY2PHR-mCherry |
| CRY2-mCherry-TDRD5^IDR1^ | This paper | [Q5VCS6](https://www.uniprot.org/uniprotkb/Q5VCS6/entry) | 82-125 | pCRY2PHR-mCherry |
| CRY2-mCherry-TDRD5^IDR2^ | This paper | [Q5VCS6](https://www.uniprot.org/uniprotkb/Q5VCS6/entry) | 194-274 | pCRY2PHR-mCherry |
| CRY2-mCherry-TDRD5^IDR3^ | This paper | [Q5VCS6](https://www.uniprot.org/uniprotkb/Q5VCS6/entry) | 367-454 | pCRY2PHR-mCherry |
| CRY2-mCherry-TDRD5^IDR4^ | This paper | [Q5VCS6](https://www.uniprot.org/uniprotkb/Q5VCS6/entry) | 707-828 | pCRY2PHR-mCherry |
| CRY2-mCherry-TDRD5^IDR4^-M3 | This paper | [Q5VCS6](https://www.uniprot.org/uniprotkb/Q5VCS6/entry) | 707-828 (Y708G;Y731G;W760G) | pCRY2PHR-mCherry |
| CRY2-mCherry-TDRD5^IDR4^-M4 | This paper | [Q5VCS6](https://www.uniprot.org/uniprotkb/Q5VCS6/entry) | 707-828 (W793G;W797G;F815G;Y825G) | pCRY2PHR-mCherry |
| CRY2-mCherry-TDRD5^IDR4^-M7 | This paper | [Q5VCS6](https://www.uniprot.org/uniprotkb/Q5VCS6/entry) | 707-828 (Y708G;Y731G;W760G;W793G;W797G;F815G;Y825G) | pCRY2PHR-mCherry |
| CRY2-mCherry-TDRD5^IDR5^ | This paper | [Q5VCS6](https://www.uniprot.org/uniprotkb/Q5VCS6/entry) | 863-984 | pCRY2PHR-mCherry |
| GST-GFP-TDRD5^L123+IDR4^ | This paper | [Q5VCS6](https://www.uniprot.org/uniprotkb/Q5VCS6/entry) | 1-380;708-828 | PGEX-6P-1-GFP |
| GST-GFP-TDRD5^L123+IDR4^-M4 | This paper | [Q5VCS6](https://www.uniprot.org/uniprotkb/Q5VCS6/entry) | 1-380;708-828 (W793G;W797G;F815G;Y825G) | PGEX-6P-1-GFP |
| GST-mCherry-TDRD5^L123+IDR4^ | This paper | [Q5VCS6](https://www.uniprot.org/uniprotkb/Q5VCS6/entry) | 1-380;708-828 | PGEX-6P-1-mCherry |
| GST-mCherry-TDRD5^L123+IDR4 M4^ | This paper | [Q5VCS6](https://www.uniprot.org/uniprotkb/Q5VCS6/entry) | 1-380;708-828 (W793G;W797G;F815G;Y825G) | PGEX-6P-1-mCherry |
| HIS-GFP-TDRD1^TD2+CTD^ | (Gao et al., 2024) | Q99MV1 | 461-680;1080-1172 | pET-28a-GFP |
| HIS-GFP-TDRD5^L123^ | This paper | [Q5VCS6](https://www.uniprot.org/uniprotkb/Q5VCS6/entry) | 1-380 | pET-28a-GFP |
| HIS-GFP-TDRD5^IDR4^ | This paper | [Q5VCS6](https://www.uniprot.org/uniprotkb/Q5VCS6/entry) | 708-828 | pET-28a-GFP |
| GFP-TDRD5^L123^ | This paper | [Q5VCS6](https://www.uniprot.org/uniprotkb/Q5VCS6/entry) | 1-380 | pEGFP-C1 |

**Reference**

Ding D, Liu J, Dong K, Melnick AF, Latham KE, Chen C (2019) Mitochondrial membrane-based initial separation of MIWI and MILI functions during pachytene piRNA biogenesis. Nucleic Acids Res 47: 2594-2608

Ding D, Wei C, Dong K, Liu J, Stanton A, Xu C, Min J, Hu J, Chen C (2020) LOTUS domain is a novel class of G-rich and G-quadruplex RNA binding domain. Nucleic Acids Res 48: 9262-9272

Gao J, Chen C, Shang G, Yu W, Zhao T, Zhang Y, Chen C, Ding D (2025) PIWI proteins tether the piRNA biogenesis machinery to mitochondria during mammalian spermatogenesis. The EMBO journal

Gao J, Jing J, Shang G, Chen C, Duan M, Yu W, Wang K, Luo J, Song M, Chen K, Chen C, Zhang T, Ding D (2024) TDRD1 phase separation drives intermitochondrial cement assembly to promote piRNA biogenesis and fertility. Dev Cell 59: 2704-2718.e6

Wei C, Jing J, Yan X, Mann JM, Geng R, Xie H, Demireva EY, Hess RA, Ding D, Chen C (2023) MIWI N-terminal RG motif promotes efficient pachytene piRNA production and spermatogenesis independent of LINE1 transposon silencing. PLoS Genet 19: e1011031

Zhang P, Fan B, Yang P, Temirov J, Messing J, Kim HJ, Taylor JP (2019) Chronic optogenetic induction of stress granules is cytotoxic and reveals the evolution of ALS-FTD pathology. eLife 8
